# Renal Function–Guided Valganciclovir Dosing and Ganciclovir Exposure Variability: A Population Pharmacokinetic Simulation Study

**DOI:** 10.1111/bcpt.70329

**Published:** 2026-11-05

**Authors:** Franck Maizaud, Marc Labriffe, Hamza Sayadi, Stéphanie Belaiche, Pierre Marquet, Jean‐Baptiste Woillard, Caroline Monchaud

**Affiliations:** ^1^ Department of Pharmacology, Toxicology and Pharmacovigilance CHU Limoges Limoges France; ^2^ INSERM UMR‐1248 “Pharmacology & Transplantation” Limoges France; ^3^ University Hospital Federation “Survival Optimization in Organ Transplantation” (FHU SUPORT) Limoges France; ^4^ Institute of Pharmacy CHU Lille Lille France; ^5^ Department of Gastroenterology CHU Lille Lille France; ^6^ METRICS Research Laboratory (ULR 2694) Lille University Lille France

**Keywords:** cytomegalovirus prophylaxis, ganciclovir exposure, kidney transplantation, model‐informed precision dosing, population pharmacokinetics, renal function estimation

## Abstract

**Background:**

Ganciclovir (GCV) exposure after standard valganciclovir (VGCV) prophylaxis is highly variable. Guidelines recommend Cockcroft–Gault for renal dose adjustment. We evaluated whether Cockcroft–Gault‐based dosing achieves the target prophylactic exposure (AUC_0‐24h:_ 40–60 mg.h/L) and whether MDRD or CKD‐EPI alters exposure.

**Methods:**

Using a validated population pharmacokinetic model, we simulated 10 000 adult solid organ transplant recipients. VGCV doses were assigned according to SmPC renal function categories based on Cockcroft–Gault, MDRD or CKD‐EPI. Patient reclassification, target attainment and exposure around dosing thresholds (25, 40 and 60 mL/min) were assessed.

**Results:**

MDRD and CKD‐EPI reclassified 45.1% and 39.9% of patients, mostly downward, lowering target attainment (20.1% and 22.7% vs. 30.8% with Cockcroft–Gault). Target attainment never exceeded 40% in any category, and underexposure predominated, reaching up to 94.1% in the most impaired renal function categories. Target attainment was consistently lower just below each threshold, and the 25 and 40 mL/min thresholds were set above the renal function ranges where the corresponding regimens achieved target exposure.

**Conclusions:**

Renal function category‐based VGCV dosing does not reliably achieve target GCV exposure. Current thresholds are poorly aligned with the exposure‐response relationship and warrant re‐examination. Alternative equations do not reduce the variability, supporting exposure‐guided approaches such as therapeutic drug monitoring and model‐informed precision dosing.

## Plain Language Summary

Kidney transplant recipients receive valganciclovir to prevent cytomegalovirus infection, but the dose is reduced when kidney function declines. Using computer simulations based on data from transplant patients, we found that current dosing recommendations often fail to provide the expected drug exposure. Fewer than 40% of patients reached the target level, with many receiving too little drug and others too much. Changing the formula used to estimate kidney function did not improve these results. Our findings suggest that measuring drug levels or using personalized dosing strategies could improve treatment and patient safety.

**Abbreviations**
AUC_0‐24h_
: area under the concentration–time curve from 0 to 24 h
CG: Cockcroft–Gault
CKD‐EPI: Chronic Kidney Disease Epidemiology Collaboration
CMV: cytomegalovirus
CrCl: creatinine clearance
GCV: ganciclovir
MDRD: modification of diet in renal disease
PopPK: population pharmacokinetic
VGCV: valganciclovir

## 1 Introduction

Cytomegalovirus (CMV) is one of the most frequent viral complications in solid organ transplant recipients and remains a major cause of posttransplant morbidity. While CMV infection is generally asymptomatic in immunocompetent individuals, viral replication in transplant recipients may lead to severe clinical consequences, including CMV disease, graft rejection, opportunistic infections and increased morbidity and mortality [1, 2, 3, 4].

To prevent CMV infection after transplantation, two main strategies are currently employed: preemptive therapy and universal prophylaxis. Preemptive therapy relies on regular virological monitoring and initiation of antiviral treatment once viral replication exceeds a predefined threshold. Although this strategy has proven effective, it requires intensive virological monitoring and strict adherence to follow‐up schedules, which may limit its feasibility in routine clinical practice [1, 5, 6]. Consequently, universal prophylaxis has become the preferred strategy in many centres, particularly in high‐risk patients such as CMV‐seronegative recipients of organs from CMV‐seropositive donors (D+/R−) or patients receiving potent immunosuppressive regimens [1, 7, 8].

Valganciclovir (VGCV), the oral prodrug of ganciclovir (GCV), is currently the standard agent for CMV prophylaxis in solid organ transplant recipients. Following administration, VGCV is rapidly converted to GCV, whose pharmacokinetics are largely driven by renal elimination. As a result, kidney function represents the primary determinant of systemic GCV exposure, and current dosing recommendations rely almost exclusively on dose adjustments based on renal function, typically expressed as creatinine clearance estimated using the Cockcroft–Gault equation [9, 10, 11, 12, 13]. However, in routine clinical practice, renal function is often reported as estimated glomerular filtration rate (eGFR), using MDRD or CKD‐EPI equations, raising uncertainty regarding the impact of the renal function equation used on dosing decisions and drug exposure.

Moreover, increasing evidence suggests that renal function‐based dosing alone results in highly variable and frequently suboptimal GCV exposure. Sever with similar levels of renal function, particularly in those with moderate to severe renal impairment, thereby increasing the risk of inadequate exposure [10, 14, 15, 16]. Although inadequate ganciclovir exposure has been suggested as a potential contributor to insufficient viral suppression and CMV breakthrough, the exposure–response relationship during prophylaxis remains incompletely characterized. A prophylactic target AUC_0‐24h_ range of approximately 40–60 mg.h/L has been proposed in pharmacokinetic/pharmacodynamic studies and is commonly used as a reference range in pharmacokinetic investigations and therapeutic drug monitoring of ganciclovir prophylaxis [1, 17]. Notably, the Fourth International Consensus Guidelines on the Management of Cytomegalovirus in Solid Organ Transplantation, published in 2025 [1], increasingly recommend considering TDM for ganciclovir/valganciclovir in patients at higher risk of suboptimal exposure, including those with unstable or extreme renal function.

Despite these observations, VGCV dosing in routine clinical practice remains largely empirical and is rarely guided by therapeutic drug monitoring. A better characterization of exposure variability under current renal function‐based dosing recommendations is therefore needed to clarify the extent to which these strategies achieve target exposure and to identify situations in which empirical dose adjustment may lead to under‐ or overexposure. Such characterization may also help better define the pharmacokinetic rationale for exposure‐guided individualization, including therapeutic drug monitoring, model‐informed precision dosing (MIPD) and machine learning (ML)–based approaches [18, 19, 20].

In this context, the present study aimed to evaluate the ability of current renal function‐based dosing recommendations for VGCV to achieve target GCV exposure in solid organ transplant recipients. Using population pharmacokinetic simulations, we assessed exposure distributions and target attainment under dosing recommendations based on Cockcroft–Gault‐estimated creatinine clearance. We then evaluated how alternative renal function equations commonly used in clinical practice (MDRD and CKD‐EPI equations) influence patient classification across dosing categories and subsequent exposure patterns.

## 2 Material and Methods

As this study was based exclusively on pharmacokinetic simulations and did not involve patient data or biological samples, ethical approval and informed consent were not required. The code used to perform the simulations and analyses is provided in the Supporting Information for full transparency and reproducibility. The study was conducted in accordance with the Basic and Clinical Pharmacology and Toxicology policy for experimental and clinical studies [21].

### 2.1 Pharmacokinetic Model Selection

A literature search was conducted in PubMed to identify published population pharmacokinetic models of ganciclovir in transplant recipients. The search was performed using the terms ‘population pharmacokinetic model,’ ‘ganciclovir,’ ‘adult,’ and ‘solid organ transplant.’ The population pharmacokinetic (POPPK) model selection process and predefined selection criteria are summarized in Figure S1. Among the identified models, the model developed by Lalagkas et al. [10] was selected for the simulations as it was the only model that strictly fulfilled all predefined selection criteria and allowed for complete implementation in the R environment. Because the objective was to explore the structural consequences of current dosing categories rather than to develop a new pharmacokinetic model, this model was used as the reference simulation framework.

The model was developed from 60 solid organ transplant recipients (45 kidney, 10 liver and 5 heart transplant recipients; median age 57 years [range 22–78]; median body weight 68 kg [range 43–131]; median body surface area 1.76 m^2^ [range 1.31–2.64], with a median CKD‐EPI‐estimated renal function of 55 mL/min/1.73 m^2^ at baseline). It was internally validated by nonparametric bootstrap (1000 replicates), prediction‐corrected visual predictive checks and normalized prediction distribution error analyses, and was further validated externally in an independent cohort of 22 solid organ transplant recipients from a separate centre, using bias (median prediction error) and imprecision (root mean squared prediction error) metrics on peak and trough concentrations; the CKD‐EPI‐based model showed the lowest prediction bias among the three renal function descriptors evaluated; for instance, imprecision of predicted trough concentrations (geometric mean 1.49 μg/mL in the external cohort) was approximately 19%.

The final model described ganciclovir clearance as CL = 6.93 × (CKD‐EPI/55)^0.817 × (BW/70)^0.75 L/h, with interindividual variability retained on CL, Vc, Vp, Ka and F, and between‐occasion variability on CL (*ω* ^2^ = 0.025). Renal function, estimated by the CKD‐EPI equation, and body weight entered the model as two separate, independently estimated covariates on clearance. Consistent with this structure, and with the original publication, CKD‐EPI‐ and MDRD‐derived renal function values are expressed as mL/min/1.73 m^2^ throughout the present study, without conversion to absolute renal clearance values: Doing so would reintroduce the influence of body size a second time, duplicating an effect already captured independently through the body weight covariate. Cockcroft–Gault values, in contrast, are expressed in absolute mL/min, as body weight is already incorporated directly into this formula.

The published model structure and parameter estimates were implemented in R (Version 4.3.2) using the *mrgsolve* package. Monte Carlo simulations were performed to generate pharmacokinetic profiles under predefined dosing scenarios. Model implementation was verified by simulating steady‐state ganciclovir exposure profiles and comparing the resulting AUC_0‐24h_ values with those reported in the original publication. The main structural characteristics and parameter estimates are presented in Table S1.

For the simulations, interindividual variability was retained as reported in the original model, whereas residual unexplained variability was fixed at a minimal value (*σ* = 0.001) to prevent additional stochastic noise and to isolate the influence of interindividual variability and covariates on systemic exposure.

### 2.2 Simulation Framework and Virtual Population

Steady‐state ganciclovir pharmacokinetic profiles following repeated oral administration of valganciclovir were simulated using Monte Carlo methods in a virtual cohort of adult solid organ transplant recipients (*n* = 10 000). For each simulated individual, age and serum creatinine were generated from truncated normal distributions parameterized to match the means, standard deviations and ranges reported for the model development cohort by Lalagkas et al. [10] Body weight and height were jointly generated from truncated normal marginal distributions, using a Gaussian copula to impose a Pearson correlation coefficient of 0.55 between the two variables, consistent with previously reported anthropometric correlations [22, 23]. Sex was simulated independently as a binary variable with equal probability. Renal function was subsequently estimated using the Cockcroft–Gault, MDRD and CKD‐EPI equations. MDRD and CKD‐EPI derived eGFR values were used in their indexed form (mL/min/1.73 m^2^) and were not converted to absolute values using individual body surface area (BSA). This approach was consistent with the structure of the source population pharmacokinetic model, in which CKD‐EPI estimated renal function and body weight were included as separate covariables on clearance. In contrast, Cockroft–Gault estimates were expressed in absolute mL/min because body weight is incorporated directly into the equation. No organ‐specific differences were assumed in the simulation framework.

### 2.3 Dosing Scenarios and Exposure Metrics

Valganciclovir maintenance doses were assigned according to the renal function categories defined in the Summary of Product Characteristics and the international consensus guidelines by Kotton et al. [1] (Table S2). As in the original model development by Lalagkas et al. [10], valganciclovir doses were converted to their ganciclovir‐equivalent content by multiplying the valganciclovir dose by 0.720 (ratio of the molecular weights of ganciclovir and valganciclovir); this conversion was applied to the bioavailable fraction of the depot compartment. For each equation, the distribution of patients across renal function categories was described, and the proportion of individuals reclassified into different categories when switching from Cockcroft–Gault to MDRD or CKD‐EPI was quantified. Simulations were not performed for patients with a renal function below 10 mL/min, as the population pharmacokinetic model used was not validated in this specific population.

Ganciclovir exposure was assessed from simulated concentration–time profiles by estimating individual AUC_0‐24h_ values using the linear trapezoidal rule applied to concentrations sampled every 2 h over a 168‐h steady‐state interval (AUC_0‐24h_ was derived as the mean of the cumulative AUC_0‐168h_ divided by 7, to reduce sensitivity to short‐term fluctuations within a single dosing interval). Because the trapezoidal method approximates the true, continuous concentration–time curve using discrete time points, it may slightly underestimate or overestimate the true AUC depending on the curvature of the profile between sampling points. To quantify this discretization error, AUC_0‐24h_ values obtained at the 2‐h sampling resolution used throughout the analysis were compared, for the same simulated individuals, against those derived from a high‐resolution reference simulation (0.1‐h time‐step); the median relative difference was −1.1% (mean −1.6%, SD 1.7%; *n* = 10 000), confirming that the sampling interval used had a negligible impact on the accuracy of the estimated exposure metrics. Extreme AUC values (below the 0.1st percentile or above the 99.9th percentile) were excluded from the analysis to avoid undue influence of numerical simulation artefacts on summary exposure metrics, while retaining the overall distribution of simulated exposures. Because dosing regimens ranged from once daily to once every 84 h according to renal function, cumulative AUC_0‐168h_ values were converted into mean daily AUC_0‐24h_ by dividing the former by seven, to enable standardized comparison of systemic exposure across heterogeneous dosing schedules.

Target attainment was evaluated using the prophylactic AUC_0‐24h_ range of 40–60 mg.h/L, by estimating the proportions of patients with underexposure, within the target range and with overexposure. This target range reflects the therapeutic window historically proposed for ganciclovir, in which an AUC_0‐24h_ below 40 mg.h/L has been associated with an increased risk of CMV viremia, and an AUC_0‐24h_ above 60 mg.h/L has been associated with an increased incidence of leukopenia and neutropenia [17]. This range has been widely adopted as a pragmatic reference across population pharmacokinetic studies of ganciclovir/valganciclovir prophylaxis in adult and paediatric solid organ transplant recipients [24].

### 2.4 Discontinuity Analysis Around Renal Function Dosing Thresholds

To further characterize exposure discontinuities around renal function dosing thresholds, three complementary approaches were used.

First, the continuous relationship between renal function and simulated ganciclovir exposure was examined graphically for each equation, using locally weighted scatterplot smoothing (LOESS) of individual AUC_0‐24h_ values across the full range of simulated renal function, with dosing thresholds (25, 40 and 60 mL/min or mL/min/1.73 m^2^, as applicable) and the AUC_0‐24h_ target range (40–60 mg.h/L) superimposed. This approach allowed visual inspection of exposure discontinuities across the entire renal function scale, rather than at isolated cutoff points.

Second, each of the four SmPC‐defined dosing regimens was extended across the full renal function range, independently of the assigned dosing category, to test directly whether the current thresholds align with the exposure‐response relationship. This allowed identification, for each regimen, of the renal function range over which simulated exposure fell within the AUC_0‐24h_ target range (40–60 mg.h/L) (Table S3).

Third, exposure discontinuities were quantified locally around each dosing threshold by comparing exposure metrics between two narrow, adjacent renal function intervals: one immediately below and one immediately above each threshold, each 1 mL/min wide and together forming a 2 mL/min window centred on the threshold (25, 40 and 60 mL/min). For each threshold, the median AUC_0‐24h_ and the proportion of patients achieving the target exposure were compared between the two adjacent intervals. A discontinuity index (DI), defined as the ratio of Δ target attainment to Δ median AUC, was calculated for each dosing threshold. Δ target attainment was defined as the difference in target attainment (percentage points) between the interval above and the interval below the threshold. Δ median AUC (%) was defined as the relative difference in median AUC between the interval above and the interval below the threshold, expressed relative to the value in the interval below the threshold. Because these narrow intervals contain a limited number of simulated patients, the precision of the DI was assessed by nonparametric bootstrap (1000 replicates, resampling patients with replacement within each equation), from which 95% confidence intervals were derived for each threshold and equation. The DI was used as a descriptive metric to characterize how target attainment changed relative to exposure across dosing thresholds and was interpreted in conjunction with its bootstrap confidence interval and with the continuous exposure profiles described above, rather than as an isolated point estimate.

The code used to perform the simulations and analysis is provided in the Supplemental Data.

## 3 Results

### 3.1 Simulated Population and Renal Function Distribution

A total of 10 000 virtual adult solid organ transplant recipients were simulated. After exclusion of outliers (*n* = 200), 9800 patients were retained for the final analysis. For everyone, renal function was estimated using the Cockcroft–Gault, CKD‐EPI and MDRD equations. The final simulated cohort (*n* = 9800) had a median age of 54 years (range 22–78), a median body weight of 71.1 kg (range 43–130), a median height of 165 cm (range 145–191), a SCR median of 140 μmol/L (range 40–600) and comprised 50.7% male and 49.3% female patients.

Median renal function was 46 mL/min (range 10–294) (Cockcroft–Gault), 37 mL/min/1.73 m^2^ (range 9–221) (MDRD) and 39 mL/min/1.73 m^2^ (range 10–155) (CKD‐EPI). Renal function estimates covered a wide range of values, allowing representation of all valganciclovir dosing categories defined in the Summary of Product Characteristics.

The distribution of renal function categories according to Cockcroft–Gault was used as the reference classification: creatinine clearance was ≥ 60 mL/min in 3131 patients (32.0%), 40–59 mL/min in 2778 patients (28.3%), 25–39 mL/min in 2926 patients (29.9%) and 10–24 mL/min in 965 patients (9.8%). Comparable overall distributions were observed with CKD‐EPI and MDRD.

### 3.2 Reclassification Across Renal Function Equations

Compared with Cockcroft–Gault, MDRD and CKD‐EPI reclassified a substantial proportion of patients (45.1% and 39.9%, respectively), predominantly toward lower renal function categories (Table 1). This downward shift was most pronounced in intermediate ranges: 59.5% (MDRD) and 50.6% (CKD‐EPI) of patients in the 40–59 mL/min category and 40.2% (MDRD) and 33.9% (CKD‐EPI) of patients in the 25–39 mL/min category, were downgraded, while upward reclassification remained minimal overall (4.7% for MDRD, 6.2% for CKD‐EPI). In contrast, classification was more stable at the extremes of renal function: 63.6% (MDRD) and 71.2% (CKD‐EPI) of patients with Cockcroft–Gault ≥ 60 mL/min and 85.8% (MDRD) and 84.1% (CKD‐EPI) of patients with Cockcroft–Gault 10–24 mL/min, retained their initial classification. Overall, these results demonstrate a consistent downward shift in renal function classification when using MDRD or CKD‐EPI, particularly in intermediate renal function ranges.

**TABLE 1:** Reclassification matrices comparing Cockcroft–Gault with MDRD and CKD‐EPI equations.

Cockcroft–Gault (mL/min)	≥ 60	40–59	25–39	10–24	Total
**MDRD (mL/min/1.73 m^2^)**					
≥ 60	**1992 (63.6%)**	129 (4.6%)	0 (0.0%)	0 (0.0%)	2121
40–59	973 (31.1%)	**996 (35.9%)**	190 (6.5%)	1 (0.1%)	2160
25–39	165 (5.3%)	1512 (54.4%)	**1561 (53.3%)**	136 (14.1%)	3374
10–24	1 (0.0%)	141 (5.1%)	1175 (40.2%)	**827 (85.8%)**	2144
**Total**	3131 (100.0%)	2778 (100.0%)	2926 (100.0%)	964 (100.0%)	**9799** *
**CKD‐EPI (mL/min/1.73 m** ^**2**^ **)**					
≥ 60	**2229 (71.2%)**	195 (7.0%)	0 (0.0%)	0 (0.0%)	2424
40–59	801 (25.6%)	**1177 (42.4%)**	261 (8.9%)	1 (0.1%)	2240
25–39	101 (3.2%)	1329 (47.8%)	**1673 (57.2%)**	152 (15.8%)	3255
10–24	0 (0.0%)	77 (2.8%)	992 (33.9%)	**812 (84.1%)**	1881
**Total**	3131 (100.0%)	2778 (100.0%)	2926 (100.0%)	965 (100.0%)	**9800**

*Note:* Values are presented as number of patients (row percentage). Cockcroft–Gault renal function categories (expressed in mL/min) are presented in columns, while MDRD [or CKD‐EPI] categories (expressed in mL/min/1.73 m^2^, not adjusted for individual body surface area) are presented in rows. Percentages are calculated relative to the corresponding Cockcroft–Gault category. Diagonal cells indicate concordant classification (bold characters), whereas off‐diagonal cells indicate reclassification; downward reclassification corresponds to transitions toward lower renal function categories (right side of the diagonal); and upward reclassification to transitions toward higher categories (left side of the diagonal).

* One patient with a Cockcroft–Gault‐defined renal function of 10–24 mL/min was excluded from this table, as the corresponding MDRD‐estimated renal function was below 10 mL/min/1.73 m^2^ and therefore outside the four categories considered.

### 3.3 Simulated Ganciclovir Exposure Across Renal Function Strata

Despite substantial reclassification across renal function categories, the use of MDRD or CKD‐EPI equations had little impact on the overall distribution of ganciclovir exposure. Across the three equations, median AUC_0‐24h_ increased progressively with increasing renal function category, but exposure remained highly variable.

With Cockcroft–Gault‐based dosing, median AUC_0‐24h_ ranged from 23.4 mg.h/L in the 10–24 mL/min category to 46.9 mg.h/L in the ≥ 60 mL/min category. Similar exposure gradients were observed with MDRD and CKD‐EPI, with overlapping interquartile ranges across equations within each renal function stratum (Table 2).

**TABLE 2:** Median simulated ganciclovir AUC_0‐24h_ [IQR] by renal function category according to Cockcroft–Gault, MDRD and CKD‐EPI equations.

Equation	≥ 60	40–59	25–39	10–24*
**Cockcroft–** **Gault (mL/min)**	46.9 [36.6–61.2]	41.0 [32.2–51.9]	31.8 [26.2–39.3]	27.5 [23.2–33.8]
**MDRD** **(mL/min/1.73 m** ^**2**^ **)**	42.8 [33.7–54.9]	34.6 [27.4–44.7]	27.4 [22.5–33.9]	23.4 [19.4–28.6]
**CKD‐EPI** **(mL/min/1.73 m** ^**2**^ **)**	44.2 [34.6–57.2]	36.5 [28.6–47.1]	28.3 [23.0–35.3]	23.8 [19.7–29.4]

*Note:* Values are expressed as median AUC_0‐24h_ [interquartile range], mg.h/L. AUC_0‐24h_ corresponds to the mean daily exposure derived from cumulative AUC_0‐168h_. Substantial overlap of interquartile ranges across equations indicates similar exposure distributions.

* Dosing every 84 h corresponds to twice‐weekly administration (e.g., Monday and Thursday or Monday and Friday), rather than a fixed daily schedule.

### 3.4 Target Attainment

Using standard dosing recommendations, only a minority of patients achieved the target AUC_0‐24h_ range of 40–60 mg.h/L, regardless of the renal function equation used (Table 3 and Figure 1).

**TABLE 3:** Target attainment (%) by renal function category according to Cockcroft–Gault, MDRD and CKD‐EPI equations.

Equation	Renal function category (mL/min)	Underexposure (%)	Target attainment (%)	Overexposure (%)
**Cockcroft–** **Gault**	≥ 60	33.7	**39.6**	26.8
	40–59	47.3	**39.5**	13.2
	25–39	76.4	**20.0**	3.6
	10–24	89.4	**9.1**	1.5
**MDRD**	≥ 60	42.9	**39.8**	17.3
	40–59	64.5	**29.1**	6.4
	25–39	87.3	**11.2**	1.5
	10–24	94.1	**5.2**	0.7
**CKD‐EPI**	≥ 60	39.8	**39.5**	20.7
	40–59	59.4	**32.0**	8.6
	25–39	85.0	**13.1**	1.9
	10–24	93.3	**5.9**	0.8

*Note:* Underexposure: AUC_0‐24h_ < 40 mg.h/L; target: 40–60 mg.h/L; overexposure: > 60 mg.h/L. Values are expressed as %.

**FIGURE 1:**
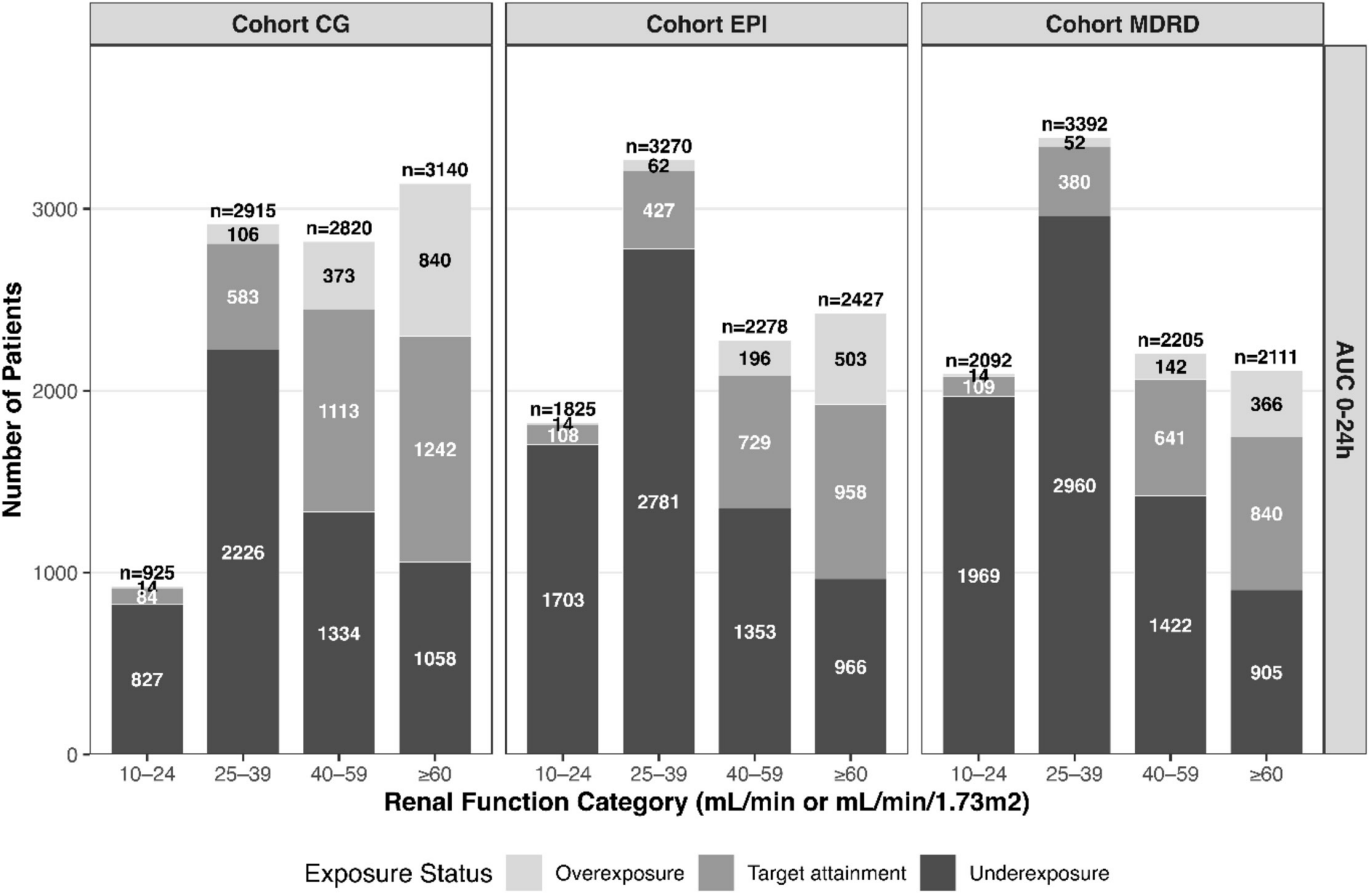
Distribution of ganciclovir exposure categories according to renal function category and estimation equation. Stacked bars represent the number of patients classified as underexposed (AUC_0‐24h_ < 40 mg.h/L), within target exposure (40–60 mg.h/L) or overexposed (> 60 mg.h/L), across renal function categories estimated using Cockcroft–Gault (CG), CKD‐EPI and MDRD equations. Total numbers of patients per renal function category are indicated above each bar.

With Cockcroft–Gault‐based dosing, the proportion of patients within the target range was 9.1% in the 10–24 mL/min category, 20.0% in the 40–59 mL/min category and 39.5% in the highest renal function category (≥ 60 mL/min). Underexposure predominated in lower renal function strata, affecting 89.4% of our virtual patients in the 10–24 mL/min category and 76.4% in the 25–39 mL/min category. Overexposure increased with renal function but remained less frequent, reaching 26.8% in the ≥ 60 mL/min category.

Similar patterns were observed with MDRD and CKD‐EPI. With MDRD, underexposure affected 94.1% of patients in the 10–24 mL/min category and 64.5% in the 40–59 mL/min category, whereas overexposure reached 17.3% in the ≥ 60 mL/min category. With CKD‐EPI, 93.3% of patients were underexposed in the 10–24 mL/min category and 59.4% in the 40–59 mL/min category, whereas 20.7% were overexposed in the ≥ 60 mL/min category.

Across all equations, target attainment did not exceed approximately 39.8% in any renal function category.

To further investigate the mechanisms underlying these exposure patterns, additional analyses were conducted around renal function dosing thresholds.

### 3.5 Exposure Patterns Around Renal Function Thresholds Used for Valganciclovir Dosing

Simulated ganciclovir exposure was highly heterogeneous across the renal function spectrum. Across narrow, 2 mL/min renal function intervals, a substantial proportion of patients had an exposure status (underexposure, target attainment or overexposure) outside the target window even near dosing thresholds, for all three equations (Figure 2). Detailed results are presented for Cockcroft–Gault, the current dosing standard; MDRD and CKD‐EPI results are reported alongside.

**FIGURE 2:**
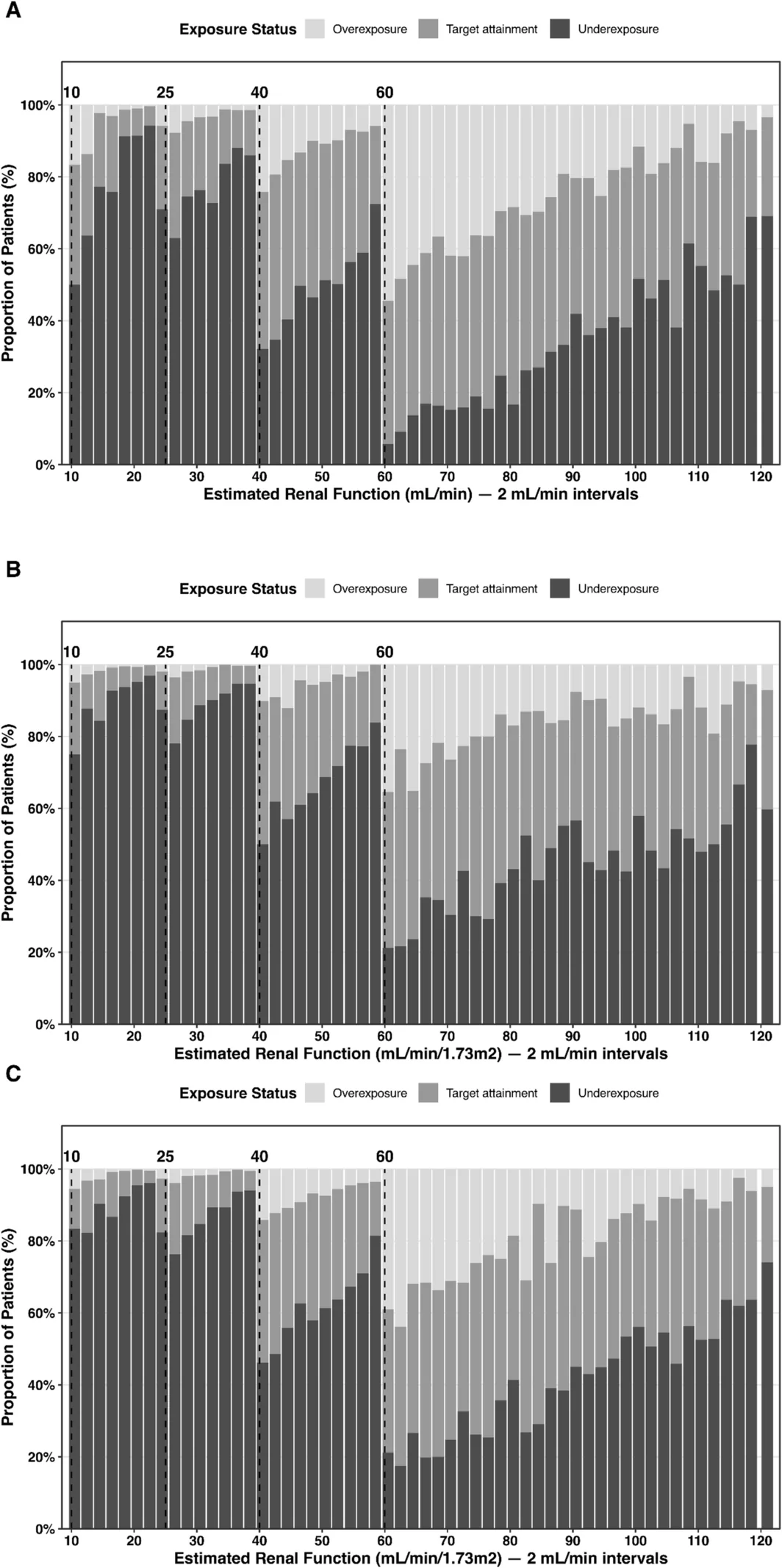
Simulated ganciclovir exposure (AUC_0‐24h_) across narrow renal function intervals according to renal function estimated using Cockcroft–Gault, MDRD or CKD‐EPI equations. (A) Cockcroft–Gault. (B) MDRD. (C) CKD‐EPI. Stacked bars represent the proportion of patients classified as underexposed (AUC_0‐24h_ < 40 mg.h/L), within target exposure (40–60 mg.h/L) or overexposed (> 60 mg.h/L) within each 2 mL/min renal function interval. Proportions were calculated within each renal function interval. Vertical dashed lines indicate valganciclovir dosing thresholds (25, 40 and 60 mL/min).

The continuous relationship between renal function and simulated exposure (Figure 3) showed a sawtooth pattern for all three equations: median AUC_0‐24h_ declined progressively as renal function increased within each dosing category, then rose abruptly at each threshold (25, 40 and 60 mL/min or mL/min/1.73 m^2^, as applicable), corresponding to the stepwise increase in assigned dose. Differences of 1 mL/min in estimated renal function were frequently associated with a change in assigned dose and an abrupt shift in exposure status.

**FIGURE 3:**
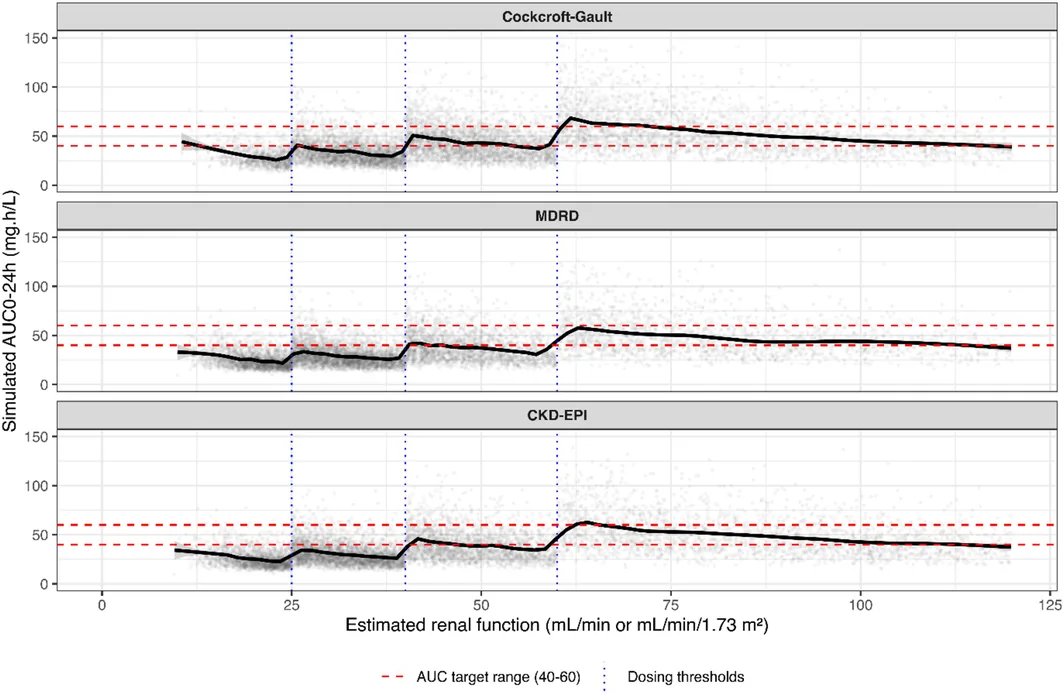
Continuous relationship between renal function and simulated ganciclovir exposure according to Cockcroft–Gault, MDRD and CKD‐EPI equations. Grey dots represent individual simulated AUC_0‐24h_ values; the black line represents the locally weighted regression (LOESS) smoothed median, with the shaded band indicating the 95% confidence interval. Blue dotted vertical lines indicate the renal function dosing thresholds (25, 40 and 60 mL/min or mL/min/1.73 m^2^, as applicable). Red dashed horizontal lines indicate the AUC_0‐24h_ target range (40–60 mg.h/L). Renal function is expressed in mL/min for Cockcroft–Gault and in mL/min/1.73 m^2^ for MDRD and CKD‐EPI.

Extending each of the four SmPC‐defined dosing regimens across the full renal function range showed that the 60 mL/min threshold was the best aligned with the exposure‐response relationship, whereas the 40 and 25 mL/min thresholds were not: With Cockcroft–Gault, the half dose achieved target between approximately 31 and 49 mL/min, below its 40 mL/min threshold and 450 mg q48h achieved target between approximately 15 and 25 mL/min, below its 25 mL/min threshold. No regimen achieved target at approximately 12–15, 25–31 and 49–67 mL/min, the 60 mL/min threshold falling within the last gap (Figure 4). MDRD and CKD‐EPI showed the same pattern, shifted toward lower values. The 10th–90th percentile bands showed that a substantial proportion of patients fell outside target even where the median was on target (Table S3).

**FIGURE 4:**
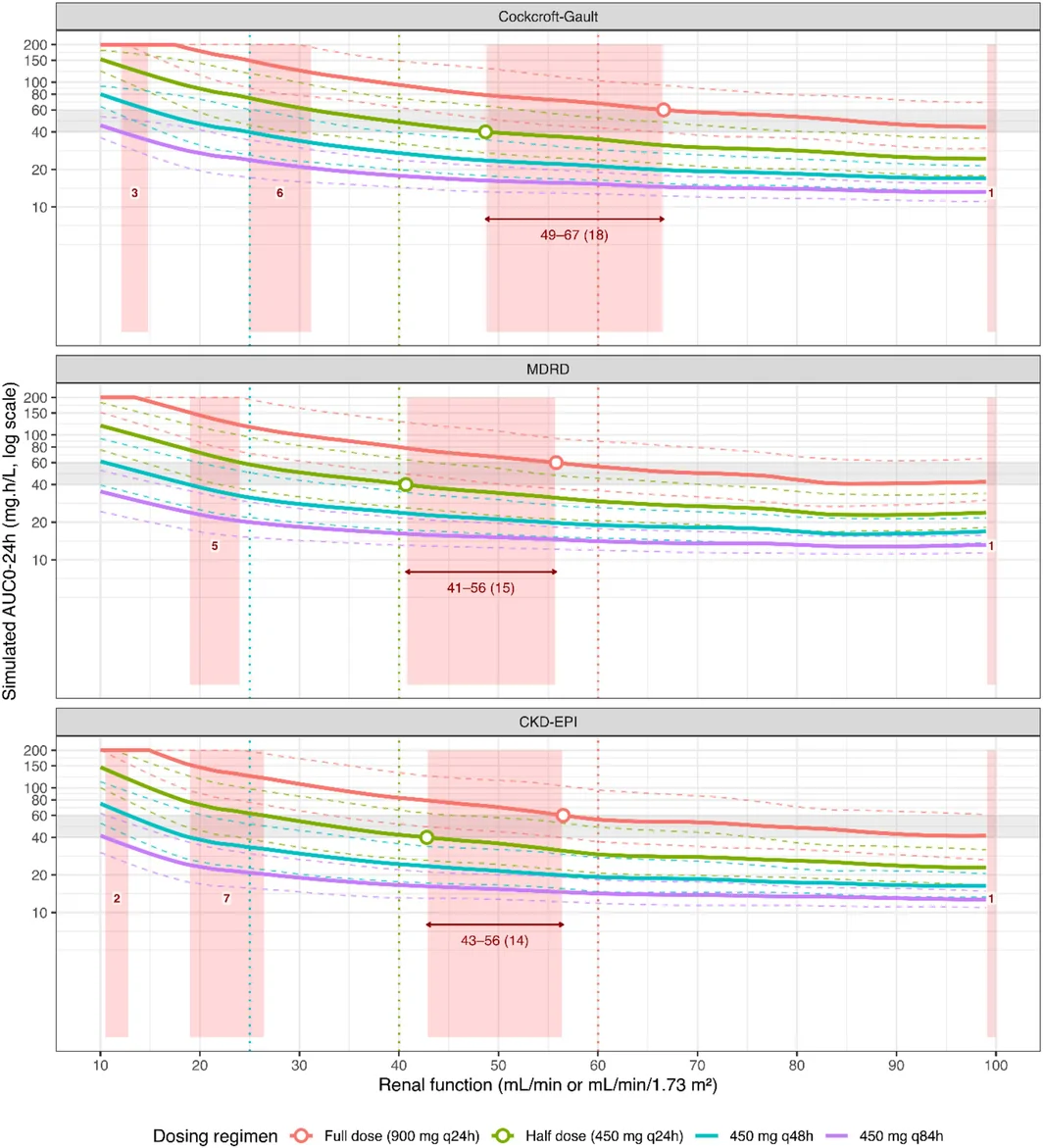
Simulated ganciclovir exposure by SmPC‐defined dosing regimen across the full renal function range. Simulated AUC_0‐24h_ (median, with 10th–90th percentiles) as a function of renal function for each of the four fixed valganciclovir dosing regimens defined in the Summary of Product Characteristics (900 mg q24h, 450 mg q24h, 450 mg q48h and 450 mg q84h), applied across the full renal function range independently of the SmPC‐assigned category. The grey band indicates the AUC_0‐24h_ target range (40–60 mg.h/L); shaded red bands indicate renal function ranges over which no regimen achieves the target range. Vertical dotted lines indicate the current valganciclovir dosing thresholds (25, 40 and 60 mL/min or mL/min/1.73 m^2^, as applicable).

The DI is reported in Table 4 together with 95% confidence intervals from a nonparametric bootstrap (1000 replicates). DI values were positive at all thresholds for all three equations, and all 95% CIs excluded zero, indicating that crossing a dosing threshold consistently increased target attainment. DI values were similar at the 25 and 40 mL/min thresholds (range 0.39–0.55), with target attainment increasing by 19 to 35 percentage points across these thresholds. They were lower and less precise at the 60 mL/min threshold (0.20–0.30), most markedly for Cockcroft–Gault (DI = 0.20, 95% CI: 0.06 to 0.40), where the dose doubling shifted part of the patients from target attainment into overexposure. Confidence intervals largely overlapped across equations.

**TABLE 4:** Quantification of exposure discontinuities across renal function dosing thresholds. Exposure metrics were compared between adjacent renal function intervals centred on each valganciclovir dosing threshold (see Methods).

Threshold	Interval below	Interval above	Δ target attainment (%)	Median Δ AUC (%)	DI (SD) [95% CI]
**Cockcroft–** ***Gault equation***					
25	24–25	25–26	33.2	60.9	0.55 (0.06) [0.43; 0.65]
40	39–40	40–41	34.8	66.6	0.53 (0.08) [0.38; 0.69]
60	59–60	60–61	17.4	84.2	0.20 (0.09) [0.06; 0.40]
***MDRD equation***					
25	24–25	25–26	19.1	48.6	0.39 (0.05) [0.28; 0.5]
40	39–40	40–41	34.9	69.9	0.49 (0.05) [0.39; 0.60]
60	59–60	60–61	27.5	104.6	0.26 (0.09) [0.09; 0.47]
***CKD‐EPI equation***					
25	24–25	25–26	24.8	57.3	0.43 (0.05) [0.33; 0.53]
40	39–40	40–41	31.8	72.3	0.44 (0.07) [0.34; 0.59]
60	59–60	60–61	25.6	81.7	0.30 (0.11) [0.1; 0.56]

*Note:* Δ Target attainment is expressed in percentage points, Median Δ AUC as a percentage change and DI as the ratio between the two calculated as:

$$\mathrm{DI}=\frac{\Delta \mathrm{TA}}{\Delta \mathrm{AUC}\%}.$$

where

$$\Delta \mathrm{TA}=TA_{\text{above}}-TA_{\text{below}}$$



$$\Delta \mathrm{AUC}\%=\frac{\mathrm{AU}C_{\text{above}}-\mathrm{AU}C_{\text{below}}}{\mathrm{AU}C_{\text{below}}}\times 100.$$

A DI of 1 indicates proportional changes in target attainment relative to exposure; values > 1 indicate larger changes in target attainment relative to exposure; values between 0 and 1 indicate smaller changes in target attainment relative to exposure; and negative values indicate that target attainment and exposure change in opposite directions.

## 4 Discussion

### 4.1 Main Findings and Impact of Renal Function Equations

This simulation study highlights structural limitations of categorical renal function–based valganciclovir dosing. Although renal function equations substantially modified patient classification across dosing categories, they had limited influence on exposure distributions at the population level, while important variability persisted at the individual level.

Using a population pharmacokinetic model and three commonly used renal function equations, we showed that standard renal‐based dosing led to highly variable systemic exposure and poor target attainment in adult solid organ transplant recipients. Across all equations, target attainment did not exceed 39.8% in any renal function category. Underexposure was the most frequent exposure category overall and increased markedly as renal function declined.

Detailed analyses around dosing thresholds further showed that small differences in estimated renal function may produce marked changes in assigned dose, exposure and target attainment (Figure 2). These findings indicate that valganciclovir dosing strategies based solely on categorical renal function thresholds are insufficient to ensure consistent ganciclovir exposure, particularly in patients with renal function values close to dosing thresholds. To determine whether exposure variability depended on the renal function equation used for dose assignment, we applied the same valganciclovir dosing thresholds after estimating renal function with Cockcroft–Gault, MDRD or CKD‐EPI. Unlike Lalagkas et al. [10], who compared these equations as predictors of ganciclovir clearance during PopPK model development, our analysis assessed whether substituting MDRD or CKD‐EPI for Cockcroft–Gault would materially alter exposure distributions and target attainment. Because MDRD and CKD‐EPI predominantly reclassified patients toward lower renal function categories, they resulted in lower median exposure and more frequent underexposure than Cockcroft–Gault (overall target attainment 20.1% and 22.7% vs. 30.8%). However, the overall exposure pattern, with poor target attainment and abrupt changes at each threshold, was similar across equations, suggesting that variability was mainly driven by the categorical dosing framework rather than by the renal function equation itself.

### 4.2 Structural Limitations and Clinical Implications of Categorical Renal Dosing

Analyses across narrow renal function intervals showed that categorical renal dosing generates abrupt exposure discontinuities around dosing thresholds. Minimal differences in estimated renal function, sometimes within the expected variability of creatinine‐based estimates, may result in different assigned doses and substantial changes in exposure patterns.

These discontinuities were further explored using the DI, together with bootstrap confidence intervals to account for the limited number of patients within each narrow threshold interval. The continuous exposure profiles (Figure 3) illustrate this sawtooth pattern across the full renal function range for all three equations, showing progressive exposure decline within each dosing category followed by an abrupt rise at each threshold. A proportional relationship between exposure and target attainment is not expected, since target attainment is a bounded probability while AUC scales directly with dose; however, a well‐calibrated threshold would be expected to preserve similar target attainment on either side. This was not the case: target attainment increased significantly when crossing every threshold, for all three equations (ΔTA +17 to +35 percentage points; all DI 95% CIs excluding zero), indicating that patients just below each threshold were systematically disadvantaged by the dose reduction. This effect was most pronounced at the 25 and 40 mL/min thresholds (DI = 0.39–0.55) and smaller at 60 mL/min (DI = 0.20–0.30), where the doubling of the dose shifted some patients from target attainment into overexposure. These findings nonetheless support the view that categorical dose reductions may produce abrupt, clinically relevant changes in exposure at renal function thresholds, particularly in patients with impaired renal function, where dose reductions may shift patients toward underexposure. These findings illustrate an intrinsic limitation of categorical dosing strategies: Continuous physiological variables such as renal function are discretized into broad categories, introducing exposure variability at category boundaries. This is particularly problematic for ganciclovir, whose clearance is strongly but not exclusively determined by renal function. Interindividual variability in body weight, bioavailability and other unmeasured pharmacokinetic determinants cannot be compensated for by renal‐based dose adjustment alone [12, 13].

Current renal function‐based dosing recommendations are also constrained by the availability of fixed‐dose valganciclovir formulations (mainly 450‐mg tablets), requiring dose adjustments in relatively large increments. Consequently, the administered dose may substantially differ from the individual dose required to achieve the target exposure, particularly for patients whose optimal dose lies between two dosing categories. As a result, patients with very similar renal function but located on opposite sides of a dosing threshold may receive different doses, whereas patients with substantially different renal function values located at opposite ends of the same category may receive the same dose. This may contribute to underexposure in patients with impaired renal function, as categorical transitions may impose dose reductions that are disproportionate to small differences in estimated renal function. Conversely, in patients with preserved renal function, the standard prophylactic dose of 900 mg once daily may exceed the individual dose required in a subset of patients, with 17%–27% overexposed in the ≥ 60 category depending on the equation.

From a clinical perspective, the high frequency of simulated underexposure in patients with impaired renal function is concerning. In our simulations, underexposure predominated in the lowest renal function strata because of dose reductions recommended in the Summary of Product Characteristics. This finding is clinically relevant in light of recent international CMV consensus guidelines [1], which recommend renal function‐based valganciclovir dosing, caution against low‐dose prophylaxis and emphasize the risk of breakthrough infection and antiviral resistance associated with suboptimal exposure [2]. Similar patterns of insufficient ganciclovir exposure have been reported in clinical pharmacokinetic studies of transplant recipients [12, 13, 15].

Conversely, the high proportion of simulated overexposure observed in patients with preserved renal function raises safety concerns, as excessive ganciclovir exposure has been associated with haematological toxicity, including neutropenia and thrombocytopenia [25, 26].

Importantly, these exposure patterns persisted regardless of the renal function equation used. Although MDRD and CKD‐EPI did not materially modify exposure distributions at the population level, reclassification across renal function categories may be clinically relevant for individual patients, because it can alter the assigned valganciclovir dose and subsequent exposure. These findings highlight the limitations of empirical renal function‐based dosing strategies and provide a pharmacokinetic rationale for further evaluation of exposure‐guided dosing approaches in prospective clinical studies.

### 4.3 Toward Exposure‐Guided Dosing Strategies

Several approaches have been proposed to improve ganciclovir dosing beyond empirical renal function‐based adjustment, including therapeutic drug monitoring [12], Bayesian model‐informed precision dosing (MIPD) [19] and machine learning‐based prediction models [20]. The Fourth International Consensus Guidelines on the management of CMV in solid organ transplantation [1], published in 2025, increasingly recommend considering TDM for ganciclovir/valganciclovir in patients at higher risk of suboptimal exposure, including those with unstable or extreme renal function.

Our results provide quantitative support for these strategies by demonstrating substantial exposure variability within each renal function category under current dosing recommendations. Part of this variability reflects the unexplained between‐patient variability in clearance and bioavailability inherent to the source population pharmacokinetic model (*ω* ^2^ = 0.089 for CL and 0.028 for F, corresponding to approximately 34% combined coefficient of variation for CL/F), which cannot be resolved by refining renal function‐based dosing categories alone, regardless of the equation or cutoff values used. This is illustrated at the 60 mL/min Cockcroft–Gault threshold, where 73.3% of patients just below remained underexposed on the half dose while 57.8% of those just above became overexposed on the full dose. Across the surrounding renal function range where neither dose achieves a median exposure within target (≈49–67 mL/min), applying either dose to all patients resulted in 61.9% of patients remaining underexposed on the half dose and 67.7% becoming overexposed on the full dose, with only around 30% on target regardless of the dose used. This inherent variability constrains the maximum target attainment achievable through covariate‐based dosing and provides a pharmacokinetic rationale for exposure‐guided approaches. Bayesian model‐informed approaches, in particular, may help estimate individual exposure from sparse concentration measurements and enable individualized dose adjustment [27]. In addition, machine learning models trained on early concentration measurements and patient covariates may further improve dose individualization, especially in complex clinical situations such as the early posttransplant period or changes in renal function over time [20].

However, the clinical benefit of improving target attainment remains uncertain, given the limited evidence supporting current prophylactic ganciclovir exposure targets and the incomplete characterization of the exposure–response relationship. Consequently, future studies should not only evaluate exposure‐guided dosing strategies but also aim to validate robust PK/PD targets associated with clinically relevant outcomes. Until such data become available, exposure‐guided approaches should be viewed as promising tools to reduce pharmacokinetic variability rather than proven strategies to improve clinical efficacy or safety.

### 4.4 Strengths and Limitations

This study has several strengths. First, detailed analyses of exposure around renal function thresholds provided mechanistic insight into the limitations of categorical dosing strategies that cannot be captured by conventional group level analyses. While an exposure difference across a threshold is itself an expected consequence of the discrete dose change, our analysis further characterizes how this translates into target attainment, which was consistently lower just below each threshold (DI, Table 4) and identifies which specific thresholds are well or poorly aligned with the exposure‐response relationship (Figures 3 and 4). Second, the use of a large virtual cohort enabled comprehensive exploration of exposure patterns across the full spectrum of renal function values and dosing categories.

A methodological consideration is that valganciclovir dosing frequency varies substantially across renal function categories, ranging from once daily to once every 84 h. Systemic exposure was therefore standardized as mean daily exposure (AUC_0‐24h_) derived from cumulative weekly AUC_0‐168h_, enabling comparison across heterogeneous dosing schedules but potentially masking peak–trough fluctuations associated with extended dosing intervals.

Some limitations should be acknowledged. The simulations were based on a single published population pharmacokinetic model and may not capture the full variability observed in real‐world populations, although the selected model had undergone internal and external validation and was considered suitable for simulation. It should also be noted that this source model was itself developed using CKD‐EPI as the renal function covariate on clearance, after comparison with Cockcroft–Gault and MDRD during model building [10]. Because CKD‐EPI was structurally embedded in the clearance model used for all simulations, this may have favoured consistency between CKD‐EPI‐estimated renal function and predicted clearance, relative to Cockcroft–Gault or MDRD, regardless of the true relative accuracy of these equations in the present virtual population. Furthermore, while previous studies have compared Cockcroft–Gault, MDRD and CKD‐EPI equations for renal function estimation including in renal transplant recipients, the present work was not designed to identify a superior renal function equation, nor to formally determine optimal renal function cutoffs, for instance through change point or segmented regression methods; rather, it aimed to evaluate whether substituting MDRD or CKD‐EPI for Cockcroft–Gault in the application of current categorical valganciclovir dosing recommendations would materially alter exposure distributions and target attainment. Finally, our study evaluated three widely used renal function metrics (Cockcroft–Gault, MDRD and CKD‐EPI). Recently, the European Kidney Function Consortium (EKFC) equation has gained traction as an alternative, continuous eGFR descriptor across paediatric and adult European populations. While assessing EKFC represents a valuable perspective for future work, it is worth noting that any eGFR‐based dosing schedule inherently relies on categorizing a continuous marker into discrete clearance tiers. Consequently, while transitioning to newer equations like EKFC may refine individual clearance estimates, population‐level tiered dosing regimens fundamentally preserve threshold effects, thereby carrying a persistent risk of exposure variability and subgroup underexposure regardless of the underlying eGFR formula used. More broadly, as discussed below, the observed exposure variability across the thresholds appears primarily driven by the categorical dosing framework itself rather than by the specific renal function equation used.

Several aspects of the simulated population may also limit the generalizability of these findings. Consistent with the source model, MDRD‐ and CKD‐EPI‐derived renal function values were not adjusted for individual body surface area as a result, simulated exposures for patients with extreme body size, particularly high body surface area, may not fully reflect their true absolute renal clearance, a limitation shared with the original population pharmacokinetic model. This suggests that clinicians should exercise particular caution when applying MDRD‐ or CKD‐EPI‐based dosing categories in patients with extreme body size, where absolute renal clearance may be under‐ or overestimated and where individualized dose adjustment (e.g., through Bayesian model‐informed approaches) may be preferable to categorical dosing alone. In addition, age, sex and serum creatinine were generated independently of one another in the simulated population, except for the imposed correlation between height and weight; this may not fully capture the physiological correlations observed between these covariates in real‐world populations (for instance, between age, sex and creatinine generation via muscle mass), which could affect the realism of the simulated distribution of renal function estimates, particularly for MDRD and CKD‐EPI equations that explicitly incorporate age and sex terms. Finally, simulated exposure was interpreted using a literature‐derived AUC_0‐24h_ target range, selected because it is widely used as a pragmatic reference in pharmacokinetic studies of ganciclovir prophylaxis. However, this target has not been prospectively validated against clinical outcomes in transplant recipients. Prospective studies are therefore required both to validate clinically relevant PK/PD targets and to determine whether improving target attainment translates into improved virological outcomes or reduced toxicity.

As anticipated above, this consistency across three renal function equations and multiple analytical approaches confirms that the categorical dosing framework itself, rather than the choice of renal function equation, remains the principal driver of exposure variability across the thresholds. Extending each SmPC‐defined dosing regimen across the full renal function range (Figure 4) further indicates that the current thresholds and dose steps themselves warrant re‐examination, particularly around the 40 and 25 mL/min thresholds. Overall, current valganciclovir dosing strategies based on renal function categories lead to highly variable ganciclovir exposure. These findings highlight the limitations of categorical renal function‐based dosing and support further evaluation of exposure‐guided approaches, including therapeutic drug monitoring as a complement to renal function‐based dosing and model‐informed precision dosing, consistent with recent international guidelines [1], while acknowledging that their clinical benefit remains to be demonstrated.

## Conflicts of Interest

F.M. and H.S. declare no conflicts of interest. C.M. has received speaker honoraria and/or research funding from Chiesi, Astellas, Pfizer and Gilead. P.M. has received speaker and consulting honoraria and/or research grants from MedinCell, Chiesi, Sandoz, Astellas and Bristol Myers Squibb (BMS). J.‐B.W. has received speaker honoraria from Chiesi, Astellas, Pfizer, Vifor and Gilead. S.B. has received lecture fees from Astellas and Chiesi. M.L. has received speaker honoraria from Chiesi.

## Supporting information




**Data S1:** Supporting Information.

**Table S1:** Main characteristics of the population pharmacokinetic model of ganciclovir reported by Lalagkas et al.^10^ and used for simulations.^a^
**Table S2:** Valganciclovir prophylactic dosing categories according to renal function (European Summary of Product Characteristics).
Table 3 Renal function ranges achieving target AUC_0‐24h_ (40–60 mg.h/L) under fixed valganciclovir dosing regimens, and gap zones with no adequate regimen, by renal function equation.

**Figure S1:** Literature review and selection process of population pharmacokinetic models of ganciclovir Flowchart summarizing the PubMed‐based identification and selection of published population pharmacokinetic (popPK) models of ganciclovir in adult solid organ transplant recipients according to predefined criteria.
**Figure S2:** Abrupt changes in simulated ganciclovir exposure around renal function dosing thresholds. Zoomed analyses around valganciclovir dosing thresholds (25, 40 and 60 mL/min), showing the proportion of patients classified as underexposed (AUC0‐24h < 40 mg.h/L), within target exposure (40–60 mg.h/L) or overexposed (> 60 mg.h/L) in narrow renal function intervals surrounding each threshold (±4 mL/min).

**Data S2:** Supporting Information.

## Data Availability

The data that support the findings of this study are available from the corresponding author upon reasonable request.
