# Supplementary material for: Renal Function–Guided Valganciclovir Dosing and Ganciclovir Exposure Variability: A Population Pharmacokinetic Simulation Study
Source: Basic Clin Pharmacol Toxicol. 2026 Nov 5;139(6):e70329. doi: 10.1111/bcpt.70329 (PMC13740662; doi:10.1111/bcpt.70329)
Supplement: Supplementary file 1 [file BCPT-139-0-s001.docx]

**Supplemental Table 1. Main characteristics of the population pharmacokinetic model of ganciclovir reported by Lalagkas et al. ^10^ and used for simulations^a^**

| Structural model | Two-compartment model with first-order absorption and elimination | | |
| --- | --- | --- | --- |
| Software | NONMEM 7.4 (original model) | | |
| Pharmacokinetic parameters | CL  Vc  Q  Vp  Lag time  Ka  F | | 6.93 L/h  43.1 L  9.23 L/h  219 L  0.331 h (fixed)  0.766 h^-1^ (fixed)  69.9% |
| Between-subject variability (variance) | ω^2^(CL)  ω^2^(Vc)  ω^2^(Vp)  ω^2^(Ka)  ω^2^(F) | | 0.0893  0.130  1.07  0.209  0.0275 |
| Between-occasion variability | φ2(CL) | | 0.025 |
| Residual error (original model) | Proportional error σ  Additive error σ | | 0.079524  0.237 |
| Covariate model | CL  Vc  Vp  Q | on CKD-EPI, BW  on BW  on BW  on BW | |

^a^Abbreviations: CL: Central clearance; Vc: Central volume; Q: Intercompartmental clearance; Vp: Peripheral volume; F: Bioavailability; Ka: Absorption rate; σ: Residual error; ω^2^: Variance of between-subject variability; φ^2^: Between occasion variability; BW: Body weight; CKD-EPI: estimated glomerular filtration rate calculated using the CKD-EPI equation

Covariate relations: CL = 6.93 × (CKD-EPI/55)^0.817 × (BW/70)^0.75 L/h; Q = 9.23 × (BW/70)^0.75 L/h; Vc = 43.1 × (BW/70) L; Vp = 219 × (BW/70) L.

**Supplemental Table 2. Valganciclovir prophylactic dosing categories according to renal function (European Summary of Product Characteristics)**

| **Renal function^a^ (mL/min)** | **Dose** |
| --- | --- |
| ≥60 | 900 mg every 24 h |
| 40-59 | 450 mg every 24 h |
| 25-39 | 450 mg every 48 h |
| 10-24 | 450 mg every 84 h |
| <10 | Contraindicated |

Renal function categories correspond to the valganciclovir dosing recommendations provided in the European Summary of Product Characteristics. In the simulations, these thresholds were applied identically to creatinine clearance estimates obtained using Cockcroft-Gault, and glomerular filtration rate estimated using MDRD or CKD-EPI equations.

*Dosing every 84 hours corresponds to twice-weekly administration (e.g., Monday and Thursday, or Monday and Friday), rather than a fixed daily schedule.*

**Supplemental Table 3. Renal function ranges achieving target AUC0-24h (40–60 mg.h/L) under fixed valganciclovir dosing regimens, and gap zones with no adequate regimen, by renal function equation**

**Panel A:** Target-achieving renal function ranges, by dosing regimen and equation

| **Dosing regimen (SmPC)** | **Assigned renal function range** | **CG: range where median AUC is in target (40–60 mg.h/L)** | **MDRD: range where median AUC is in target** | **CKD-EPI: range where median AUC is in target** |
| --- | --- | --- | --- | --- |
| 900 mg q24h (full dose) | ≥60 mL/min | ≈ 67–99 mL/min | ≈ 56–99 mL/min/1.73m^2^ | ≈ 56–99 mL/min/1.73m^2^ |
| 450 mg q24h (half dose) | 40–59 mL/min | ≈ 31–49 mL/min | ≈ 24–41 mL/min/1.73m^2^ | ≈ 26–43 mL/min/1.73m^2^ |
| 450 mg q48h | 25–39 mL/min | ≈ 15–25 mL/min | ≈ 10–19 mL/min/1.73m^2^ | ≈ 13–19 mL/min/1.73m^2^ |
| 450 mg q84h | 10–24 mL/min | ≈ 10– 12 mL/min | ≈ Never in target | ≈ 10 mL/min/1.73m^2^ |

**Panel B**: Gap zones where no available regimen achieves target, by equation

| **Equation** | **Gap 1 (near 25 mL/min or mL/min/1.73m^2^ threshold)** | **Gap 2 (near 40 mL/min or mL/min/1.73m^2^ threshold)** | **Gap 3 (near 60 mL/min or mL/min/1.73m^2^ threshold)** |
| --- | --- | --- | --- |
| CG | ≈ 12–15 (3) | ≈ 25–31 (6) | ≈ 49–67 (18) |
| MDRD | ≈ NA (regimen never in target) | ≈ 19–24 (5) | ≈ 41–56 (15) |
| CKD-EPI | ≈ 10–13 (3) | ≈ 19–26 (7) | ≈ 43–56 (13) |
