# Supplementary material for: Renal Function–Guided Valganciclovir Dosing and Ganciclovir Exposure Variability: A Population Pharmacokinetic Simulation Study
Source: Basic Clin Pharmacol Toxicol. 2026 Nov 5;139(6):e70329. doi: 10.1111/bcpt.70329 (PMC13740662; doi:10.1111/bcpt.70329)
Supplement: Supplementary file 2 [file BCPT-139-0-s002.docx]

# 1 Supplementary Data

**Supplemental Figure 1. Literature review and selection process of population pharmacokinetic models of ganciclovir**

Flowchart summarizing the PubMed-based identification and selection of published population pharmacokinetic (popPK) models of ganciclovir in adult solid organ transplant recipients according to predefined criteria.

**Supplemental Figure 2. Abrupt changes in simulated ganciclovir exposure around renal function dosing thresholds.**

Zoomed analyses around valganciclovir dosing thresholds (25, 40, and 60 mL/min), showing the proportion of patients classified as underexposed (AUC_0-24h_ <40 mg.h/L), within target exposure (40-60 mg.h/L), or overexposed (>60 mg.h/L) in narrow renal function intervals surrounding each threshold (±4 mL/min).

*A: Cockcroft–Gault; B: MDRD; C: CKD-EPI.*
