# Supplementary material for: Renal Function–Guided Valganciclovir Dosing and Ganciclovir Exposure Variability: A Population Pharmacokinetic Simulation Study
Source: Basic Clin Pharmacol Toxicol. 2026 Nov 5;139(6):e70329. doi: 10.1111/bcpt.70329 (PMC13740662; doi:10.1111/bcpt.70329)
Supplement: Supplementary file 3 [file BCPT-139-0-s004.docx]

**Supplementary Data Code used for simulation**

**`Ganciclovir_Lalagkas_DFG.Rmd** **`**

---

title: "Ganciclovir_Lalagkas_DFG"

author: "Maizaud Franck"

date: "2025-08-05"

output:

word_document:

toc: true

toc_depth: '2'

pdf_document:

toc: true

toc_depth: '2'

html_document:

toc: true

toc_depth: 2

number_sections: true

df_print: paged

theme: readable

code_folding: hide

self_contained: false

toc_float: false

highlight: tango

keep_md: false

css: styles.css

warning: false

message: false

---

```{r setup, include=FALSE}

knitr::opts_chunk$set(echo = TRUE)

```

\# Loading of the library

```{r}

library(mrgsolve)

library(ggplot2)

library(tidymodels)

library(tidyverse)

library(dplyr)

library(truncnorm)

library(pracma)

library(ggalluvial)

library(tidyr)

library(conflicted)

\# priorités globales

conflict_prefer("filter", "dplyr")

conflict_prefer("lag", "dplyr")

conflict_prefer("select", "dplyr")

conflict_prefer("rename", "dplyr")

conflict_prefer("mutate", "dplyr")

conflict_prefer("summarise","dplyr")

conflict_prefer("slice", "dplyr")

```

\# Implementation of the Lalagkas (CKD-EPI formula)

Adaptation from Heather Panagiotis Nikolaos Lalagkas et al, Clinical Pharmacokinetics 2023 POPPK gcv adult IV and oral.

https://www.ncbi.nlm.nih.gov/pmc/articles/PMC10256675/

```{r}

code_Lalagkas <-

"

[SET] end=100, delta=0.1

[PARAM] @annotated

TVF	: 0.699 : Typical value of bioavaibility

ALAG : 0.331 : value of lag time (h) (fixed)

TVKA : 0.766 : Typicalvalue of absorption rate (1/h) (fixed)

TVCL : 6.93 : Typical value of Clearance (L/h)

TVVC : 43.1 : Typical value of Central volume (L)

TVVP : 219	: Typical value of Peripheral volume of distribution (L)

TVQ	: 9.23 : Typical value of Intercompartmental clearance (L/h)

SALT : 0.720 : VGCV-to-GCV conversion factor (MW GCV / MW VGCV), Lalagkas et al. 2023

CLcrCL: 0.817 : effect of Clcr on CL

WTCL : 0.75 : effect of WT on CL

WTQ	: 0.75 : effect of WT on Q

ETA1 : 0	: IVV on CL (L/h)

ETA2 : 0	: IVV on VC (L/h)

ETA3 : 0	: IVV on VP (L/h)

ETA4 : 0	: IVV on KA (L/h)

ETA5 : 0	: IVV on F (L/h)

ETA6 : 0	: IOV on CL

[PARAM] @annotated @covariates

CLcr : 55 : estimated creat clearance CDK-EPI (ml/min)

WT	: 70 : weight (kg)

[OMEGA]

0.0893

0.130

1.07

0.209

0.0275

0.025

[SIGMA]

0.079524 // proportional error 0.079524

0.237	// additive error 0.237

[CMT] @annotated

DEPOT : Depot compartment, dose entered as mg of VGCV [ADM]

CENT : Central compartment, ganciclovir [OBS] (mg/L)

PERIPH: Peripheral compartment

[MAIN]

double CL = TVCL * pow(CLcr/55, CLcrCL) * pow(WT/70, WTCL) * exp(ETA1 + ETA(1));

double VC = TVVC * (WT/70) * exp(ETA2 + ETA(2));

double VP = TVVP * (WT/70) * exp(ETA3 + ETA(3));

double Q = TVQ * pow(WT/70, WTQ);

double KA = TVKA * exp(ETA4 + ETA(4));

double F = TVF * exp(ETA5 + ETA(5));

// Doses are entered in mg of valganciclovir (VGCV);

// converted to ganciclovir (GCV)-equivalent amount as in Lalagkas et al.

ALAG_DEPOT = ALAG;

F_DEPOT	= F * SALT;

double F_GCV = F_DEPOT;

[ODE]

dxdt_DEPOT = -KA*DEPOT;

dxdt_CENT	= KA*DEPOT -(CL+Q)*CENT/VC + Q*PERIPH/VP ;

dxdt_PERIPH = Q*CENT/VC - Q*PERIPH/VP ;

[TABLE]

double DV = (CENT/VC) * (1 + EPS(1)) + EPS(2);

int i = 0;

while(DV<0 && i <100) {

simeps();

DV = (CENT/VC) *(1 + EPS(1)) + EPS(2);

++i;

}

[CAPTURE] DV CL VC VP Q F_GCV

"

model_Lalagkas <- mcode("Valganci_Lalagkas_model_SALT", code_Lalagkas)

```

✅ Improved Version:

Goal: We are now testing the Lalagkas model, which was developed using the CKD-EPI formula and includes covariates such as body weight (WT). We aim to replicate the same analysis workflow as before, in order to compare the outcomes. Specifically:

First, we will assess whether the AUC₀–₂₄h remains consistent under different dose attribution strategies.

Second, we will evaluate the proportion of patients who transition between exposure categories (e.g., underexposure, acceptable exposure, overexposure).

Finally, we will propose a new dosing strategy and determine whether it leads to improved drug exposure.

\# Testing the model

```{r}

data <- ev(ID = 1:10, amt = 900, ii=12, addl =1, ss= 1)

set.seed(050825)

data1 <- data %>%

mutate(Clcr = rtruncnorm(n(), a = 10, b = 118, mean = 54, sd = 26),

WT = rtruncnorm(n(), a = 43, b = 131, mean = 70, sd = 17))

set.seed(050825)

sim_Lalagkas <- model_Lalagkas %>%

data_set(data1) %>%

zero_re("sigma") %>%

Req(DV) %>%

mrgsim(delta = 1, end = 24)

as_tibble(sim_Lalagkas) %>%

ggplot(aes(x = time, y = DV, color = as.factor(ID))) +

geom_point(show.legend = FALSE) +

geom_line(show.legend = FALSE) +

labs(x = "Time (h)", y = "Valganciclovir concentration (mg/L)") +

theme_bw()

```

\# Creation of the database

```{r}

library(dplyr)

library(tibble)

library(truncnorm)

library(MASS)

set.seed(300725)

n <- 10000

\# -------------------------------

\# 1) AGE (inchangé)

\# -------------------------------

Age_data <- tibble(ID = 1:n) %>%

mutate(AGE = rtruncnorm(n(), a = 22, b = 78, mean = 54, sd = 12.8))

\# -------------------------------

\# 2) HEIGHT & WT corrélés (r = 0.55)

\# -------------------------------

r_hw <- 0.55

R <- matrix(c(

1, r_hw,

r_hw, 1

), nrow = 2)

Z <- MASS::mvrnorm(n = n, mu = c(0,0), Sigma = R)

U <- pnorm(Z)

Height_data <- tibble(ID = 1:n) %>%

mutate(Height = qtruncnorm(U[,1], a = 143, b = 192, mean = 164, sd = 10.13))

WT_data <- tibble(ID = 1:n) %>%

mutate(WT = qtruncnorm(U[,2], a = 43, b = 131, mean = 70, sd = 16.78))

\# -------------------------------

\# 3) SCR (inchangé)

\# -------------------------------

set.seed(30072025)

Scr_data <- tibble(ID = 1:n) %>%

mutate(SCR = rtruncnorm(n(), a = 40, b = 602, mean = 141, sd = 75.22))

\# -------------------------------

\# 4) SEX (inchangé)

\# -------------------------------

set.seed(30072025)

Gender_data <- tibble(ID = 1:n, SEX = rbinom(n, 1, 0.50))

\# -------------------------------

\# 5) Assemblage final

\# -------------------------------

data_ev_2 <- ev(ID = 1:n, ss = 1, cmt = 1, amt = 0, time = 0)

full_data <- as_tibble(data_ev_2) %>%

arrange(ID) %>%

left_join(Age_data, by = "ID") %>%

left_join(WT_data, by = "ID") %>%

left_join(Gender_data, by = "ID") %>%

left_join(Scr_data, by = "ID") %>%

left_join(Height_data, by = "ID")

\# -------------------------------

\# 6) Vérification

\# -------------------------------

cor(full_data$Height, full_data$WT)

```

\## Estimation of the DFG in function of the formule used (CG / MDRD / CKD-EPI)

```{r}

full_data <- full_data %>%

mutate(

F_SEX = ifelse(SEX == 1, 1.0, 0.85),

SCR_mgdl = SCR / 88.4,

\# Cockcroft-Gault

DFG_CG = ((140 - AGE) * WT * F_SEX) / (72 * SCR_mgdl),

\# MDRD

DFG_MDRD = 175 * (SCR_mgdl ^ -1.154) * (AGE ^ -0.203) * ifelse(SEX == 0, 0.742, 1.0),

\# CKD-EPI

kappa = ifelse(SEX == 1, 0.9, 0.7),

alpha = ifelse(SEX == 1, -0.411, -0.329),

S = ifelse(SEX == 1, 1.0, 1.018),

ratio = SCR_mgdl / kappa,

min_part = ifelse(ratio < 1, ratio ^ alpha, 1.0),

max_part = ifelse(ratio > 1, ratio ^ -1.209, 1.0),

DFG_EPI = 141 * min_part * max_part * (0.993 ^ AGE) * S

) %>%

select(-kappa, -alpha, -S,-ratio, -min_part, -max_part, -F_SEX, -SCR, -SCR_mgdl)

```

\## Attribution of the posology

\### Prophylaxie

```{r}

get_posology_prophylaxie <- function(df) {

df %>%

mutate(

amt = case_when(

CLcr>= 60 ~ 900,

CLcr >= 40 ~ 450,

CLcr >= 25 ~ 450,

CLcr >= 10 ~ 450,

TRUE ~ NA_real_

),

ii = case_when(

CLcr >= 60 ~ 24,

CLcr >= 40 ~ 24,

CLcr >= 25 ~ 48,

CLcr >= 10 ~ 84,

TRUE ~ NA_real_

),

addl = ifelse(!is.na(amt), 9, 0),

ss = ifelse(!is.na(amt), 1, 0)

)

}

```

\### Curative

```{r}

get_posology_curative <- function(df) {

df %>%

mutate(

amt = case_when(

CLcr>= 60 ~ 900,

CLcr >= 40 ~ 450,

CLcr >= 25 ~ 450,

CLcr >= 10 ~ 450,

TRUE ~ NA_real_

),

ii = case_when(

CLcr >= 60 ~ 12,

CLcr >= 40 ~ 12,

CLcr >= 25 ~ 24,

CLcr >= 10 ~ 48,

TRUE ~ NA_real_

),

addl = ifelse(!is.na(amt), 9, 0),

ss = ifelse(!is.na(amt), 1, 0)

)

}

```

\# Impact of the dose and the attribution by the CG formula

\# CKD EPI FORMULA Prophylaxie

For this model, the reference is the CKD EPI formula because the model is develop with this formula

```{r}

full_data_Lalagkas_CKD <- full_data %>%

rename(CLcr = DFG_EPI)

```

```{r}

full_data_Lalagkas_CKD_prophylaxie <- get_posology_prophylaxie(full_data_Lalagkas_CKD)

rmarkdown::paged_table(full_data_Lalagkas_CKD_prophylaxie)

```

```{r}

full_data_Lalagkas_CKD_prophylaxie_ready <- full_data_Lalagkas_CKD_prophylaxie %>%

mutate(evid = 1, cmt = 1)

```

Simulation of the patient with the doses attributes by the Cg formula (like the RCP)

```{r}

set.seed(050825)

out_Lalagkas_prophylaxie_CKD <- model_Lalagkas %>%

data_set(

full_data_Lalagkas_CKD_prophylaxie_ready %>%

select(ID, time, amt, ii, addl, ss, evid, WT, AGE, CLcr, cmt)

) %>%

mrgsim(end = 192, delta = 2)

```

```{r}

out_Lalagkas_prophylaxie_CKD_Final <- as.data.frame(out_Lalagkas_prophylaxie_CKD)

\# Joindre avec les infos patients

final_df_Lalagkas_prophylaxie_CKD_Final <- out_Lalagkas_prophylaxie_CKD_Final %>%

left_join(

full_data_Lalagkas_CKD_prophylaxie %>%

select(ID, amt, ii, addl, ss, AGE, WT, SEX, Height, CLcr, DFG_MDRD, DFG_CG),

by = "ID"

)

final_df_Lalagkas_prophylaxie_CKD_Final <- final_df_Lalagkas_prophylaxie_CKD_Final %>%

rename(DFG_EPI = CLcr)

rmarkdown::paged_table(final_df_Lalagkas_prophylaxie_CKD_Final)

```

\# Table simulation Chen

```{r}

data_long_final_Lalagkas_CKD_prophyalxie <- final_df_Lalagkas_prophylaxie_CKD_Final %>%

pivot_longer(

cols = starts_with("DFG_"),

names_to = "Formule",

names_prefix = "DFG_",

values_to = "CLcr"

)

data_long_final_Lalagkas_CKD_prophyalxie <- data_long_final_Lalagkas_CKD_prophyalxie %>%

mutate(

CLcr_class = case_when(

CLcr < 10	~ "<10",

CLcr >= 10 & CLcr < 25	~ "10–24",

CLcr >= 25 & CLcr < 40	~ "25–39",

CLcr >= 40 & CLcr < 60	~ "40–59",

CLcr >= 60	~ "≥60",

TRUE ~ NA_character_

)

)

rmarkdown::paged_table(data_long_final_Lalagkas_CKD_prophyalxie)

```

\# Individual AUC for the CG

```{r}

library(pracma)

get_indiv_auc_ranges_3 <- function(data_long, formule = "EPI") {

data_filtered <- data_long %>%

dplyr::filter(Formule == formule)

auc_per_patient <- data_filtered %>%

group_by(ID, CLcr_class) %>%

summarise(

AUC_0_24 = (trapz(time[time <= 168], DV[time <= 168]))/7,

.groups = "drop"

) %>%

pivot_longer(

cols = starts_with("AUC"),

names_to = "Intervalle",

values_to = "AUC"

)

return(auc_per_patient)

}

```

\# Summary of the AUC for the patient with the CG formula

```{r}

data_long_final_Lalagkas_CKD_prophyalxie_AUC <- get_indiv_auc_ranges_3(data_long_final_Lalagkas_CKD_prophyalxie)

#enlève les preliers et derniers outliers

quantiles <- quantile(data_long_final_Lalagkas_CKD_prophyalxie_AUC$AUC, probs = c(0.01, 0.99))

data_long_final_Lalagkas_CKD_prophyalxie_AUC <- data_long_final_Lalagkas_CKD_prophyalxie_AUC %>%

dplyr::filter(AUC > quantiles[1], AUC < quantiles [2])

rmarkdown::paged_table(data_long_final_Lalagkas_CKD_prophyalxie_AUC)

```

\# DISTRIBUTION OF THE AUC FOR THE DIFFERENT CLCR CLASS WITH THE CG FORMULA

```{r}

\# ForCED THE ORDER CLASS

data_long_final_Lalagkas_CKD_prophyalxie_AUC <- data_long_final_Lalagkas_CKD_prophyalxie_AUC %>%

mutate(CLcr_class = factor(CLcr_class, levels = c("10–24", "25–39", "40–59", "≥60")))

\# boxplot

ggplot(data_long_final_Lalagkas_CKD_prophyalxie_AUC, aes(x = CLcr_class, y = AUC)) +

geom_boxplot(fill = "gray70", outlier.shape = NA, alpha = 0.8) +

geom_jitter(width = 0.2, alpha = 0.4, color = "black", size = 0.6) +

geom_hline(yintercept = 40, linetype = "dashed", color = "red") +

geom_hline(yintercept = 60, linetype = "dashed", color = "darkgreen") +

labs(

title = "Distribution of AUC 0-24h by Creatinine Clearance Class (CKD EPI Prophylaxis)",

subtitle = "Lalagkas model",

x = "Creatinine Clearance Class (ml/min)",

y = "AUC 0-24h (mg·h/L)"

) +

theme_minimal(base_size = 11) +

theme(

plot.title = element_text(hjust = 0.5),

plot.subtitle = element_text(hjust = 0.5),

axis.text.x = element_text(angle = 45, hjust = 1)

)

```

```{r}

\# 1. Define target intervals

auc_targets <- tibble::tibble(

Intervalle = c("AUC_0_24"),

Target_min = c(40),

Target_max = c(60)

)

\# 2. Assign exposure category

auc_with_groups_Lalagkas_CKD_Prophylaxie <- data_long_final_Lalagkas_CKD_prophyalxie_AUC %>%

dplyr::filter(Intervalle %in% auc_targets$Intervalle) %>%

left_join(auc_targets, by = "Intervalle") %>%

mutate(

Exposure_group = case_when(

AUC < Target_min ~ "Underexposure",

AUC > Target_max ~ "Overexposure",

TRUE	~ "Acceptable exposure"

)

)

\# 3. Summary statistics

auc_stats_table_Lalagkas_CKD_Prophylaxie <- auc_with_groups_Lalagkas_CKD_Prophylaxie %>%

group_by(Intervalle, CLcr_class) %>%

summarise(

n = n(),

median = round(median(AUC, na.rm = TRUE), 1),

Q5 = round(quantile(AUC, 0.05, na.rm = TRUE), 1),

Q25 = round(quantile(AUC, 0.25, na.rm = TRUE), 1),

Q75 = round(quantile(AUC, 0.75, na.rm = TRUE), 1),

Q95 = round(quantile(AUC, 0.95, na.rm = TRUE), 1),

n_under	= sum(Exposure_group == "Underexposure"),

n_acceptable = sum(Exposure_group == "Acceptable exposure"),

n_over	= sum(Exposure_group == "Overexposure"),

.groups = "drop"

) %>%

mutate(

Summary	= paste0(median, " [", Q5, "–", Q95, "]"),

`Underexposure (%)` = paste0(round(100 * n_under / n, 1), "%"),

`Acceptable (%)`	= paste0(round(100 * n_acceptable / n, 1), "%"),

`Overexposure (%)`	= paste0(round(100 * n_over / n, 1), "%")

) %>%

select(Intervalle, CLcr_class, Summary,

`Underexposure (%)`, `Acceptable (%)`, `Overexposure (%)`)

\# 4. Reshape to wide format

auc_stats_table_wide_Lalagkas_CKD_Prophylaxie <- auc_stats_table_Lalagkas_CKD_Prophylaxie %>%

pivot_wider(

names_from = CLcr_class,

values_from = c(Summary, `Underexposure (%)`, `Acceptable (%)`, `Overexposure (%)`)

) %>%

arrange(match(Intervalle, c("AUC_0_24")))

\# 5. Display in RMarkdown

rmarkdown::paged_table(auc_stats_table_wide_Lalagkas_CKD_Prophylaxie)

```

\### Graph of the proportion of the patient in prophylaxie with CKD formula

```{r}

library(dplyr)

library(ggplot2)

\# 1. Define the AUC target # 40 - 60 mg.h/L for the prophylaxie scenario

auc_targets <- tibble::tibble(

Intervalle = "AUC_0_24",

Target_min = 40,

Target_max = 60

)

\# 2. Filter the AUC 0-24 by CLcr_Class

auc_classified_Lalagkas_CKD_Prophylaxie <- data_long_final_Lalagkas_CKD_prophyalxie_AUC %>%

dplyr::filter(Intervalle == "AUC_0_24") %>%

left_join(auc_targets, by = "Intervalle") %>%

mutate(

Exposure = case_when(

AUC < Target_min ~ "Underexposure",

AUC >= Target_min & AUC <= Target_max ~ "Acceptable exposure",

AUC > Target_max ~ "Overexposure"

),

CLcr_class = factor(CLcr_class, levels = c("<10", "10–24", "25–39", "40–59", "≥60")),

Exposure = factor(Exposure, levels = c("Overexposure", "Acceptable exposure", "Underexposure"))

)

\# 3. Summary of the proportion of patient

exposure_summary_Lalagkas_CKD_Prophylaxie <- auc_classified_Lalagkas_CKD_Prophylaxie %>%

group_by(CLcr_class, Exposure) %>%

summarise(n = n(), .groups = "drop") %>%

group_by(CLcr_class) %>%

mutate(prop = n / sum(n) * 100)

\# 4. Graph of the proportion of patient

ggplot(exposure_summary_Lalagkas_CKD_Prophylaxie, aes(x = CLcr_class, y = prop, fill = Exposure)) +

geom_bar(stat = "identity", position = "stack", width = 0.8) +

scale_y_continuous(labels = scales::percent_format(scale = 1)) +

scale_fill_manual(

values = c(

"Underexposure" = "#d73027",

"Acceptable exposure" = "#91bfdb",

"Overexposure" = "#fc8d59"

)

) +

labs(

title = "Exposure classification by renal clearance (AUC0-24h) CKD Formula",

subtitle = "Lalagkas model",

x = "Renal clearance class (mL/min)",

y = "% of patients",

fill = "Exposure group"

) +

theme_minimal(base_size = 10) +

theme(

legend.position = "top",

plot.title = element_text(size = 11, face = "bold"),

plot.subtitle = element_text(size = 9),

strip.text = element_text(size = 9)

)

```

\# Alluvia graph

\# AUC indiv Chen

```{r}

\# 1. Calcul de l’AUC individuelle

auc_indiv_Lalagkas <- data_long_final_Lalagkas_CKD_prophyalxie %>%

dplyr::filter(time <= 24) %>%

group_by(ID, Formule, CLcr, CLcr_class) %>%

summarise(

AUC0_24 = trapz(time, DV),

.groups = "drop"

)

\# 2. Extraction des covariables uniques par patient

covariates <- data_long_final_Lalagkas_CKD_prophyalxie %>%

select(ID, amt, AGE, SEX, WT, Height, ii) %>%

distinct()

\# 3. Fusion des deux tables

auc_indiv_Lalagkas <- auc_indiv_Lalagkas %>%

left_join(covariates, by = "ID")

\# 4. Filtrage des AUC en excluant les 1 % les plus extrêmes

quantiles <- quantile(auc_indiv_Lalagkas$AUC0_24, probs = c(0.01, 0.99), na.rm = TRUE)

auc_indiv_Lalagkas <- auc_indiv_Lalagkas %>%

dplyr::filter(AUC0_24 >= quantiles[1], AUC0_24 <= quantiles[2])

rmarkdown::paged_table(auc_indiv_Lalagkas)

```

\# Alluvia graph Lalagkas

```{r}

\# 1. Reformater en large pour capturer les transitions

df_transitions_Lalagkas <- auc_indiv_Lalagkas %>%

select(ID, Formule, CLcr_class) %>%

pivot_wider(names_from = Formule, values_from = CLcr_class)

\# 2. Compter les transitions uniques

ordre_classes <- c("≥60", "40–59", "25–39", "10–24")

df_summary_Lalagkas <- df_transitions_Lalagkas %>%

group_by(CG, MDRD, EPI) %>%

summarise(n = n(), .groups = "drop") %>%

mutate(pct = round(100 * n / sum(n), 1)) %>%

dplyr::filter(!(CG == "10–24" & MDRD == "<10" & EPI == "<10")) %>%

mutate(

CG	= factor(CG, levels = ordre_classes),

MDRD = factor(MDRD, levels = ordre_classes),

EPI = factor(EPI, levels = ordre_classes)

)

\# 3. Créer le diagramme alluvial

Alluvia_graph_Lalagkas <- ggplot(df_summary_Lalagkas,

aes(axis1 = MDRD, axis2 = CG, axis3 = EPI, y = n)) +

geom_alluvium(aes(fill = CG), width = 0.25, alpha = 0.7) +

geom_stratum(width = 0.25, fill = "grey95", color = "black") +

geom_text(

stat = "stratum",

aes(label = after_stat(stratum)),

size = 3

) +

scale_x_discrete(

limits = c("MDRD", "CG", "EPI"),

expand = c(.1, .05)

) +

scale_y_continuous(name = "Number of patients") +

scale_fill_manual(

values = c(

"10–24" = "#d73027",

"25–39" = "#fc8d59",

"40–59" = "#91bfdb",

"≥60"	= "#1a9850"

),

name = "Classe CG"

) +

theme_minimal()

\# Afficher

Alluvia_graph_Lalagkas

```

Proportion for each class of formula

```{r}

\# Répartition par classe au sein de chaque formule (sans croiser les formules)

prop_par_formule <- auc_indiv_Lalagkas %>%

count(Formule, CLcr_class, name = "n") %>%

group_by(Formule) %>%

mutate(pct = 100 * n / sum(n)) %>%

ungroup()

prop_par_formule

```

\# DF SUMMARY PROPORTION OF PATIENT WHO MOVE

```{r}

table_CG_to_MDRD <- df_summary_Lalagkas %>%

group_by(CG, MDRD) %>%

summarise(n = sum(n), .groups = "drop") %>%

group_by(CG) %>%

mutate(Proportion = round(100 * n / sum(n), 1)) %>%

arrange(CG)

rmarkdown::paged_table(table_CG_to_MDRD)

```

```{r}

table_CG_to_EPI <- df_summary_Lalagkas %>%

group_by(CG, EPI) %>%

summarise(n = sum(n), .groups = "drop") %>%

group_by(CG) %>%

mutate(Proportion = round(100 * n / sum(n), 1)) %>%

arrange(CG)

rmarkdown::paged_table(table_CG_to_EPI)

```

\## Proportion of patient who moved

```{r}

df_summary_Lalagkas <- df_summary_Lalagkas %>%

mutate(

Changed_MDRD = CG != MDRD,

Changed_EPI = CG != EPI

)

prop_change_CG_to_MDRD <- df_summary_Lalagkas %>%

group_by(CG) %>%

summarise(

n_total = sum(n),

n_changed = sum(n[CG != MDRD]),

prop_changed = round(100 * n_changed / n_total, 1)

)

rmarkdown::paged_table(prop_change_CG_to_MDRD)

prop_change_CG_to_EPI <- df_summary_Lalagkas %>%

group_by(CG) %>%

summarise(

n_total = sum(n),

n_changed = sum(n[CG != EPI]),

prop_changed = round(100 * n_changed / n_total, 1)

)

rmarkdown::paged_table(prop_change_CG_to_EPI)

total_changed_CG_to_MDRD <- sum(prop_change_CG_to_MDRD$n_changed)

total_changed_CG_to_MDRD

total_patients <- sum(prop_change_CG_to_MDRD$n_total)

prop_global_change <- round(100 * total_changed_CG_to_MDRD / total_patients, 1)

tibble::tibble(

Total_patients = total_patients,

Total_changed = total_changed_CG_to_MDRD,

Proportion_changed = paste0(prop_global_change, "%")

)

total_changed_CG_to_EPI <- sum(prop_change_CG_to_EPI$n_changed)

total_changed_CG_to_EPI

total_patients <- sum(prop_change_CG_to_EPI$n_total)

prop_global_change <- round(100 * total_changed_CG_to_EPI / total_patients, 1)

tibble::tibble(

Total_patients = total_patients,

Total_changed = total_changed_CG_to_EPI,

Proportion_changed = paste0(prop_global_change, "%")

)

```

```{r}

\# Ordre des classes (du plus haut au plus bas)

ordre_classes <- c("≥60", "40–59", "25–39", "10–24")

\# Sécuriser les facteurs

df_summary_Lalagkas <- df_summary_Lalagkas %>%

mutate(

CG	= factor(CG, levels = ordre_classes),

MDRD = factor(MDRD, levels = ordre_classes),

EPI = factor(EPI, levels = ordre_classes)

)

\# ---- Fonction utilitaire : CG -> (MDRD ou EPI) ----

compute_change_tables <- function(df, to_col = c("MDRD","EPI")) {

to_col <- match.arg(to_col)

TO <- df[[to_col]]

res <- df %>%

mutate(

CG_num = as.numeric(CG),

TO	= TO,

TO_num = as.numeric(TO),

\# CORRECTION: vers le bas si la classe cible est numériquement PLUS GRANDE

direction = dplyr::case_when(

TO_num > CG_num ~ "Vers le bas", # DFG plus bas avec la formule cible

TO_num < CG_num ~ "Vers le haut", # DFG plus haut avec la formule cible

TRUE	~ "Identique"

)

)

\# Global (dénominateur = total patients)

total_patients <- sum(res$n)

overall <- res %>%

group_by(direction) %>%

summarise(n = sum(n), .groups = "drop") %>%

mutate(pct = round(100 * n / total_patients, 1)) %>%

arrange(factor(direction, levels = c("Vers le bas","Identique","Vers le haut")))

\# Par classe CG (dénominateur = total de la classe CG)

by_CG <- res %>%

group_by(CG, direction) %>%

summarise(n = sum(n), .groups = "drop_last") %>%

group_by(CG) %>%

mutate(pct_within_CG = round(100 * n / sum(n), 1)) %>%

ungroup() %>%

arrange(CG, factor(direction, levels = c("Vers le bas","Identique","Vers le haut")))

list(overall = overall, by_CG = by_CG, long = res)

}

\# ---- CG -> MDRD ----

chg_CG_MDRD <- compute_change_tables(df_summary_Lalagkas, to_col = "MDRD")

cat("### CG → MDRD : résumé global\n")

rmarkdown::paged_table(chg_CG_MDRD$overall)

cat("### CG → MDRD : par classe CG\n")

rmarkdown::paged_table(chg_CG_MDRD$by_CG)

\# ---- CG -> EPI ----

chg_CG_EPI <- compute_change_tables(df_summary_Lalagkas, to_col = "EPI")

cat("### CG → EPI : résumé global\n")

rmarkdown::paged_table(chg_CG_EPI$overall)

cat("### CG → EPI : par classe CG\n")

rmarkdown::paged_table(chg_CG_EPI$by_CG)

\# ---- Matrices de transition (contrôle visuel) ----

\# % par classe CG (les lignes somment à 100%)

mat_CG_to_MDRD <- df_summary_Lalagkas %>%

group_by(CG, MDRD) %>% summarise(n = sum(n), .groups = "drop") %>%

group_by(CG) %>% mutate(pct_row = round(100 * n / sum(n), 1)) %>%

select(CG, MDRD, pct_row) %>%

tidyr::pivot_wider(names_from = MDRD, values_from = pct_row, values_fill = 0) %>%

arrange(CG)

mat_CG_to_EPI <- df_summary_Lalagkas %>%

group_by(CG, EPI) %>% summarise(n = sum(n), .groups = "drop") %>%

group_by(CG) %>% mutate(pct_row = round(100 * n / sum(n), 1)) %>%

select(CG, EPI, pct_row) %>%

tidyr::pivot_wider(names_from = EPI, values_from = pct_row, values_fill = 0) %>%

arrange(CG)

cat("### Matrice CG → MDRD (lignes = CG, % ligne)\n")

rmarkdown::paged_table(mat_CG_to_MDRD)

cat("### Matrice CG → EPI (lignes = CG, % ligne)\n")

rmarkdown::paged_table(mat_CG_to_EPI)

```

```{r}

\# ---- Tableau : % DFG plus haut + posologie recommandée ----

library(dplyr)

library(tidyr)

library(tibble)

\# Grille posologique par classe de CrCl (CG)

\# ⚠️ ADAPTER les posologies à ton schéma ganciclovir

dose_grid <- tibble(

CG = factor(ordre_classes, levels = ordre_classes),

dose_recommandee = c(

"Poso classe ≥60", # ex : 900 mg x2/j

"Poso classe 40–59", # ex : 450 mg x2/j

"Poso classe 25–39", # ex : 450 mg x1/j

"Poso classe 10–24"	# ex : 450 mg q48h

)

)

\# Utiliser ta fonction déjà définie pour CG -> MDRD et CG -> EPI

chg_CG_MDRD <- compute_change_tables(df_summary_Lalagkas, to_col = "MDRD")

chg_CG_EPI <- compute_change_tables(df_summary_Lalagkas, to_col = "EPI")

\# Construire le tableau : % "Vers le haut" par classe CG et par formule

tab_DFG_plus_haut <- bind_rows(

chg_CG_MDRD$by_CG %>% mutate(formule = "MDRD"),

chg_CG_EPI$by_CG %>% mutate(formule = "CKD-EPI")

) %>%

\# On ne garde que les cas où la formule estime un DFG plus élevé que CG

dplyr::filter(direction == "Vers le haut") %>%

select(CG, formule, pct_plus_haut = pct_within_CG) %>%

\# Ajouter la posologie pour chaque classe CG

left_join(dose_grid, by = "CG") %>%

\# Passer en format large : une colonne par formule

pivot_wider(

id_cols = c(CG, dose_recommandee),

names_from = formule,

values_from = pct_plus_haut,

names_prefix = "% DFG plus haut ("

) %>%

arrange(CG)

\# Affichage dans le Rmd

rmarkdown::paged_table(tab_DFG_plus_haut)

```

```{r}

\# ---- Tableau : % DFG plus bas + posologie recommandée ----

library(dplyr)

library(tidyr)

library(tibble)

\# Si dose_grid est déjà défini plus haut, tu peux sauter ce bloc

\# Grille posologique par classe de CrCl (CG)

\# ⚠️ ADAPTER les posologies à ton schéma ganciclovir

dose_grid <- tibble(

CG = factor(ordre_classes, levels = ordre_classes),

dose_recommandee = c(

"Poso classe ≥60", # ex : 900 mg x2/j

"Poso classe 40–59", # ex : 450 mg x2/j

"Poso classe 25–39", # ex : 450 mg x1/j

"Poso classe 10–24"	# ex : 450 mg q48h

)

)

\# On réutilise chg_CG_MDRD et chg_CG_EPI si déjà calculés

\# Sinon, dé-commente les 2 lignes ci-dessous :

\# chg_CG_MDRD <- compute_change_tables(df_summary_Lalagkas, to_col = "MDRD")

\# chg_CG_EPI <- compute_change_tables(df_summary_Lalagkas, to_col = "EPI")

tab_DFG_plus_bas <- bind_rows(

chg_CG_MDRD$by_CG %>% mutate(formule = "MDRD"),

chg_CG_EPI$by_CG %>% mutate(formule = "CKD-EPI")

) %>%

\# Ici on s'intéresse aux cas où la formule donne un DFG plus BAS que CG

dplyr::filter(direction == "Vers le bas") %>%

select(CG, formule, pct_plus_bas = pct_within_CG) %>%

\# Ajouter la posologie pour chaque classe CG

left_join(dose_grid, by = "CG") %>%

\# Format large : une colonne par formule

pivot_wider(

id_cols	= c(CG, dose_recommandee),

names_from = formule,

values_from = pct_plus_bas,

names_prefix = "% DFG plus bas ("

) %>%

arrange(CG)

\# Affichage dans le Rmd

rmarkdown::paged_table(tab_DFG_plus_bas)

```

```{r}

library(dplyr)

library(tidyr)

library(tibble)

#------------------------------------------------------------

\# 1) Calcul transitions CG → MDRD et CG → CKD-EPI

#------------------------------------------------------------

chg_CG_MDRD <- compute_change_tables(df_summary_Lalagkas, to_col = "MDRD")

chg_CG_EPI <- compute_change_tables(df_summary_Lalagkas, to_col = "EPI")

#------------------------------------------------------------

\# 2) n total par classe CG

#------------------------------------------------------------

n_per_CG <- df_summary_Lalagkas %>%

group_by(CG) %>%

summarise(n_CG = sum(n), .groups = "drop")

#------------------------------------------------------------

\# 3) Préparer données : high / low pour MDRD + CKD-EPI

#------------------------------------------------------------

byCG_all <- bind_rows(

chg_CG_MDRD$by_CG %>% mutate(formule = "MDRD"),

chg_CG_EPI$by_CG %>% mutate(formule = "CKD-EPI")

)

\# ---- % higher ----

tab_high <- byCG_all %>%

filter(direction == "Vers le haut") %>%

select(CG, formule, pct_high = pct_within_CG) %>%

pivot_wider(

id_cols = CG,

names_from = formule,

values_from = pct_high,

names_glue = "pct_higher_{formule}",

values_fill = 0

)

\# ---- % lower ----

tab_low <- byCG_all %>%

filter(direction == "Vers le bas") %>%

select(CG, formule, pct_low = pct_within_CG) %>%

pivot_wider(

id_cols = CG,

names_from = formule,

values_from = pct_low,

names_glue = "pct_lower_{formule}",

values_fill = 0

)

#------------------------------------------------------------

\# 4) Table de base = toutes classes CG + posologie + n

#------------------------------------------------------------

base_CG <- tibble(

CG = factor(ordre_classes, levels = ordre_classes)

) %>%

left_join(dose_grid, by = "CG") %>%

left_join(n_per_CG, by = "CG")

#------------------------------------------------------------

\# 5) Fusionner high + low + base

\#	et calculer % same category

#------------------------------------------------------------

tab_full_partial <- base_CG %>%

left_join(tab_high, by = "CG") %>%

left_join(tab_low, by = "CG") %>%

mutate(across(starts_with("pct_"), ~ tidyr::replace_na(.x, 0)))

tab_full <- tab_full_partial %>%

mutate(

pct_same_MDRD	= 100 - pct_higher_MDRD	- pct_lower_MDRD,

pct_same_CKDEPI = 100 - `pct_higher_CKD-EPI` - `pct_lower_CKD-EPI`

)

#------------------------------------------------------------

\# 6) Renommer proprement pour poster en anglais

#------------------------------------------------------------

tab_poster <- tab_full %>%

rename(

`CG category`	= CG,

`Recommended dose (CG)`	= dose_recommandee,

`n (CG class)`	= n_CG,

`% higher GFR (MDRD)`	= pct_higher_MDRD,

`% higher GFR (CKD-EPI)`	= `pct_higher_CKD-EPI`,

`% lower GFR (MDRD)`	= pct_lower_MDRD,

`% lower GFR (CKD-EPI)`	= `pct_lower_CKD-EPI`,

`% same category (MDRD)`	= pct_same_MDRD,

`% same category (CKD-EPI)` = pct_same_CKDEPI

) %>%

arrange(factor(`CG category`, levels = ordre_classes))

#------------------------------------------------------------

\# 7) Affichage

#------------------------------------------------------------

rmarkdown::paged_table(tab_poster)

```

```{r}

\# ---- 1. Récupérer n par classe CG ----

n_per_CG <- df_summary_Lalagkas %>%

group_by(CG) %>%

summarise(n_CG = sum(n), .groups = "drop")

\# ---- 2. Récupérer les % identiques pour MDRD et CKD-EPI ----

same_MDRD <- chg_CG_MDRD$by_CG %>%

filter(direction == "Identique") %>%

select(CG, pct_same_MDRD = pct_within_CG)

same_EPI <- chg_CG_EPI$by_CG %>%

filter(direction == "Identique") %>%

select(CG, pct_same_EPI = pct_within_CG)

```

\# CG

For this model, the reference is the CKD EPI formula because the model is develop with this formula

```{r}

full_data_Lalagkas_CG <- full_data %>%

rename(CLcr = DFG_CG)

```

```{r}

full_data_Lalagkas_CG_prophylaxie <- get_posology_prophylaxie(full_data_Lalagkas_CG)

full_data_Lalagkas_CG_prophylaxie <- full_data_Lalagkas_CG_prophylaxie %>%

rename(DFG_CG = CLcr,

CLcr = DFG_EPI)

rmarkdown::paged_table(full_data_Lalagkas_CG_prophylaxie)

```

```{r}

full_data_Lalagkas_CG_prophylaxie <- full_data_Lalagkas_CG_prophylaxie %>%

mutate(evid = 1, cmt = 1)

```

Simulation of the patient with the doses attributes by the CG formula (like the RCP)

```{r}

set.seed(050825)

out_Lalagkas_prophylaxie_CG <- model_Lalagkas %>%

data_set(

full_data_Lalagkas_CG_prophylaxie %>%

select(ID, time, amt, ii, addl, ss, evid, WT, AGE, CLcr, cmt)

) %>%

mrgsim(end = 192, delta = 2)

```

```{r}

out_Lalagkas_prophylaxie_CG_Final <- as.data.frame(out_Lalagkas_prophylaxie_CG)

\# Joindre avec les infos patients

final_df_Lalagkas_prophylaxie_CG_Final <- out_Lalagkas_prophylaxie_CG_Final %>%

left_join(

full_data_Lalagkas_CG_prophylaxie %>%

select(ID, amt, ii, addl, ss, AGE, WT, SEX, Height, CLcr, DFG_MDRD, DFG_CG),

by = "ID"

)

final_df_Lalagkas_prophylaxie_CG_Final <- final_df_Lalagkas_prophylaxie_CG_Final %>%

rename(DFG_EPI = CLcr)

rmarkdown::paged_table(final_df_Lalagkas_prophylaxie_CG_Final)

```

\# Table simulation Chen

```{r}

data_long_final_Lalagkas_CG_prophyalxie <- final_df_Lalagkas_prophylaxie_CG_Final %>%

pivot_longer(

cols = starts_with("DFG_"),

names_to = "Formule",

names_prefix = "DFG_",

values_to = "CLcr"

)

data_long_final_Lalagkas_CG_prophyalxie <- data_long_final_Lalagkas_CG_prophyalxie %>%

mutate(

CLcr_class = case_when(

CLcr < 10	~ "<10",

CLcr >= 10 & CLcr < 25	~ "10–24",

CLcr >= 25 & CLcr < 40	~ "25–39",

CLcr >= 40 & CLcr < 60	~ "40–59",

CLcr >= 60	~ "≥60",

TRUE ~ NA_character_

)

)

rmarkdown::paged_table(data_long_final_Lalagkas_CG_prophyalxie)

```

\# Individual AUC for the CG

```{r}

library(pracma)

get_indiv_auc_ranges_1 <- function(data_long, formule = "CG") {

data_filtered <- data_long %>%

dplyr::filter(Formule == formule)

auc_per_patient <- data_filtered %>%

group_by(ID, CLcr_class) %>%

summarise(

AUC_0_24 = (trapz(time[time <= 168], DV[time <= 168]))/7,

.groups = "drop"

) %>%

pivot_longer(

cols = starts_with("AUC"),

names_to = "Intervalle",

values_to = "AUC"

)

return(auc_per_patient)

}

```

\# Summary of the AUC for the patient with the CG formula

```{r}

data_long_final_Lalagkas_CG_prophyalxie_AUC <- get_indiv_auc_ranges_1(data_long_final_Lalagkas_CG_prophyalxie)

#enlève les preliers et derniers outliers

quantiles <- quantile(data_long_final_Lalagkas_CG_prophyalxie_AUC$AUC, probs = c(0.01, 0.99))

data_long_final_Lalagkas_CG_prophyalxie_AUC <- data_long_final_Lalagkas_CG_prophyalxie_AUC %>%

dplyr::filter(AUC > quantiles[1], AUC < quantiles [2])

rmarkdown::paged_table(data_long_final_Lalagkas_CG_prophyalxie_AUC)

```

\# DISTRIBUTION OF THE AUC FOR THE DIFFERENT CLCR CLASS WITH THE CG FORMULA

```{r}

\# ForCED THE ORDER CLASS

data_long_final_Lalagkas_CG_prophyalxie_AUC <- data_long_final_Lalagkas_CG_prophyalxie_AUC %>%

mutate(CLcr_class = factor(CLcr_class, levels = c("10–24", "25–39", "40–59", "≥60")))

\# boxplot

ggplot(data_long_final_Lalagkas_CG_prophyalxie_AUC, aes(x = CLcr_class, y = AUC)) +

geom_boxplot(fill = "gray70", outlier.shape = NA, alpha = 0.8) +

geom_jitter(width = 0.2, alpha = 0.4, color = "black", size = 0.6) +

geom_hline(yintercept = 40, linetype = "dashed", color = "red") +

geom_hline(yintercept = 60, linetype = "dashed", color = "darkgreen") +

labs(

title = "Distribution of AUC 0-24h by Creatinine Clearance Class (CG Prophylaxis)",

subtitle = "Lalagkas model",

x = "Creatinine Clearance Class (ml/min)",

y = "AUC 0-24h (mg·h/L)"

) +

theme_minimal(base_size = 11) +

theme(

plot.title = element_text(hjust = 0.5),

plot.subtitle = element_text(hjust = 0.5),

axis.text.x = element_text(angle = 45, hjust = 1)

)

```

```{r}

\# 1. Define target intervals

auc_targets <- tibble::tibble(

Intervalle = c("AUC_0_24"),

Target_min = c(40),

Target_max = c(60)

)

\# 2. Assign exposure category

auc_with_groups_Lalagkas_CG_Prophylaxie <- data_long_final_Lalagkas_CG_prophyalxie_AUC %>%

dplyr::filter(Intervalle %in% auc_targets$Intervalle) %>%

left_join(auc_targets, by = "Intervalle") %>%

mutate(

Exposure_group = case_when(

AUC < Target_min ~ "Underexposure",

AUC > Target_max ~ "Overexposure",

TRUE	~ "Acceptable exposure"

)

)

\# 3. Summary statistics

auc_stats_table_Lalagkas_CG_Prophylaxie <- auc_with_groups_Lalagkas_CG_Prophylaxie %>%

group_by(Intervalle, CLcr_class) %>%

summarise(

n = n(),

median = round(median(AUC, na.rm = TRUE), 1),

Q1 = round(quantile(AUC, 0.025, na.rm = TRUE), 1),

Q3 = round(quantile(AUC, 0.975, na.rm = TRUE), 1),

n_under	= sum(Exposure_group == "Underexposure"),

n_acceptable = sum(Exposure_group == "Acceptable exposure"),

n_over	= sum(Exposure_group == "Overexposure"),

.groups = "drop"

) %>%

mutate(

Summary	= paste0(median, " [", Q1, "–", Q3, "]"),

`Underexposure (%)` = paste0(round(100 * n_under / n, 1), "%"),

`Acceptable (%)`	= paste0(round(100 * n_acceptable / n, 1), "%"),

`Overexposure (%)`	= paste0(round(100 * n_over / n, 1), "%")

) %>%

select(Intervalle, CLcr_class, Summary,

`Underexposure (%)`, `Acceptable (%)`, `Overexposure (%)`)

\# 4. Reshape to wide format

auc_stats_table_wide_Lalagkas_CG_Prophylaxie <- auc_stats_table_Lalagkas_CG_Prophylaxie %>%

pivot_wider(

names_from = CLcr_class,

values_from = c(Summary, `Underexposure (%)`, `Acceptable (%)`, `Overexposure (%)`)

) %>%

arrange(match(Intervalle, c("AUC_0_24")))

\# 5. Display in RMarkdown

rmarkdown::paged_table(auc_stats_table_wide_Lalagkas_CG_Prophylaxie)

```

\### Graph of the proportion of the patient in prophylaxie with CG formula

```{r}

library(dplyr)

library(ggplot2)

\# 1. Define the AUC target # 40 - 60 mg.h/L for the prophylaxie scenario

auc_targets <- tibble::tibble(

Intervalle = "AUC_0_24",

Target_min = 40,

Target_max = 60

)

\# 2. Filter the AUC 0-24 by CLcr_Class

auc_classified_Lalagkas_CG_Prophylaxie <- data_long_final_Lalagkas_CG_prophyalxie_AUC %>%

dplyr::filter(Intervalle == "AUC_0_24") %>%

left_join(auc_targets, by = "Intervalle") %>%

mutate(

Exposure = case_when(

AUC < Target_min ~ "Underexposure",

AUC >= Target_min & AUC <= Target_max ~ "Acceptable exposure",

AUC > Target_max ~ "Overexposure"

),

CLcr_class = factor(CLcr_class, levels = c("<10", "10–24", "25–39", "40–59", "≥60")),

Exposure = factor(Exposure, levels = c("Overexposure", "Acceptable exposure", "Underexposure"))

)

\# 3. Summary of the proportion of patient

exposure_summary_Lalagkas_CG_Prophylaxie <- auc_classified_Lalagkas_CG_Prophylaxie %>%

group_by(CLcr_class, Exposure) %>%

summarise(n = n(), .groups = "drop") %>%

group_by(CLcr_class) %>%

mutate(prop = n / sum(n) * 100)

\# 4. Graph of the proportion of patient

ggplot(exposure_summary_Lalagkas_CG_Prophylaxie, aes(x = CLcr_class, y = prop, fill = Exposure)) +

geom_bar(stat = "identity", position = "stack", width = 0.8) +

scale_y_continuous(labels = scales::percent_format(scale = 1)) +

scale_fill_manual(

values = c(

"Underexposure" = "#1f78b4", # bleu

"Acceptable exposure" = "#33a02c", # vert

"Overexposure" = "#e31a1c"	# rouge

)

) +

labs(

title = "Exposure classification by renal clearance (AUC0-24h) CG Formula",

x = "Renal clearance class (mL/min)",

y = "% of patients",

fill = "Exposure group"

) +

theme_minimal(base_size = 10) +

theme(

legend.position = "top",

plot.title = element_text(hjust = 0.5, size = 11, face = "bold"),

strip.text = element_text(size = 9)

)

```

\# MDRD

For this model, the reference is the CKD EPI formula because the model is develop with this formula

```{r}

full_data_Lalagkas_MDRD <- full_data %>%

rename(CLcr = DFG_MDRD)

```

```{r}

full_data_Lalagkas_MDRD_prophylaxie <- get_posology_prophylaxie(full_data_Lalagkas_MDRD)

full_data_Lalagkas_MDRD_prophylaxie <- full_data_Lalagkas_MDRD_prophylaxie %>%

rename(DFG_MDRD = CLcr,

CLcr = DFG_EPI)

rmarkdown::paged_table(full_data_Lalagkas_MDRD_prophylaxie)

```

```{r}

full_data_Lalagkas_MDRD_prophylaxie <- full_data_Lalagkas_MDRD_prophylaxie %>%

mutate(evid = 1, cmt = 1)

```

Simulation of the patient with the doses attributes by the CG formula (like the RCP)

```{r}

set.seed(050825)

out_Lalagkas_prophylaxie_MDRD <- model_Lalagkas %>%

data_set(

full_data_Lalagkas_MDRD_prophylaxie %>%

select(ID, time, amt, ii, addl, ss, evid, WT, AGE, CLcr, cmt)

) %>%

mrgsim(end = 192, delta = 2)

```

```{r}

out_Lalagkas_prophylaxie_MDRD_Final <- as.data.frame(out_Lalagkas_prophylaxie_MDRD)

\# Joindre avec les infos patients

final_df_Lalagkas_prophylaxie_MDRD_Final <- out_Lalagkas_prophylaxie_MDRD_Final %>%

left_join(

full_data_Lalagkas_MDRD_prophylaxie %>%

select(ID, amt, ii, addl, ss, AGE, WT, SEX, Height, CLcr, DFG_MDRD, DFG_CG),

by = "ID"

)

final_df_Lalagkas_prophylaxie_MDRD_Final <- final_df_Lalagkas_prophylaxie_MDRD_Final %>%

rename(DFG_EPI = CLcr)

rmarkdown::paged_table(final_df_Lalagkas_prophylaxie_MDRD_Final)

```

\# Table simulation Chen

```{r}

data_long_final_Lalagkas_MDRD_prophyalxie <- final_df_Lalagkas_prophylaxie_MDRD_Final %>%

pivot_longer(

cols = starts_with("DFG_"),

names_to = "Formule",

names_prefix = "DFG_",

values_to = "CLcr"

)

data_long_final_Lalagkas_MDRD_prophyalxie <- data_long_final_Lalagkas_MDRD_prophyalxie %>%

mutate(

CLcr_class = case_when(

CLcr < 10	~ "<10",

CLcr >= 10 & CLcr < 25	~ "10–24",

CLcr >= 25 & CLcr < 40	~ "25–39",

CLcr >= 40 & CLcr < 60	~ "40–59",

CLcr >= 60	~ "≥60",

TRUE ~ NA_character_

)

)

rmarkdown::paged_table(data_long_final_Lalagkas_MDRD_prophyalxie)

```

\# Individual AUC for the CG

```{r}

library(pracma)

get_indiv_auc_ranges_2 <- function(data_long, formule = "MDRD") {

data_filtered <- data_long %>%

dplyr::filter(Formule == formule)

auc_per_patient <- data_filtered %>%

group_by(ID, CLcr_class) %>%

summarise(

AUC_0_24 = (trapz(time[time <= 168], DV[time <= 168]))/7,

.groups = "drop"

) %>%

pivot_longer(

cols = starts_with("AUC"),

names_to = "Intervalle",

values_to = "AUC"

)

return(auc_per_patient)

}

```

\# Summary of the AUC for the patient with the CG formula

```{r}

data_long_final_Lalagkas_MDRD_prophyalxie_AUC <- get_indiv_auc_ranges_2(data_long_final_Lalagkas_MDRD_prophyalxie)

#enlève les preliers et derniers outliers

quantiles <- quantile(data_long_final_Lalagkas_MDRD_prophyalxie_AUC$AUC, probs = c(0.01, 0.99))

data_long_final_Lalagkas_MDRD_prophyalxie_AUC <- data_long_final_Lalagkas_MDRD_prophyalxie_AUC %>%

dplyr::filter(AUC > quantiles[1], AUC < quantiles [2])

rmarkdown::paged_table(data_long_final_Lalagkas_MDRD_prophyalxie_AUC)

```

\# DISTRIBUTION OF THE AUC FOR THE DIFFERENT CLCR CLASS WITH THE MDRD FORMULA

```{r}

\# ForCED THE ORDER CLASS

data_long_final_Lalagkas_MDRD_prophyalxie_AUC <- data_long_final_Lalagkas_MDRD_prophyalxie_AUC %>%

mutate(CLcr_class = factor(CLcr_class, levels = c("10–24", "25–39", "40–59", "≥60")))

\# boxplot

ggplot(data_long_final_Lalagkas_MDRD_prophyalxie_AUC, aes(x = CLcr_class, y = AUC)) +

geom_boxplot(fill = "gray70", outlier.shape = NA, alpha = 0.8) +

geom_jitter(width = 0.2, alpha = 0.4, color = "black", size = 0.6) +

geom_hline(yintercept = 40, linetype = "dashed", color = "red") +

geom_hline(yintercept = 60, linetype = "dashed", color = "darkgreen") +

labs(

title = "Distribution of AUC 0-24h by Creatinine Clearance Class (MDRD Prophylaxis)",

subtitle = "Lalagkas model",

x = "Creatinine Clearance Class (ml/min)",

y = "AUC 0-24h (mg·h/L)"

) +

theme_minimal(base_size = 11) +

theme(

plot.title = element_text(hjust = 0.5),

plot.subtitle = element_text(hjust = 0.5),

axis.text.x = element_text(angle = 45, hjust = 1)

)

```

```{r}

\# 1. Define target intervals

auc_targets <- tibble::tibble(

Intervalle = c("AUC_0_24"),

Target_min = c(40),

Target_max = c(60)

)

\# 2. Assign exposure category

auc_with_groups_Lalagkas_MDRD_Prophylaxie <- data_long_final_Lalagkas_MDRD_prophyalxie_AUC %>%

dplyr::filter(Intervalle %in% auc_targets$Intervalle) %>%

left_join(auc_targets, by = "Intervalle") %>%

mutate(

Exposure_group = case_when(

AUC < Target_min ~ "Underexposure",

AUC > Target_max ~ "Overexposure",

TRUE	~ "Acceptable exposure"

)

)

\# 3. Summary statistics

auc_stats_table_Lalagkas_MDRD_Prophylaxie <- auc_with_groups_Lalagkas_MDRD_Prophylaxie %>%

group_by(Intervalle, CLcr_class) %>%

summarise(

n = n(),

median = round(median(AUC, na.rm = TRUE), 1),

Q1 = round(quantile(AUC, 0.025, na.rm = TRUE), 1),

Q3 = round(quantile(AUC, 0.975, na.rm = TRUE), 1),

n_under	= sum(Exposure_group == "Underexposure"),

n_acceptable = sum(Exposure_group == "Acceptable exposure"),

n_over	= sum(Exposure_group == "Overexposure"),

.groups = "drop"

) %>%

mutate(

Summary	= paste0(median, " [", Q1, "–", Q3, "]"),

`Underexposure (%)` = paste0(round(100 * n_under / n, 1), "%"),

`Acceptable (%)`	= paste0(round(100 * n_acceptable / n, 1), "%"),

`Overexposure (%)`	= paste0(round(100 * n_over / n, 1), "%")

) %>%

select(Intervalle, CLcr_class, Summary,

`Underexposure (%)`, `Acceptable (%)`, `Overexposure (%)`)

\# 4. Reshape to wide format

auc_stats_table_wide_Lalagkas_MDRD_Prophylaxie <- auc_stats_table_Lalagkas_MDRD_Prophylaxie %>%

pivot_wider(

names_from = CLcr_class,

values_from = c(Summary, `Underexposure (%)`, `Acceptable (%)`, `Overexposure (%)`)

) %>%

arrange(match(Intervalle, c("AUC_0_24")))

\# 5. Display in RMarkdown

rmarkdown::paged_table(auc_stats_table_wide_Lalagkas_MDRD_Prophylaxie)

```

\### Graph of the proportion of the patient in prophylaxie with CKD formula

```{r}

library(dplyr)

library(ggplot2)

\# 1. Define the AUC target # 40 - 60 mg.h/L for the prophylaxie scenario

auc_targets <- tibble::tibble(

Intervalle = "AUC_0_24",

Target_min = 40,

Target_max = 60

)

\# 2. Filter the AUC 0-24 by CLcr_Class

auc_classified_Lalagkas_MDRD_Prophylaxie <- data_long_final_Lalagkas_MDRD_prophyalxie_AUC %>%

dplyr::filter(Intervalle == "AUC_0_24") %>%

left_join(auc_targets, by = "Intervalle") %>%

mutate(

Exposure = case_when(

AUC < Target_min ~ "Underexposure",

AUC >= Target_min & AUC <= Target_max ~ "Acceptable exposure",

AUC > Target_max ~ "Overexposure"

),

CLcr_class = factor(CLcr_class, levels = c("<10", "10–24", "25–39", "40–59", "≥60")),

Exposure = factor(Exposure, levels = c("Overexposure", "Acceptable exposure", "Underexposure"))

)

\# 3. Summary of the proportion of patient

exposure_summary_Lalagkas_MDRD_Prophylaxie <- auc_classified_Lalagkas_MDRD_Prophylaxie %>%

group_by(CLcr_class, Exposure) %>%

summarise(n = n(), .groups = "drop") %>%

group_by(CLcr_class) %>%

mutate(prop = n / sum(n) * 100)

\# 4. Graph of the proportion of patient

Proportion_graph_Poso_MDRD <-ggplot(exposure_summary_Lalagkas_MDRD_Prophylaxie, aes(x = CLcr_class, y = prop, fill = Exposure)) +

geom_bar(stat = "identity", position = "stack", width = 0.8) +

scale_y_continuous(labels = scales::percent_format(scale = 1)) +

scale_fill_manual(

values = c(

"Underexposure" = "#1f78b4", # bleu

"Acceptable exposure" = "#33a02c", # vert

"Overexposure" = "#e31a1c"	# rouge

)

) +

labs(

title = "Exposure classification by renal clearance (AUC0-24h) MDRD Formula",

x = "Renal clearance class (mL/min)",

y = "% of patients",

fill = "Exposure group"

) +

theme_minimal(base_size = 10) +

theme(

legend.position = "top",

plot.title = element_text(hjust = 0.5, size = 11, face = "bold"),

plot.subtitle = element_text(size = 9),

strip.text = element_text(size = 9)

)

Proportion_graph_Poso_MDRD

```

\# COMBINED PROPHYLAXIS

\## Chen model with the 3 different posology associated

```{r}

auc_indiv_Lalagkas_cohorte <- data_long_final_Lalagkas_CG_prophyalxie_AUC %>%

mutate(Cohorte = "Cohorte_CG")

Summary_auc_intervals_Lalagkas_EPI_cohorte <- data_long_final_Lalagkas_CKD_prophyalxie_AUC %>%

mutate(Cohorte = "Cohorte_EPI")

Summary_auc_intervals_Lalagkas_MDRD_cohorte <- data_long_final_Lalagkas_MDRD_prophyalxie_AUC %>%

mutate(Cohorte = "Cohorte_MDRD")

```

Combine all for comparaison

```{r}

\# 2. Combinaison des jeux de données

auc_indiv_all_Lalagkas_3_formula <- bind_rows(auc_indiv_Lalagkas_cohorte, Summary_auc_intervals_Lalagkas_EPI_cohorte, Summary_auc_intervals_Lalagkas_MDRD_cohorte)

auc_indiv_all_Lalagkas_3_formula$CLcr_class <- factor(auc_indiv_all_Lalagkas_3_formula$CLcr_class,

levels = c("<10", "10–24", "25–39", "40–59", "≥60"))

rmarkdown::paged_table(auc_indiv_all_Lalagkas_3_formula)

```

```{r}

\# 1. Define target intervals

auc_targets <- tibble::tibble(

Intervalle = c("AUC_0_24"),

Target_min = c(40),

Target_max = c(60)

)

\# 2. Assign exposure category

auc_with_groups_Lalagkas_3_formula <- auc_indiv_all_Lalagkas_3_formula %>%

dplyr::filter(Intervalle %in% auc_targets$Intervalle) %>%

left_join(auc_targets, by = "Intervalle") %>%

mutate(

Exposure_group = case_when(

AUC < Target_min ~ "Underexposure",

AUC > Target_max ~ "Overexposure",

TRUE	~ "Acceptable exposure"

)

)

\# 3. Summary statistics

auc_stats_table_Lalagkas_3_formula <- auc_with_groups_Lalagkas_3_formula %>%

group_by(Intervalle, Cohorte, CLcr_class) %>%

summarise(

n = n(),

median = round(median(AUC, na.rm = TRUE), 1),

Q5 = round(quantile(AUC, 0.05, na.rm = TRUE), 1),

Q25 = round(quantile(AUC, 0.25, na.rm = TRUE), 1),

Q75 = round(quantile(AUC, 0.75, na.rm = TRUE), 1),

Q95 = round(quantile(AUC, 0.95, na.rm = TRUE), 1),

n_under	= sum(Exposure_group == "Underexposure"),

n_acceptable = sum(Exposure_group == "Acceptable exposure"),

n_over	= sum(Exposure_group == "Overexposure"),

.groups = "drop"

) %>%

mutate(

Summary	= paste0(median, " [", Q5, "–", Q95, "]"),

N_Patients	= paste0("n=", n),

`Underexposure (%)` = paste0(round(100 * n_under / n, 1), "%"),

`Acceptable (%)`	= paste0(round(100 * n_acceptable / n, 1), "%"),

`Overexposure (%)`	= paste0(round(100 * n_over / n, 1), "%")

) %>%

select(Intervalle, Cohorte, CLcr_class, N_Patients, Summary,

`Underexposure (%)`, `Acceptable (%)`, `Overexposure (%)`)

\# 4. Reshape to wide format

auc_stats_table_wide_Lalagkas_3_Formula <- auc_stats_table_Lalagkas_3_formula %>%

pivot_wider(

names_from = CLcr_class,

values_from = c(N_Patients, Summary, `Underexposure (%)`, `Acceptable (%)`, `Overexposure (%)`)

) %>%

arrange(match(Intervalle, c("AUC_0_24")))

\# 5. Display in RMarkdown

rmarkdown::paged_table(auc_stats_table_wide_Lalagkas_3_Formula)

```

\## Proportion for the Chen model with the 3 different formula

```{r}

\# 1. Définir les cibles spécifiques à chaque intervalle

auc_targets <- tibble::tibble(

Intervalle = c("AUC_0_24"),

Target_min = c(40),

Target_max = c(60)

)

\# 2. Ajouter une colonne d’exposition selon la cible propre à chaque AUC

auc_classified_Lalagkas_3_formula <- auc_indiv_all_Lalagkas_3_formula %>%

left_join(auc_targets, by = "Intervalle") %>%

mutate(

Exposure = case_when(

AUC < Target_min ~ "Underexposure",

AUC >= Target_min & AUC <= Target_max ~ "Acceptable exposure",

AUC > Target_max ~ "Overexposure"

),

CLcr_class = factor(CLcr_class, levels = c("<10", "10–24", "25–39", "40–59", "≥60")),

Exposure = factor(Exposure, levels = c("Overexposure", "Acceptable exposure", "Underexposure"))

)

\# 3. Calculer les proportions

Figure_1_GA<- exposure_summary_Lalagkas_formula <- auc_classified_Lalagkas_3_formula %>%

group_by(Intervalle, Cohorte, CLcr_class, Exposure) %>%

summarise(n = n(), .groups = "drop") %>%

group_by(Intervalle, Cohorte, CLcr_class) %>%

mutate(prop = n / sum(n) * 100)

\# 4. Graphique : proportion d'exposition par Intervalle, Cohorte et CLcr_class

ggplot(exposure_summary_Lalagkas_formula, aes(x = CLcr_class, y = prop, fill = Exposure)) +

geom_bar(stat = "identity", position = "stack", width = 0.8) +

facet_grid(Intervalle ~ Cohorte) +

scale_y_continuous(labels = scales::percent_format(scale = 1)) +

scale_fill_manual(

values = c(

"Underexposure" = "#1f78b4", # bleu

"Acceptable exposure" = "#33a02c", # vert

"Overexposure" = "#e31a1c"	# rouge

)

) +

theme_minimal(base_size = 10)

Figure_1_GA

```

```{r}

library(ggplot2)

library(dplyr)

\# 1. Préparation et nettoyage des données

exposure_summary_Lalagkas_formula <- auc_classified_Lalagkas_3_formula %>%

\# Transformation des noms des cohortes et mise en ordre (Factor)

mutate(Cohorte = case_when(

Cohorte == "Cohorte_CG"	~ "Cohort CG",

Cohorte == "Cohorte_EPI" ~ "Cohort EPI",

Cohorte == "Cohorte_MDRD" ~ "Cohort MDRD",

TRUE ~ Cohorte

)) %>%

mutate(Cohorte = factor(Cohorte, levels = c("Cohort CG", "Cohort EPI", "Cohort MDRD"))) %>%

\# Calcul des effectifs par groupe

group_by(Intervalle, Cohorte, CLcr_class, Exposure) %>%

summarise(n = n(), .groups = "drop") %>%

\# Calcul du total par barre (n_total) pour les labels au-dessus

group_by(Intervalle, Cohorte, CLcr_class) %>%

mutate(

prop = n / sum(n) * 100,

n_total = sum(n)

) %>%

ungroup() %>%

\# Harmonisation du statut d'exposition pour la légende

mutate(Exposure = recode(Exposure, "Acceptable exposure" = "Target attainment"))

\# 2. Labels pour l'axe secondaire (AUC)

facet_labels <- c("AUC_0_24" = "AUC 0-24h")

\# 3. Génération du graphique

Figure_1_GA <- ggplot(exposure_summary_Lalagkas_formula,

aes(x = CLcr_class, y = n, fill = Exposure)) +

\# Barres empilées

geom_bar(stat = "identity", position = "stack", width = 0.75, color = "white", linewidth = 0.1) +

\# Chiffres (n) à l'intérieur des segments

geom_text(

aes(label = n, color = Exposure),

position = position_stack(vjust = 0.5),

size = 3.2,

fontface = "bold"

) +

\# Total (n=...) au-dessus de chaque barre

geom_text(

data = exposure_summary_Lalagkas_formula %>%

distinct(Intervalle, Cohorte, CLcr_class, n_total),

aes(x = CLcr_class, y = n_total, label = paste0("n=", n_total)),

inherit.aes = FALSE,

vjust = -0.5,

size = 3.2,

fontface = "bold"

) +

\# Organisation en facettes (Colonnes = Cohortes, Lignes = Intervalles)

facet_grid(Intervalle ~ Cohorte, labeller = labeller(Intervalle = facet_labels)) +

\# Couleurs des segments (Gris dégradés)

scale_fill_manual(

name = "Exposure Status",

values = c(

"Underexposure"	= "gray30",

"Target attainment" = "gray60",

"Overexposure"	= "gray85"

)

) +

\# Couleurs du texte dynamique pour le contraste

scale_color_manual(

values = c(

"Underexposure"	= "white",

"Target attainment" = "white",

"Overexposure"	= "black"

),

guide = "none"

) +

\# Configuration des axes

scale_y_continuous(

name = "Number of Patients",

expand = expansion(mult = c(0, 0.15)) # Espace pour les labels n=...

) +

scale_x_discrete(name = "Renal Function Category (mL/min or mL/min/1.73m2)") +

\# Thème visuel

theme_bw(base_size = 12) +

theme(

strip.text = element_text(face = "bold", size = 11),

axis.title = element_text(face = "bold"),

legend.position = "bottom",

panel.grid.minor = element_blank(),

panel.grid.major.x = element_blank()

)

\# Affichage du résultat final

Figure_1_GA

```

```{r}

tiff("Figure_2.tiff",

width = 9, height = 6.0*1,

units = "in", res = 1000,

compression = "lzw",

type = "cairo")

print(Figure_1_GA)

dev.off()

```

#Test

```{r}

library(dplyr)

library(tidyr)

library(ggplot2)

library(pracma)

library(scales)

library(stringr)

###############################################################################

\# 1) Classe fine de CLcr (PAS 1 ou 2)

###############################################################################

make_clcr_bins_pas <- function(x, step = 2, min_bin = 10, max_bin = 120) {

breaks <- seq(min_bin, max_bin, by = step)

labs <- paste0(breaks[-length(breaks)], "–", breaks[-1] - 1)

lab <- cut(

x,

breaks = breaks,

right = FALSE,

include.lowest = TRUE,

labels = labs

)

out <- as.character(lab)

out[!is.na(x) & x < min_bin] <- paste0("<", min_bin)

out[!is.na(x) & x >= max_bin] <- paste0("≥", max_bin)

levels_out <- c(paste0("<", min_bin), labs, paste0("≥", max_bin))

factor(out, levels = levels_out)

}

###############################################################################

\# 2) Fonction pour obtenir le milieu numérique de chaque bin

###############################################################################

get_bin_midpoint <- function(bin_label, min_bin = 10, max_bin = 120, step = 2) {

bin_label <- as.character(bin_label)

case_when(

is.na(bin_label) ~ NA_real_,

bin_label == paste0("<", min_bin) ~ min_bin - step / 2,

bin_label == paste0("≥", max_bin) ~ max_bin + step / 2,

str_detect(bin_label, "–") ~ {

low <- as.numeric(str_extract(bin_label, "^[0-9]+"))

high <- as.numeric(str_extract(bin_label, "(?<=–)[0-9]+"))

(low + high) / 2

},

TRUE ~ NA_real_

)

}

###############################################################################

\# 3) Table long + classes RCP + classes fines

###############################################################################

step_pas <- 2 # mettre 1 si vous voulez un pas de 1 mL/min

data_long_final_Lalagkas_CKD_prophyalxie_pas <- final_df_Lalagkas_prophylaxie_CKD_Final %>%

pivot_longer(

cols = starts_with("DFG_"),

names_to = "Formule",

names_prefix = "DFG_",

values_to = "CLcr"

) %>%

mutate(

CLcr_class = case_when(

CLcr < 10	~ "<10",

CLcr >= 10 & CLcr < 25 ~ "10–24",

CLcr >= 25 & CLcr < 40 ~ "25–39",

CLcr >= 40 & CLcr < 60 ~ "40–59",

CLcr >= 60	~ "≥60",

TRUE ~ NA_character_

),

CLcr_class_fine_pas = make_clcr_bins_pas(

CLcr,

step = step_pas,

min_bin = 10,

max_bin = 120

)

)

###############################################################################

\# 4) Calcul AUC (prophylaxie)

###############################################################################

get_indiv_auc_ranges_3_pas <- function(data_long, formule = "EPI") {

data_filtered <- data_long %>%

filter(Formule == formule)

auc_per_patient <- data_filtered %>%

group_by(ID, CLcr_class, CLcr_class_fine_pas) %>%

summarise(

AUC_0_24_pas = (pracma::trapz(time[time <= 168], DV[time <= 168])) / 7,

.groups = "drop"

) %>%

pivot_longer(

cols = starts_with("AUC"),

names_to = "Intervalle",

values_to = "AUC_pas"

)

return(auc_per_patient)

}

set.seed(100226)

data_long_final_Lalagkas_CKD_prophyalxie_AUC_pas <- get_indiv_auc_ranges_3_pas(

data_long_final_Lalagkas_CKD_prophyalxie_pas,

formule = "EPI"

)

###############################################################################

\# 5) Exclusion des outliers + classification d’exposition

###############################################################################

quantiles_pas <- quantile(

data_long_final_Lalagkas_CKD_prophyalxie_AUC_pas$AUC_pas,

probs = c(0.01, 0.99),

na.rm = TRUE

)

data_long_final_Lalagkas_CKD_prophyalxie_AUC_pas <- data_long_final_Lalagkas_CKD_prophyalxie_AUC_pas %>%

filter(AUC_pas > quantiles_pas[1], AUC_pas < quantiles_pas[2]) %>%

mutate(

Exposure_pas = case_when(

AUC_pas < 40	~ "Underexposure",

AUC_pas >= 40 & AUC_pas <= 60 ~ "Target attainment",

AUC_pas > 60	~ "Overexposure",

TRUE ~ NA_character_

),

Exposure_pas = factor(

Exposure_pas,

levels = c("Overexposure", "Target attainment", "Underexposure")

)

)

###############################################################################

\# 6) Résumé proportions par CLcr fine

###############################################################################

exposure_summary_Lalagkas_CKD_Prophylaxie_pas <- data_long_final_Lalagkas_CKD_prophyalxie_AUC_pas %>%

filter(Intervalle == "AUC_0_24_pas") %>%

group_by(CLcr_class_fine_pas, Exposure_pas) %>%

summarise(n = n(), .groups = "drop") %>%

group_by(CLcr_class_fine_pas) %>%

mutate(prop = n / sum(n) * 100) %>%

ungroup() %>%

mutate(

x_mid = get_bin_midpoint(

CLcr_class_fine_pas,

min_bin = 10,

max_bin = 120,

step = step_pas

)

)

###############################################################################

\# 7) Seuils cliniques + étiquettes

###############################################################################

thresholds <- c(10, 25, 40, 60)

vlines_df <- tibble(

xint = thresholds,

label = as.character(thresholds)

)

###############################################################################

\# 8) Figure finale

###############################################################################

###############################################################################

\# Figure S1 EPI - Version Finale (Lignes verticales limitées à 100%)

###############################################################################

library(ggplot2)

library(dplyr)

library(cowplot) # Pour le label de panneau de type publication

###############################################################################

\# Figure S1 EPI - Version Finale avec label "C"

###############################################################################

\# 1. Création de la figure de base

Figure_S1_EPI_base <- ggplot(

exposure_summary_Lalagkas_CKD_Prophylaxie_pas,

aes(x = x_mid, y = prop, fill = Exposure_pas)

) +

geom_col(

width = step_pas * 0.9,

position = position_stack(reverse = FALSE)

) +

\# Segments verticaux limités à 100%

geom_segment(

data = vlines_df,

aes(x = xint, xend = xint, y = 0, yend = 100),

inherit.aes = FALSE,

linetype = "dashed",

linewidth = 0.6,

color = "black"

) +

\# Étiquettes des seuils (10, 25, 40, 60)

geom_text(

data = vlines_df,

aes(x = xint, y = 102, label = label),

inherit.aes = FALSE,

size = 4.5,

fontface = "bold",

vjust = 0,

color = "black"

) +

\# Échelles

scale_x_continuous(

name = "Estimated Renal Function (mL/min/1.73m2) — 2 mL/min intervals",

breaks = seq(10, 120, by = 10),

expand = expansion(mult = c(0.01, 0.01))

) +

scale_y_continuous(

name = "Proportion of Patients (%)",

labels = scales::label_number(suffix = "%"),

limits = c(0, 112),

breaks = seq(0, 100, by = 20),

expand = expansion(mult = c(0, 0))

) +

scale_fill_manual(

name = "Exposure Status",

values = c(

"Underexposure"	= "gray30",

"Target attainment" = "gray60",

"Overexposure"	= "gray85"

)

) +

coord_cartesian(clip = "off") +

theme_bw(base_size = 12) +

theme(

legend.position = "top",

legend.justification = "center",

axis.title = element_text(face = "bold", size = 13),

axis.text = element_text(color = "black", size = 11, face = "bold"),

panel.grid.major.x = element_blank(),

panel.grid.minor = element_blank(),

panel.grid.major.y = element_line(color = "grey90", linewidth = 0.5),

legend.title = element_text(face = "bold"),

\# Marge supérieure pour le label C

plot.margin = margin(t = 25, r = 10, b = 10, l = 10)

)

\# 2. Ajout du label "C" en haut à gauche

Figure_S1_EPI_final <- ggdraw(Figure_S1_EPI_base) +

draw_label(

"C",

x = 0.02, y = 0.96,

hjust = 0, vjust = 1,

fontface = "bold",

size = 18

)

\# 3. Affichage

print(Figure_S1_EPI_final)

```

```{r}

tiff("Figure_S1_EPI.tiff",

width = 9, height = 6.0*1,

units = "in", res = 600,

compression = "lzw",

type = "cairo")

print(Figure_S1_EPI_final)

dev.off()

```

\## Zoom panneaux

```{r}

library(dplyr)

library(ggplot2)

set.seed(1234)

###############################################################################

\# Version “publication” — 3 panneaux (25 / 40 / 60), zoom ±4 mL/min

\# Prérequis : data_long_final_Lalagkas_CKD_prophyalxie_AUC_pas contient

\#	- Intervalle == "AUC_0_24_pas"

\#	- AUC_pas

\#	- Exposure_pas

\#	- CLcr_class_fine_pas (labels type "38–39", "40–41", etc.)

###############################################################################

\# 1) Extraire un centre numérique de bin à partir du label "a–b"

get_bin_center_pas <- function(bin_label) {

\# renvoie NA pour "<10" ou "≥120"

out <- rep(NA_real_, length(bin_label))

ok <- grepl("–", bin_label)

parts <- strsplit(bin_label[ok], "–", fixed = TRUE)

a <- as.numeric(vapply(parts, `[`, "", 1))

b <- as.numeric(vapply(parts, `[`, "", 2))

out[ok] <- (a + b) / 2

out

}

\# 2) Construire la base “zoom” autour des seuils

thresholds <- c(25, 40, 60)

window <- 4

df_zoom_pas_EPI <- data_long_final_Lalagkas_CKD_prophyalxie_AUC_pas %>%

filter(Intervalle == "AUC_0_24_pas") %>%

mutate(

CLcr_bin_center = get_bin_center_pas(as.character(CLcr_class_fine_pas))

) %>%

filter(!is.na(CLcr_bin_center)) %>%

tidyr::crossing(Threshold = thresholds) %>%

filter(CLcr_bin_center >= Threshold - window,

CLcr_bin_center <= Threshold + window) %>%

mutate(

Threshold_lab = factor(

Threshold,

levels = thresholds,

labels = paste0("Threshold ", thresholds, " mL/min (±", window, ")")

)

)

\# 3) Résumé en % (stacked à 100% par bin) — par panneau

sum_zoom_pas_EPI <- df_zoom_pas_EPI %>%

group_by(Threshold_lab, CLcr_class_fine_pas, Exposure_pas) %>%

summarise(n = n(), .groups = "drop") %>%

group_by(Threshold_lab, CLcr_class_fine_pas) %>%

mutate(prop = n / sum(n) * 100) %>%

ungroup() %>%

\# ordre des bins sur l'axe x (numérique)

mutate(

CLcr_bin_center = get_bin_center_pas(as.character(CLcr_class_fine_pas))

) %>%

arrange(Threshold_lab, CLcr_bin_center) %>%

group_by(Threshold_lab) %>%

mutate(

CLcr_class_fine_pas = factor(CLcr_class_fine_pas, levels = unique(CLcr_class_fine_pas))

) %>%

ungroup()

library(ggplot2)

library(dplyr)

library(cowplot) # Pour l'ajout du label de panneau

###############################################################################

\# Figure 2 CKD (Zoom seuils) - Version optimisée avec label "C"

###############################################################################

\# 1. Création de la figure de base

Figure_2_CKD_base <- ggplot(sum_zoom_pas_EPI,

aes(x = CLcr_class_fine_pas, y = prop, fill = Exposure_pas)) +

\# Barres avec bordure blanche fine

geom_bar(stat = "identity", position = "stack", width = 0.8, color = "white", linewidth = 0.1) +

\# Pourcentages internes

geom_text(

aes(label = ifelse(prop > 5, paste0(round(prop, 0), "%"), ""),

color = Exposure_pas),

position = position_stack(vjust = 0.5),

size = 3,

fontface = "bold"

) +

\# Séparation en 3 panneaux (25, 40, 60)

facet_wrap(~Threshold_lab, nrow = 1, scales = "free_x") +

\# Échelles

scale_y_continuous(

name = "Proportion of Patients (%)",

labels = scales::label_number(suffix = "%"),

limits = c(0, 100),

expand = expansion(mult = c(0, 0.05))

) +

scale_x_discrete(name = "Estimated Renal Function (mL/min/1.73m2)") +

\# Couleurs (Gris contrastés)

scale_fill_manual(

name = "Exposure Status",

values = c(

"Underexposure"	= "gray30",

"Target attainment" = "gray60",

"Overexposure"	= "gray85"

)

) +

scale_color_manual(

values = c(

"Underexposure"	= "white",

"Target attainment" = "white",

"Overexposure"	= "black"

),

guide = "none"

) +

\# Thème Publication

theme_bw(base_size = 12) +

theme(

legend.position = "top",

strip.background = element_rect(fill = "grey95"),

strip.text = element_text(face = "bold", size = 11),

axis.title = element_text(face = "bold"),

axis.text.y = element_text(color = "black"),

axis.text.x = element_text(angle = 90, vjust = 0.5, hjust = 1,

color = "black", size = 9, face = "bold"),

panel.grid.major.x = element_blank(),

panel.grid.minor = element_blank(),

legend.title = element_text(face = "bold"),

\# Marge pour le label C

plot.margin = margin(t = 20, r = 10, b = 10, l = 10)

)

\# 2. Ajout du label "C" en haut à gauche

Figure_2_CKD_final <- ggdraw(Figure_2_CKD_base) +

draw_label(

"C",

x = 0.02, y = 0.96,

hjust = 0, vjust = 1,

fontface = "bold",

size = 18

)

\# 3. Affichage final

print(Figure_2_CKD_final)

```

```{r}

tiff("Figure_3_CKD.tiff",

width = 9, height = 6.0*1,

units = "in", res = 600,

compression = "lzw",

type = "cairo")

print(Figure_2_CKD_final)

dev.off()

```

#Figure S1

\## Test CG

```{r}

library(dplyr)

library(tidyr)

library(ggplot2)

library(pracma)

library(scales)

library(stringr)

set.seed(1234)

###############################################################################

\# 1) Classe fine de CLcr (PAS 1 ou 2)

###############################################################################

make_clcr_bins_pas <- function(x, step = 2, min_bin = 10, max_bin = 120) {

breaks <- seq(min_bin, max_bin, by = step)

labs <- paste0(breaks[-length(breaks)], "–", breaks[-1] - 1)

lab <- cut(

x,

breaks = breaks,

right = FALSE,

include.lowest = TRUE,

labels = labs

)

out <- as.character(lab)

out[!is.na(x) & x < min_bin] <- paste0("<", min_bin)

out[!is.na(x) & x >= max_bin] <- paste0("≥", max_bin)

levels_out <- c(paste0("<", min_bin), labs, paste0("≥", max_bin))

factor(out, levels = levels_out)

}

###############################################################################

\# 2) Fonction pour obtenir le milieu numérique de chaque bin

###############################################################################

get_bin_midpoint <- function(bin_label, min_bin = 10, max_bin = 120, step = 2) {

bin_label <- as.character(bin_label)

case_when(

is.na(bin_label) ~ NA_real_,

bin_label == paste0("<", min_bin) ~ min_bin - step / 2,

bin_label == paste0("≥", max_bin) ~ max_bin + step / 2,

str_detect(bin_label, "–") ~ {

low <- as.numeric(str_extract(bin_label, "^[0-9]+"))

high <- as.numeric(str_extract(bin_label, "(?<=–)[0-9]+"))

(low + high) / 2

},

TRUE ~ NA_real_

)

}

###############################################################################

\# 3) Table long + classes RCP + classes fines

###############################################################################

step_pas <- 2 # mettre 1 si vous voulez un pas de 1 mL/min

data_long_final_Lalagkas_CG_prophyalxie_pas <- final_df_Lalagkas_prophylaxie_CG_Final %>%

pivot_longer(

cols = starts_with("DFG_"),

names_to = "Formule",

names_prefix = "DFG_",

values_to = "CLcr"

) %>%

mutate(

CLcr_class = case_when(

CLcr < 10	~ "<10",

CLcr >= 10 & CLcr < 25 ~ "10–24",

CLcr >= 25 & CLcr < 40 ~ "25–39",

CLcr >= 40 & CLcr < 60 ~ "40–59",

CLcr >= 60	~ "≥60",

TRUE ~ NA_character_

),

CLcr_class_fine_pas = make_clcr_bins_pas(

CLcr,

step = step_pas,

min_bin = 10,

max_bin = 120

)

)

###############################################################################

\# 4) Calcul AUC (prophylaxie)

###############################################################################

get_indiv_auc_ranges_5_pas <- function(data_long, formule = "CG") {

data_filtered <- data_long %>%

filter(Formule == formule)

auc_per_patient <- data_filtered %>%

group_by(ID, CLcr_class, CLcr_class_fine_pas) %>%

summarise(

AUC_0_24_pas = (pracma::trapz(time[time <= 168], DV[time <= 168])) / 7,

.groups = "drop"

) %>%

pivot_longer(

cols = starts_with("AUC"),

names_to = "Intervalle",

values_to = "AUC_pas"

)

return(auc_per_patient)

}

set.seed(100226)

data_long_final_Lalagkas_CG_prophyalxie_AUC_pas <- get_indiv_auc_ranges_5_pas(

data_long_final_Lalagkas_CG_prophyalxie_pas,

formule = "CG"

)

###############################################################################

\# 5) Exclusion des outliers + classification d’exposition

###############################################################################

quantiles_pas <- quantile(

data_long_final_Lalagkas_CG_prophyalxie_AUC_pas$AUC_pas,

probs = c(0.01, 0.99),

na.rm = TRUE

)

data_long_final_Lalagkas_CG_prophyalxie_AUC_pas <- data_long_final_Lalagkas_CG_prophyalxie_AUC_pas %>%

filter(AUC_pas > quantiles_pas[1], AUC_pas < quantiles_pas[2]) %>%

mutate(

Exposure_pas = case_when(

AUC_pas < 40	~ "Underexposure",

AUC_pas >= 40 & AUC_pas <= 60 ~ "Target attainment",

AUC_pas > 60	~ "Overexposure",

TRUE ~ NA_character_

),

Exposure_pas = factor(

Exposure_pas,

levels = c("Overexposure", "Target attainment", "Underexposure")

)

)

###############################################################################

\# 6) Résumé proportions par CLcr fine

###############################################################################

exposure_summary_Lalagkas_CG_Prophylaxie_pas <- data_long_final_Lalagkas_CG_prophyalxie_AUC_pas %>%

filter(Intervalle == "AUC_0_24_pas") %>%

group_by(CLcr_class_fine_pas, Exposure_pas) %>%

summarise(n = n(), .groups = "drop") %>%

group_by(CLcr_class_fine_pas) %>%

mutate(prop = n / sum(n) * 100) %>%

ungroup() %>%

mutate(

x_mid = get_bin_midpoint(

CLcr_class_fine_pas,

min_bin = 10,

max_bin = 120,

step = step_pas

)

)

###############################################################################

\# 7) Seuils cliniques + étiquettes

###############################################################################

thresholds <- c(10, 25, 40, 60)

vlines_df <- tibble(

xint = thresholds,

label = as.character(thresholds)

)

library(ggplot2)

library(dplyr)

library(cowplot) # Pour ajouter le label "A" proprement en dehors du graphique

###############################################################################

\# Figure S1 CG - Version optimisée avec label "A" (JAC style)

###############################################################################

\# 1. Création de la figure de base (ggplot)

Figure_S1_CG_base <- ggplot(

exposure_summary_Lalagkas_CG_Prophylaxie_pas,

aes(x = x_mid, y = prop, fill = Exposure_pas)

) +

\# Barres empilées

geom_col(

width = step_pas * 0.9,

position = position_stack(reverse = FALSE)

) +

\# Segments verticaux qui s'arrêtent à 100% (évite de barrer les chiffres)

geom_segment(

data = vlines_df,

aes(x = xint, xend = xint, y = 0, yend = 100),

inherit.aes = FALSE,

linetype = "dashed",

linewidth = 0.6,

color = "black"

) +

\# Étiquettes des seuils (10, 25, 40, 60) au-dessus des segments

geom_text(

data = vlines_df,

aes(x = xint, y = 102, label = label),

inherit.aes = FALSE,

size = 4.5,

fontface = "bold",

vjust = 0,

color = "black"

) +

\# Axe X : Lisibilité accrue (tous les 10 mL/min pour éviter l'encombrement)

scale_x_continuous(

name = "Estimated Renal Function (mL/min) — 2 mL/min intervals",

breaks = seq(10, 120, by = 10),

expand = expansion(mult = c(0.01, 0.01))

) +

\# Axe Y : Espace pour les labels de seuils

scale_y_continuous(

name = "Proportion of Patients (%)",

labels = scales::label_number(suffix = "%"),

limits = c(0, 112),

breaks = seq(0, 100, by = 20),

expand = expansion(mult = c(0, 0))

) +

\# Couleurs (Standardisées avec les autres figures)

scale_fill_manual(

name = "Exposure Status",

values = c(

"Underexposure"	= "gray30",

"Target attainment" = "gray60",

"Overexposure"	= "gray85"

)

) +

\# Empêche la coupure des éléments hors-cadre

coord_cartesian(clip = "off") +

\# Thème Publication Standard

theme_bw(base_size = 12) +

theme(

legend.position = "top",

legend.justification = "center",

axis.title = element_text(face = "bold", size = 13),

axis.text = element_text(color = "black", size = 11, face = "bold"),

panel.grid.major.x = element_blank(),

panel.grid.minor = element_blank(),

panel.grid.major.y = element_line(color = "grey90", linewidth = 0.5),

legend.title = element_text(face = "bold"),

\# Marge supérieure augmentée pour le label A

plot.margin = margin(t = 25, r = 10, b = 10, l = 10)

)

\# 2. Ajout du label "A" en haut à gauche avec cowplot

\# Cela place le label en dehors des panneaux de facettes/grilles

Figure_S1_CG_final <- ggdraw(Figure_S1_CG_base) +

draw_label(

"A", # Le texte

x = 0.02, y = 0.96, # Position (coordonnées normalisées 0-1)

hjust = 0, vjust = 1, # Alignement (top-left)

fontface = "bold", # Gras

size = 18	# Taille pour publication

)

\# 3. Affichage

print(Figure_S1_CG_final)

```

```{r}

tiff("Figure_S1_CG.tiff",

width = 9, height = 6.0*1,

units = "in", res = 600,

compression = "lzw",

type = "cairo")

print(Figure_S1_CG_final)

dev.off()

```

```{r}

library(dplyr)

library(tidyr)

library(ggplot2)

###############################################################################

\# Figure “publication” — 3 panneaux (25 / 40 / 60), zoom ±4 mL/min (CG)

\# Prérequis : data_long_final_Lalagkas_CG_prophyalxie_AUC_pas contient :

\#	- Intervalle == "AUC_0_24_pas"

\#	- AUC_pas

\#	- Exposure_pas

\#	- CLcr_class_fine_pas (labels type "38–39", "40–41", etc.)

###############################################################################

\# 1) Extraire un centre numérique de bin à partir du label "a–b"

get_bin_center_pas <- function(bin_label) {

out <- rep(NA_real_, length(bin_label))

ok <- grepl("–", bin_label, fixed = TRUE)

parts <- strsplit(bin_label[ok], "–", fixed = TRUE)

a <- as.numeric(vapply(parts, `[`, "", 1))

b <- as.numeric(vapply(parts, `[`, "", 2))

out[ok] <- (a + b) / 2

out

}

\# 2) Construire la base “zoom” autour des seuils

thresholds <- c(25, 40, 60)

window <- 4

df_zoom_pas <- data_long_final_Lalagkas_CG_prophyalxie_AUC_pas %>%

filter(Intervalle == "AUC_0_24_pas") %>%

mutate(

CLcr_bin_center = get_bin_center_pas(as.character(CLcr_class_fine_pas))

) %>%

filter(!is.na(CLcr_bin_center)) %>%

tidyr::crossing(Threshold = thresholds) %>%

filter(

CLcr_bin_center >= Threshold - window,

CLcr_bin_center <= Threshold + window

) %>%

mutate(

Threshold_lab = factor(

Threshold,

levels = thresholds,

labels = paste0("Thresold ", thresholds, " mL/min (±", window, ")")

)

)

\# 3) Résumé en % (stacked à 100% par bin) — par panneau

sum_zoom_pas <- df_zoom_pas %>%

group_by(Threshold_lab, CLcr_class_fine_pas, Exposure_pas) %>%

summarise(n = n(), .groups = "drop") %>%

group_by(Threshold_lab, CLcr_class_fine_pas) %>%

mutate(prop = n / sum(n) * 100) %>%

ungroup() %>%

\# ordre des bins sur l'axe x (numérique)

mutate(

CLcr_bin_center = get_bin_center_pas(as.character(CLcr_class_fine_pas))

) %>%

arrange(Threshold_lab, CLcr_bin_center) %>%

group_by(Threshold_lab) %>%

mutate(

CLcr_class_fine_pas = factor(CLcr_class_fine_pas, levels = unique(CLcr_class_fine_pas))

) %>%

ungroup()

library(ggplot2)

library(dplyr)

library(cowplot) # Indispensable pour l'annotation de type publication

###############################################################################

\# Figure 2 GA (Zoom seuils) - Version optimisée avec label "A"

###############################################################################

\# 1. Création de la figure de base

Figure_2_GA_base <- ggplot(sum_zoom_pas,

aes(x = CLcr_class_fine_pas, y = prop, fill = Exposure_pas)) +

\# Barres avec bordure blanche

geom_bar(stat = "identity", position = "stack", width = 0.8, color = "white", linewidth = 0.1) +

\# Labels de pourcentage internes

geom_text(

aes(label = ifelse(prop > 5, paste0(round(prop, 0), "%"), ""),

color = Exposure_pas),

position = position_stack(vjust = 0.5),

size = 3.2,

fontface = "bold"

) +

\# Panneaux par seuils

facet_wrap(~Threshold_lab, nrow = 1, scales = "free_x") +

\# Échelles

scale_y_continuous(

name = "Proportion of Patients (%)",

labels = scales::label_number(suffix = "%"),

limits = c(0, 100),

expand = expansion(mult = c(0, 0.05))

) +

scale_x_discrete(name = "Estimated Renal Function (mL/min)") +

\# Couleurs

scale_fill_manual(

name = "Exposure Status",

values = c(

"Underexposure"	= "gray30",

"Target attainment" = "gray60",

"Overexposure"	= "gray85"

)

) +

scale_color_manual(

values = c(

"Underexposure"	= "white",

"Target attainment" = "white",

"Overexposure"	= "black"

),

guide = "none"

) +

\# Thème Publication

theme_bw(base_size = 12) +

theme(

legend.position = "top",

strip.background = element_rect(fill = "grey95"),

strip.text = element_text(face = "bold", size = 11),

axis.title = element_text(face = "bold"),

axis.text.y = element_text(color = "black"),

axis.text.x = element_text(angle = 90, vjust = 0.5, hjust = 1,

color = "black", size = 9.5, face = "bold"),

panel.grid.major.x = element_blank(),

panel.grid.minor = element_blank(),

legend.title = element_text(face = "bold"),

\# Marge pour laisser de la place au "A"

plot.margin = margin(t = 20, r = 10, b = 10, l = 10)

)

\# 2. Ajout du label "A" avec cowplot

Figure_2_GA_final <- ggdraw(Figure_2_GA_base) +

draw_label(

"A",

x = 0.02, y = 0.96,

hjust = 0, vjust = 1,

fontface = "bold",

size = 18

)

\# 3. Affichage final

print(Figure_2_GA_final)

```

```{r}

tiff("Figure_3_CG.tiff",

width = 9, height = 6.0*1,

units = "in", res = 1000,

compression = "lzw",

type = "cairo")

print(Figure_2_GA_final)

dev.off()

```

```{r}

library(dplyr)

library(tidyr)

library(stringr)

library(tibble)

library(knitr)

###############################################################################

\# Discontinuity index around thresholds (CG)

\# Prérequis :

\# data_long_final_Lalagkas_CG_prophyalxie_AUC_pas contient :

\#	- Intervalle == "AUC_0_24_pas"

\#	- AUC_pas

\#	- Exposure_pas

\#	- CLcr_class_fine_pas (labels type "38–39", "40–41", etc.)

###############################################################################

\# 1) Fonction pour extraire borne basse / haute / centre depuis "a–b"

parse_bin_pas <- function(bin_label) {

low <- high <- center <- rep(NA_real_, length(bin_label))

ok <- grepl("–", bin_label, fixed = TRUE)

parts <- strsplit(bin_label[ok], "–", fixed = TRUE)

a <- as.numeric(vapply(parts, `[`, "", 1))

b <- as.numeric(vapply(parts, `[`, "", 2))

low[ok] <- a

high[ok] <- b

center[ok] <- (a + b) / 2

tibble(bin_low = low, bin_high = high, bin_center = center)

}

\# 2) Base de travail

df_disc <- data_long_final_Lalagkas_CG_prophyalxie_AUC_pas %>%

filter(Intervalle == "AUC_0_24_pas") %>%

bind_cols(parse_bin_pas(as.character(.$CLcr_class_fine_pas))) %>%

filter(!is.na(bin_low), !is.na(bin_high)) %>%

mutate(

target_attainment = Exposure_pas == "Target attainment"

)

\# 3) Résumé par bin fin

bin_summary <- df_disc %>%

group_by(CLcr_class_fine_pas, bin_low, bin_high, bin_center) %>%

summarise(

n = n(),

median_AUC = median(AUC_pas, na.rm = TRUE),

q25_AUC = quantile(AUC_pas, 0.25, na.rm = TRUE),

q75_AUC = quantile(AUC_pas, 0.75, na.rm = TRUE),

target_pct = 100 * mean(target_attainment, na.rm = TRUE),

.groups = "drop"

) %>%

arrange(bin_low, bin_high)

\# 4) Définir les bins juste sous / juste au-dessus de chaque seuil

threshold_table <- tibble(

Threshold = c(25, 40, 60),

below_low = c(23, 38, 58),

below_high = c(24, 39, 59),

above_low = c(25, 40, 60),

above_high = c(26, 41, 61)

)

\# Si tes bins sont du type 24–25 / 25–26 au lieu de 23–24 / 25–26,

\# adapte simplement les valeurs ci-dessus.

\# 5) Extraire les deux bins autour de chaque seuil

below_bins <- threshold_table %>%

left_join(

bin_summary,

by = c("below_low" = "bin_low", "below_high" = "bin_high")

) %>%

rename(

Bin_below = CLcr_class_fine_pas,

n_below = n,

median_AUC_below = median_AUC,

q25_AUC_below = q25_AUC,

q75_AUC_below = q75_AUC,

target_pct_below = target_pct

)

above_bins <- threshold_table %>%

left_join(

bin_summary,

by = c("above_low" = "bin_low", "above_high" = "bin_high")

) %>%

rename(

Bin_above = CLcr_class_fine_pas,

n_above = n,

median_AUC_above = median_AUC,

q25_AUC_above = q25_AUC,

q75_AUC_above = q75_AUC,

target_pct_above = target_pct

)

\# 6) Table finale : discontinuity

discontinuity_table <- below_bins %>%

select(

Threshold,

Bin_below, n_below, median_AUC_below, q25_AUC_below, q75_AUC_below, target_pct_below

) %>%

left_join(

above_bins %>%

select(

Threshold,

Bin_above, n_above, median_AUC_above, q25_AUC_above, q75_AUC_above, target_pct_above

),

by = "Threshold"

) %>%

mutate(

delta_target_attainment = target_pct_above - target_pct_below,

delta_median_AUC = median_AUC_above - median_AUC_below/median_AUC_above,

discontinuity_index = abs(delta_target_attainment) / abs(delta_median_AUC),

`Target below (%)` = round(target_pct_below, 1),

`Target above (%)` = round(target_pct_above, 1),

`Δ Target attainment (%)` = round(delta_target_attainment, 1),

`Median AUC below` = round(median_AUC_below, 1),

`Median AUC above` = round(median_AUC_above, 1),

`Δ Median AUC` = round(delta_median_AUC, 1),

`Discontinuity index` = round(discontinuity_index, 2)

) %>%

select(

Threshold,

Bin_below,

Bin_above,

n_below,

n_above,

`Target below (%)`,

`Target above (%)`,

`Δ Target attainment (%)`,

`Median AUC below`,

`Median AUC above`,

`Δ Median AUC`,

`Discontinuity index`

)

kable(

discontinuity_table,

align = "c",

caption = "Discontinuity in target attainment and median AUC across renal function thresholds"

)

```

\# Table target atteintenment CG

```{r}

library(dplyr)

library(tidyr)

library(tibble)

library(knitr)

\# 1) Parser les bins "a–b"

parse_bin_pas <- function(bin_label) {

low <- high <- center <- rep(NA_real_, length(bin_label))

ok <- grepl("–", bin_label, fixed = TRUE)

parts <- strsplit(bin_label[ok], "–", fixed = TRUE)

a <- as.numeric(vapply(parts, `[`, "", 1))

b <- as.numeric(vapply(parts, `[`, "", 2))

low[ok] <- a

high[ok] <- b

center[ok] <- (a + b) / 2

tibble(bin_low = low, bin_high = high, bin_center = center)

}

\# 2) Base de travail

df_disc <- data_long_final_Lalagkas_CG_prophyalxie_AUC_pas %>%

filter(Intervalle == "AUC_0_24_pas") %>%

mutate(CLcr_class_fine_pas = as.character(CLcr_class_fine_pas)) %>%

bind_cols(parse_bin_pas(.$CLcr_class_fine_pas)) %>%

filter(!is.na(bin_low), !is.na(bin_high)) %>%

mutate(target_attainment = Exposure_pas == "Target attainment")

\# 3) Résumé par bin

bin_summary <- df_disc %>%

group_by(CLcr_class_fine_pas, bin_low, bin_high, bin_center) %>%

summarise(

n = n(),

median_AUC = median(AUC_pas, na.rm = TRUE),

q25_AUC = quantile(AUC_pas, 0.25, na.rm = TRUE),

q75_AUC = quantile(AUC_pas, 0.75, na.rm = TRUE),

target_pct = 100 * mean(target_attainment, na.rm = TRUE),

.groups = "drop"

) %>%

arrange(bin_low, bin_high)

\# Vérification utile

bin_summary %>%

select(CLcr_class_fine_pas, bin_low, bin_high) %>%

arrange(bin_low) %>%

print(n = 50)

\# 4) Fonction qui trouve automatiquement les bins juste sous / juste au-dessus

find_threshold_bins <- function(threshold, bin_df) {

below <- bin_df %>%

filter(bin_high <= threshold) %>%

arrange(desc(bin_high), desc(bin_low)) %>%

slice(1)

above <- bin_df %>%

filter(bin_low >= threshold) %>%

arrange(bin_low, bin_high) %>%

slice(1)

tibble(

Threshold = threshold,

Bin_below = if (nrow(below) == 0) NA_character_ else as.character(below$CLcr_class_fine_pas[[1]]),

Bin_above = if (nrow(above) == 0) NA_character_ else as.character(above$CLcr_class_fine_pas[[1]]),

below_low = if (nrow(below) == 0) NA_real_ else below$bin_low[[1]],

below_high = if (nrow(below) == 0) NA_real_ else below$bin_high[[1]],

above_low = if (nrow(above) == 0) NA_real_ else above$bin_low[[1]],

above_high = if (nrow(above) == 0) NA_real_ else above$bin_high[[1]]

)

}

thresholds <- c(25, 40, 60)

threshold_table_auto <- bind_rows(

lapply(thresholds, find_threshold_bins, bin_df = bin_summary)

)

print(threshold_table_auto)

\# 5) Récupérer les stats des bins trouvés

below_bins <- threshold_table_auto %>%

left_join(

bin_summary,

by = c("below_low" = "bin_low", "below_high" = "bin_high")

) %>%

rename(

n_below = n,

median_AUC_below = median_AUC,

q25_AUC_below = q25_AUC,

q75_AUC_below = q75_AUC,

target_pct_below = target_pct

) %>%

select(

Threshold, Bin_below, n_below,

median_AUC_below, q25_AUC_below, q75_AUC_below, target_pct_below

)

above_bins <- threshold_table_auto %>%

left_join(

bin_summary,

by = c("above_low" = "bin_low", "above_high" = "bin_high")

) %>%

rename(

n_above = n,

median_AUC_above = median_AUC,

q25_AUC_above = q25_AUC,

q75_AUC_above = q75_AUC,

target_pct_above = target_pct

) %>%

select(

Threshold, Bin_above, n_above,

median_AUC_above, q25_AUC_above, q75_AUC_above, target_pct_above

)

\# 6) Table finale

discontinuity_table <- below_bins %>%

left_join(above_bins, by = "Threshold") %>%

mutate(

delta_target_attainment = target_pct_above - target_pct_below,

delta_median_AUC = median_AUC_above - median_AUC_below/median_AUC_above,

discontinuity_index = (delta_target_attainment) / (delta_median_AUC),

`Target below (%)` = round(target_pct_below, 1),

`Target above (%)` = round(target_pct_above, 1),

`Δ Target attainment (%)` = round(delta_target_attainment, 1),

`Median AUC below` = round(median_AUC_below, 1),

`Median AUC above` = round(median_AUC_above, 1),

`Δ Median AUC` = round(delta_median_AUC, 1),

`Discontinuity index` = round(discontinuity_index, 2)

) %>%

select(

Threshold,

Bin_below,

Bin_above,

n_below,

n_above,

`Target below (%)`,

`Target above (%)`,

`Δ Target attainment (%)`,

`Median AUC below`,

`Median AUC above`,

`Δ Median AUC`,

`Discontinuity index`

)

kable(

discontinuity_table,

align = "c",

caption = "Discontinuity in target attainment and median AUC across renal function thresholds"

)

discontinuity_table_small <- discontinuity_table %>%

select(

Threshold,

Bin_below,

Bin_above,

`Δ Target attainment (%)`,

`Δ Median AUC`,

`Discontinuity index`

)

kable(

discontinuity_table_small,

align = "c",

caption = "Summary of discontinuity across renal function thresholds"

)

```

```{r}

discontinuity_table_small <- discontinuity_table %>%

select(

Threshold,

Bin_below,

Bin_above,

`Δ Target attainment (%)`,

`Δ Median AUC`,

`Discontinuity index`

)

kable(

discontinuity_table_small,

align = "c",

caption = "Summary of discontinuity across renal function thresholds"

)

```

\# MDRD

```{r}

library(dplyr)

library(tidyr)

library(ggplot2)

library(pracma)

library(scales)

library(stringr)

###############################################################################

\# 1) Classe fine de CLcr (PAS 1 ou 2)

###############################################################################

make_clcr_bins_pas <- function(x, step = 2, min_bin = 10, max_bin = 120) {

breaks <- seq(min_bin, max_bin, by = step)

labs <- paste0(breaks[-length(breaks)], "–", breaks[-1] - 1)

lab <- cut(

x,

breaks = breaks,

right = FALSE,

include.lowest = TRUE,

labels = labs

)

out <- as.character(lab)

out[!is.na(x) & x < min_bin] <- paste0("<", min_bin)

out[!is.na(x) & x >= max_bin] <- paste0("≥", max_bin)

levels_out <- c(paste0("<", min_bin), labs, paste0("≥", max_bin))

factor(out, levels = levels_out)

}

###############################################################################

\# 2) Fonction pour extraire une position numérique de chaque classe fine

###############################################################################

get_bin_midpoint <- function(bin_label, min_bin = 10, max_bin = 120, step = 2) {

bin_label <- as.character(bin_label)

case_when(

is.na(bin_label) ~ NA_real_,

bin_label == paste0("<", min_bin) ~ min_bin - step / 2,

bin_label == paste0("≥", max_bin) ~ max_bin + step / 2,

str_detect(bin_label, "–") ~ {

low <- as.numeric(str_extract(bin_label, "^[0-9]+"))

high <- as.numeric(str_extract(bin_label, "(?<=–)[0-9]+"))

(low + high) / 2

},

TRUE ~ NA_real_

)

}

###############################################################################

\# 3) Table long + classes RCP + classes fines

###############################################################################

step_pas <- 2

data_long_final_Lalagkas_MDRD_prophyalxie_pas <- final_df_Lalagkas_prophylaxie_MDRD_Final %>%

pivot_longer(

cols = starts_with("DFG_"),

names_to = "Formule",

names_prefix = "DFG_",

values_to = "CLcr"

) %>%

mutate(

CLcr_class = case_when(

CLcr < 10	~ "<10",

CLcr >= 10 & CLcr < 25 ~ "10–24",

CLcr >= 25 & CLcr < 40 ~ "25–39",

CLcr >= 40 & CLcr < 60 ~ "40–59",

CLcr >= 60	~ "≥60",

TRUE ~ NA_character_

),

CLcr_class_fine_pas = make_clcr_bins_pas(

CLcr,

step = step_pas,

min_bin = 10,

max_bin = 120

)

)

###############################################################################

\# 4) Calcul AUC

###############################################################################

get_indiv_auc_ranges_6_pas <- function(data_long, formule = "MDRD") {

data_filtered <- data_long %>%

filter(Formule == formule)

auc_per_patient <- data_filtered %>%

group_by(ID, CLcr_class, CLcr_class_fine_pas) %>%

summarise(

AUC_0_24_pas = (pracma::trapz(time[time <= 168], DV[time <= 168])) / 7,

.groups = "drop"

) %>%

pivot_longer(

cols = starts_with("AUC"),

names_to = "Intervalle",

values_to = "AUC_pas"

)

return(auc_per_patient)

}

set.seed(100226)

data_long_final_Lalagkas_MDRD_prophyalxie_AUC_pas <- get_indiv_auc_ranges_6_pas(

data_long_final_Lalagkas_MDRD_prophyalxie_pas,

formule = "MDRD"

)

###############################################################################

\# 5) Exclusion des outliers + classification d’exposition

###############################################################################

quantiles_pas <- quantile(

data_long_final_Lalagkas_MDRD_prophyalxie_AUC_pas$AUC_pas,

probs = c(0.01, 0.99),

na.rm = TRUE

)

data_long_final_Lalagkas_MDRD_prophyalxie_AUC_pas <- data_long_final_Lalagkas_MDRD_prophyalxie_AUC_pas %>%

filter(AUC_pas > quantiles_pas[1], AUC_pas < quantiles_pas[2]) %>%

mutate(

Exposure_pas = case_when(

AUC_pas < 40	~ "Underexposure",

AUC_pas >= 40 & AUC_pas <= 60 ~ "Target attainment",

AUC_pas > 60	~ "Overexposure",

TRUE ~ NA_character_

),

Exposure_pas = factor(

Exposure_pas,

levels = c("Overexposure", "Target attainment", "Underexposure")

)

)

###############################################################################

\# 6) Résumé proportions par CLcr fine

###############################################################################

exposure_summary_Lalagkas_MDRD_Prophylaxie_pas <- data_long_final_Lalagkas_MDRD_prophyalxie_AUC_pas %>%

filter(Intervalle == "AUC_0_24_pas") %>%

group_by(CLcr_class_fine_pas, Exposure_pas) %>%

summarise(n = n(), .groups = "drop") %>%

group_by(CLcr_class_fine_pas) %>%

mutate(prop = n / sum(n) * 100) %>%

ungroup() %>%

mutate(

x_mid = get_bin_midpoint(CLcr_class_fine_pas, min_bin = 10, max_bin = 120, step = step_pas)

)

###############################################################################

\# 7) Data frame pour les lignes verticales et leurs étiquettes

###############################################################################

thresholds <- c(10, 25, 40, 60)

vlines_df <- tibble(

xint = thresholds,

label = as.character(thresholds)

)

###############################################################################

\# 8) Figure corrigée

###############################################################################

library(ggplot2)

library(dplyr)

library(cowplot)

library(scales)

###############################################################################

\# Figure S1 MDRD - Version optimisée avec label "B"

###############################################################################

\# 1. Création de la figure de base

Figure_S1_MDRD_base <- ggplot(

exposure_summary_Lalagkas_MDRD_Prophylaxie_pas,

aes(x = x_mid, y = prop, fill = Exposure_pas)

) +

\# Barres empilées (Ordre original : Under en bas)

geom_col(

width = step_pas * 0.9,

position = position_stack(reverse = FALSE)

) +

\# Segments verticaux limités à 100% (ne barre pas le texte)

geom_segment(

data = vlines_df,

aes(x = xint, xend = xint, y = 0, yend = 100),

inherit.aes = FALSE,

linetype = "dashed",

linewidth = 0.6,

color = "black"

) +

\# Étiquettes des seuils (10, 25, 40, 60)

geom_text(

data = vlines_df,

aes(x = xint, y = 102, label = label),

inherit.aes = FALSE,

size = 4.5,

fontface = "bold",

vjust = 0,

color = "black"

) +

\# Axe X : Lisibilité optimisée

scale_x_continuous(

name = "Estimated Renal Function (mL/min/1.73m2) — 2 mL/min intervals",

breaks = seq(10, 120, by = 10),

expand = expansion(mult = c(0.01, 0.01))

) +

\# Axe Y : Espace pour les labels de seuils

scale_y_continuous(

name = "Proportion of Patients (%)",

labels = scales::label_number(suffix = "%"),

limits = c(0, 112),

breaks = seq(0, 100, by = 20),

expand = expansion(mult = c(0, 0))

) +

\# Palette de gris standardisée

scale_fill_manual(

name = "Exposure Status",

values = c(

"Underexposure"	= "gray30",

"Target attainment" = "gray60",

"Overexposure"	= "gray85"

)

) +

coord_cartesian(clip = "off") +

\# Thème Publication

theme_bw(base_size = 12) +

theme(

legend.position = "top",

legend.justification = "center",

axis.title = element_text(face = "bold", size = 13),

axis.text = element_text(color = "black", size = 11, face = "bold"),

panel.grid.major.x = element_blank(),

panel.grid.minor = element_blank(),

panel.grid.major.y = element_line(color = "grey90", linewidth = 0.5),

legend.title = element_text(face = "bold"),

plot.margin = margin(t = 25, r = 10, b = 10, l = 10)

)

\# 2. Ajout du label "B" en haut à gauche

Figure_S1_MDRD_final <- ggdraw(Figure_S1_MDRD_base) +

draw_label(

"B",

x = 0.02, y = 0.96,

hjust = 0, vjust = 1,

fontface = "bold",

size = 18

)

\# 3. Affichage

print(Figure_S1_MDRD_final)

```

```{r}

tiff("Figure_S1_MDRD.tiff",

width = 9, height = 6.0*1,

units = "in", res = 600,

compression = "lzw",

type = "cairo")

print(Figure_S1_MDRD_final)

dev.off()

```

```{r}

library(dplyr)

library(tidyr)

library(ggplot2)

###############################################################################

\# Figure “publication” — 3 panneaux (25 / 40 / 60), zoom ±4 mL/min (CG)

\# Prérequis : data_long_final_Lalagkas_CG_prophyalxie_AUC_pas contient :

\#	- Intervalle == "AUC_0_24_pas"

\#	- AUC_pas

\#	- Exposure_pas

\#	- CLcr_class_fine_pas (labels type "38–39", "40–41", etc.)

###############################################################################

\# 1) Extraire un centre numérique de bin à partir du label "a–b"

get_bin_center_pas <- function(bin_label) {

out <- rep(NA_real_, length(bin_label))

ok <- grepl("–", bin_label, fixed = TRUE)

parts <- strsplit(bin_label[ok], "–", fixed = TRUE)

a <- as.numeric(vapply(parts, `[`, "", 1))

b <- as.numeric(vapply(parts, `[`, "", 2))

out[ok] <- (a + b) / 2

out

}

\# 2) Construire la base “zoom” autour des seuils

thresholds <- c(25, 40, 60)

window <- 4

df_zoom_pas_MDRD <- data_long_final_Lalagkas_MDRD_prophyalxie_AUC_pas %>%

filter(Intervalle == "AUC_0_24_pas") %>%

mutate(

CLcr_bin_center = get_bin_center_pas(as.character(CLcr_class_fine_pas))

) %>%

filter(!is.na(CLcr_bin_center)) %>%

tidyr::crossing(Threshold = thresholds) %>%

filter(

CLcr_bin_center >= Threshold - window,

CLcr_bin_center <= Threshold + window

) %>%

mutate(

Threshold_lab = factor(

Threshold,

levels = thresholds,

labels = paste0("Thresold ", thresholds, " mL/min (±", window, ")")

)

)

\# 3) Résumé en % (stacked à 100% par bin) — par panneau

sum_zoom_pas_MDRD <- df_zoom_pas_MDRD %>%

group_by(Threshold_lab, CLcr_class_fine_pas, Exposure_pas) %>%

summarise(n = n(), .groups = "drop") %>%

group_by(Threshold_lab, CLcr_class_fine_pas) %>%

mutate(prop = n / sum(n) * 100) %>%

ungroup() %>%

\# ordre des bins sur l'axe x (numérique)

mutate(

CLcr_bin_center = get_bin_center_pas(as.character(CLcr_class_fine_pas))

) %>%

arrange(Threshold_lab, CLcr_bin_center) %>%

group_by(Threshold_lab) %>%

mutate(

CLcr_class_fine_pas = factor(CLcr_class_fine_pas, levels = unique(CLcr_class_fine_pas))

) %>%

ungroup()

library(ggplot2)

library(dplyr)

library(cowplot) # Nécessaire pour placer le label "B" proprement

###############################################################################

\# Figure 2 MDRD (Zoom seuils) - Version optimisée pour publication JAC

###############################################################################

\# 1. Création de la figure de base (ggplot)

Figure_2_MDRD_base <- ggplot(sum_zoom_pas_MDRD,

aes(x = CLcr_class_fine_pas, y = prop, fill = Exposure_pas)) +

\# Barres avec bordure fine pour une séparation nette

geom_bar(stat = "identity", position = "stack", width = 0.8, color = "white", linewidth = 0.1) +

\# Pourcentages internes pour visualiser l'instabilité (cliff effect)

geom_text(

aes(label = ifelse(prop > 5, paste0(round(prop, 0), "%"), ""),

color = Exposure_pas),

position = position_stack(vjust = 0.5),

size = 3.2,

fontface = "bold"

) +

\# Panneaux par seuils

facet_wrap(~Threshold_lab, nrow = 1, scales = "free_x") +

\# Échelles

scale_y_continuous(

name = "Proportion of Patients (%)",

labels = scales::label_number(suffix = "%"),

limits = c(0, 100),

expand = expansion(mult = c(0, 0.05))

) +

scale_x_discrete(name = "Estimated Renal Function (mL/min/1.73m2)") +

\# Palette de gris harmonisée

scale_fill_manual(

name = "Exposure Status",

values = c(

"Underexposure"	= "gray30", # Gris très foncé (presque noir)

"Target attainment" = "gray60", # Gris moyen

"Overexposure"	= "gray85" # Gris très clair

)

) +

\# Couleur du texte adaptative

scale_color_manual(

values = c(

"Underexposure"	= "white",

"Target attainment" = "white",

"Overexposure"	= "black"

),

guide = "none"

) +

\# Thème Publication Standard

theme_bw(base_size = 12) +

theme(

legend.position = "top",

strip.background = element_rect(fill = "grey95"),

strip.text = element_text(face = "bold", size = 11),

axis.title = element_text(face = "bold"),

axis.text.y = element_text(color = "black"),

axis.text.x = element_text(angle = 90, vjust = 0.5, hjust = 1,

color = "black", size = 9.5, face = "bold"),

panel.grid.major.x = element_blank(),

panel.grid.minor = element_blank(),

legend.title = element_text(face = "bold"),

\# Ajout de marge supérieure pour le label B

plot.margin = margin(t = 20, r = 10, b = 10, l = 10)

)

\# 2. Ajout du label "B" en haut à gauche avec cowplot

Figure_2_MDRD_final <- ggdraw(Figure_2_MDRD_base) +

draw_label(

"B", # Le texte du label

x = 0.02, y = 0.96, # Position (coordonnées normalisées 0-1)

hjust = 0, vjust = 1, # Alignement (top-left)

fontface = "bold", # Gras

size = 18	# Taille augmentée pour lisibilité de publication

)

\# 3. Affichage final

print(Figure_2_MDRD_final)

```

```{r}

tiff("Figure_3_MDRD.tiff",

width = 9, height = 6.0*1,

units = "in", res = 600,

compression = "lzw",

type = "cairo")

print(Figure_2_MDRD_final)

dev.off()

```

\# Verification distribution population :

```{r}

library(dplyr)

library(ggplot2)

library(tidyr)

library(scales)

\# ========================

\# 1. Classes d'âge

\# ========================

full_data <- full_data %>%

mutate(

age_class = cut(

AGE,

breaks = c(20, 40, 60, 80, Inf),

right = FALSE,

labels = c("20-39", "40-59", "60-79", "80+")

)

)

\# ========================

\# 2. Distribution d'âge

\# ========================

age_summary <- full_data %>%

summarise(

n = n(),

mean_age = mean(AGE, na.rm = TRUE),

sd_age = sd(AGE, na.rm = TRUE),

median_age = median(AGE, na.rm = TRUE),

min_age = min(AGE, na.rm = TRUE),

max_age = max(AGE, na.rm = TRUE)

)

print(age_summary)

age_distribution <- full_data %>%

count(age_class) %>%

mutate(percent = 100 * n / sum(n))

print(age_distribution)

p_age <- ggplot(full_data, aes(x = AGE)) +

geom_histogram(binwidth = 5, boundary = 20, color = "black", fill = "grey80") +

labs(title = "Distribution de l'âge", x = "Âge (ans)", y = "Nombre de sujets") +

theme_bw()

print(p_age)

\# ========================

\# 3. BSA (formule de Mosteller simplifiée)

\# ========================

\# BSA (m²) = sqrt(Taille_cm x Poids_kg / 3600)

full_data <- full_data %>%

mutate(BSA = sqrt(Height * WT / 3600))

bsa_summary <- full_data %>%

summarise(

n = n(),

mean_BSA = mean(BSA, na.rm = TRUE),

sd_BSA = sd(BSA, na.rm = TRUE),

median_BSA = median(BSA, na.rm = TRUE),

min_BSA = min(BSA, na.rm = TRUE),

max_BSA = max(BSA, na.rm = TRUE)

)

print(bsa_summary)

p_bsa <- ggplot(full_data, aes(x = BSA)) +

geom_histogram(binwidth = 0.1, color = "black", fill = "grey80") +

labs(

title = "Distribution de la surface corporelle (BSA, formule de Mosteller)",

x = "BSA (m²)", y = "Nombre de sujets"

) +

theme_bw()

print(p_bsa)

\# ========================

\# 4. Distribution des DFG

\# ========================

gfr_summary <- full_data %>%

summarise(

mean_CG	= mean(DFG_CG, na.rm = TRUE),

sd_CG	= sd(DFG_CG, na.rm = TRUE),

median_CG = median(DFG_CG, na.rm = TRUE),

min_CG	= min(DFG_CG, na.rm = TRUE),

max_CG	= max(DFG_CG, na.rm = TRUE),

mean_MDRD	= mean(DFG_MDRD, na.rm = TRUE),

sd_MDRD	= sd(DFG_MDRD, na.rm = TRUE),

median_MDRD = median(DFG_MDRD, na.rm = TRUE),

min_MDRD	= min(DFG_MDRD, na.rm = TRUE),

max_MDRD	= max(DFG_MDRD, na.rm = TRUE),

mean_EPI	= mean(DFG_EPI, na.rm = TRUE),

sd_EPI	= sd(DFG_EPI, na.rm = TRUE),

median_EPI = median(DFG_EPI, na.rm = TRUE),

min_EPI	= min(DFG_EPI, na.rm = TRUE),

max_EPI	= max(DFG_EPI, na.rm = TRUE)

)

print(gfr_summary)

gfr_long <- full_data %>%

select(ID, AGE, age_class, DFG_CG, DFG_MDRD, DFG_EPI) %>%

pivot_longer(

cols = c(DFG_CG, DFG_MDRD, DFG_EPI),

names_to = "formula",

values_to = "DFG"

) %>%

mutate(

formula = recode(

formula,

DFG_CG = "Cockcroft-Gault",

DFG_MDRD = "MDRD",

DFG_EPI = "CKD-EPI"

)

)

p_gfr_hist <- ggplot(gfr_long, aes(x = DFG)) +

geom_histogram(binwidth = 10, color = "black", fill = "grey80") +

facet_wrap(~ formula, scales = "free_y") +

labs(

title = "Distribution des DFG estimés",

x = "DFG (mL/min ou mL/min/1.73 m² selon formule)",

y = "Nombre de sujets"

) +

theme_bw()

print(p_gfr_hist)

p_gfr_density <- ggplot(gfr_long, aes(x = DFG, linetype = formula)) +

geom_density(linewidth = 1) +

labs(title = "Distribution des DFG estimés", x = "DFG", y = "Densité", linetype = "Formule") +

theme_bw()

print(p_gfr_density)

\# ========================

\# 5. Pourcentage de DFG < 60 selon l'âge

\# ========================

low_gfr_by_age <- full_data %>%

group_by(age_class) %>%

summarise(

n = n(),

pct_CG_below60	= 100 * mean(DFG_CG < 60, na.rm = TRUE),

pct_MDRD_below60 = 100 * mean(DFG_MDRD < 60, na.rm = TRUE),

pct_EPI_below60 = 100 * mean(DFG_EPI < 60, na.rm = TRUE)

)

print(low_gfr_by_age)

low_gfr_by_age_long <- low_gfr_by_age %>%

pivot_longer(

cols = starts_with("pct_"),

names_to = "formula",

values_to = "percent_below60"

) %>%

mutate(

formula = recode(

formula,

pct_CG_below60 = "Cockcroft-Gault",

pct_MDRD_below60 = "MDRD",

pct_EPI_below60 = "CKD-EPI"

)

)

p_low_gfr <- ggplot(low_gfr_by_age_long, aes(x = age_class, y = percent_below60, fill = formula)) +

geom_col(position = position_dodge(width = 0.8), width = 0.7) +

scale_y_continuous(labels = label_number(suffix = "%")) +

labs(

title = "Pourcentage de sujets avec DFG < 60 selon l'âge",

x = "Classe d'âge", y = "Pourcentage", fill = "Formule"

) +

theme_bw()

print(p_low_gfr)

\# ========================

\# 6. Graphe DFG vs âge

\# ========================

p_gfr_age <- ggplot(gfr_long, aes(x = AGE, y = DFG)) +

geom_point(alpha = 0.15, size = 1) +

geom_smooth(se = TRUE, method = "loess") +

facet_wrap(~ formula, scales = "free_y") +

labs(title = "Relation entre âge et DFG estimé", x = "Âge (ans)", y = "DFG") +

theme_bw()

print(p_gfr_age)

\# ========================

\# 7. Boxplot des DFG par classe d'âge

\# ========================

p_gfr_box <- ggplot(gfr_long, aes(x = age_class, y = DFG)) +

geom_boxplot() +

facet_wrap(~ formula, scales = "free_y") +

labs(title = "Distribution des DFG par classe d'âge", x = "Classe d'âge", y = "DFG") +

theme_bw()

print(p_gfr_box)

\# ========================

\# 8. Résumé démographique du texte du manuscrit (cohorte finale)

\# ========================

\# ATTENTION : "final_cohort" doit correspondre exactement aux 9 800 patients

\# retenus après exclusion des valeurs d'AUC extrêmes (0.1e/99.9e percentile).

\# Remplace la ligne ci-dessous par le bon filtre sur les ID réellement retenus

\# (par ex. les ID présents dans df_summary_Lalagkas ou auc_by_id_CG) si ce

\# sous-ensemble diffère de full_data.

final_cohort <- full_data	# <-- à ajuster si nécessaire

demo_summary <- final_cohort %>%

summarise(

n = n(),

median_age = median(AGE, na.rm = TRUE),

min_age = min(AGE, na.rm = TRUE),

max_age = max(AGE, na.rm = TRUE),

median_wt = median(WT, na.rm = TRUE),

min_wt = min(WT, na.rm = TRUE),

max_wt = max(WT, na.rm = TRUE),

median_CG = median(DFG_CG, na.rm = TRUE),

min_CG = min(DFG_CG, na.rm = TRUE),

max_CG = max(DFG_CG, na.rm = TRUE),

median_MDRD = median(DFG_MDRD, na.rm = TRUE),

min_MDRD = min(DFG_MDRD, na.rm = TRUE),

max_MDRD = max(DFG_MDRD, na.rm = TRUE),

median_EPI = median(DFG_EPI, na.rm = TRUE),

min_EPI = min(DFG_EPI, na.rm = TRUE),

max_EPI = max(DFG_EPI, na.rm = TRUE),

pct_male = 100 * mean(SEX == 1, na.rm = TRUE),

pct_female = 100 * mean(SEX == 0, na.rm = TRUE)

)

print(demo_summary)

cat(sprintf(

"The final simulated cohort (n = %d) had a median age of %.0f years [range %.0f-%.0f], a median body weight of %.1f kg [range %.1f-%.1f], a median height of %.0f cm [range %.0f-%.0f], a median serum creatinine of %.2f mg/dL [range %.2f-%.2f], a median CrCl (Cockcroft-Gault) of %.1f mL/min [range %.1f-%.1f], a median eGFR (MDRD) of %.1f mL/min/1.73m2 [range %.1f-%.1f], a median eGFR (CKD-EPI) of %.1f mL/min/1.73m2 [range %.1f-%.1f], and comprised %.1f%% male and %.1f%% female patients.\n",

demo_summary$n,

demo_summary$median_age, demo_summary$min_age, demo_summary$max_age,

demo_summary$median_wt, demo_summary$min_wt, demo_summary$max_wt,

demo_summary$median_height, demo_summary$min_height, demo_summary$max_height,

demo_summary$median_scr, demo_summary$min_scr, demo_summary$max_scr,

demo_summary$median_CG, demo_summary$min_CG, demo_summary$max_CG,

demo_summary$median_MDRD, demo_summary$min_MDRD, demo_summary$max_MDRD,

demo_summary$median_EPI, demo_summary$min_EPI, demo_summary$max_EPI,

demo_summary$pct_male, demo_summary$pct_female

))

```

```{r}

summary(full_data$AGE)

summary(full_data$DFG_EPI)

mean(full_data$DFG_EPI < 60)

```

#Table Matrice 3

```{r}

#Table Matrice 3

library(dplyr)

library(tidyr)

library(tibble)

library(knitr)

\# ordre des classes

levels_rf <- c("≥60", "40–59", "25–39", "10–24")

\# -----------------------------------------------------------

\# Fonction générique : lignes = Cockcroft-Gault (référence)

\#	colonnes = méthode comparée (MDRD/EPI)

\#	cellules = n (% par ligne, donc % relatif à CG)

\# -----------------------------------------------------------

make_reclass_table <- function(data, row_var, col_var) {

row_sym <- rlang::ensym(row_var)

col_sym <- rlang::ensym(col_var)

\# comptages bruts

tab <- data %>%

mutate(

!!row_sym := factor(!!row_sym, levels = levels_rf),

!!col_sym := factor(!!col_sym, levels = levels_rf)

) %>%

group_by(!!row_sym, !!col_sym) %>%

summarise(n = sum(n), .groups = "drop") %>%

complete(!!row_sym := levels_rf, !!col_sym := levels_rf, fill = list(n = 0)) %>%

pivot_wider(names_from = !!col_sym, values_from = n) %>%

select(!!row_sym, all_of(levels_rf))

\# total de ligne (= marge du côté "row_var", donc CG)

tab <- tab %>%

mutate(Total = rowSums(across(all_of(levels_rf))))

\# mise en forme "n (%)" -> % calculé par rapport au total de LIGNE (CG)

tab_fmt <- tab

for (col in levels_rf) {

pct <- ifelse(tab$Total > 0,

sprintf("%.1f", 100 * tab[[col]] / tab$Total),

"0.0")

tab_fmt[[col]] <- paste0(tab[[col]], " (", pct, "%)")

}

tab_fmt$Total <- as.character(tab$Total)

\# ligne "Total" = somme des colonnes (marge du côté col_var), sans %

total_row <- tab %>%

summarise(

!!row_sym := "Total",

across(all_of(levels_rf), sum),

Total = sum(Total)

) %>%

mutate(across(all_of(levels_rf), as.character),

Total = as.character(Total))

bind_rows(tab_fmt, total_row) %>%

\# forcer l'ordre des lignes (perdu par bind_rows/as.character)

mutate(row_order = factor(!!row_sym, levels = c(levels_rf, "Total"))) %>%

arrange(row_order) %>%

select(-row_order)

}

\# ========================

\# A) CG (lignes, référence) vs MDRD (colonnes)

\# ========================

table_CG_MDRD <- make_reclass_table(df_summary_Lalagkas, CG, MDRD)

kable(

table_CG_MDRD,

align = "c",

col.names = c("Cockcroft-Gault (mL/min)", "≥60", "40–59", "25–39", "10–24", "Total"),

caption = "Table 1A. Reclassification of renal function categories: Cockcroft–Gault vs MDRD"

)

\# ========================

\# B) CG (lignes, référence) vs CKD-EPI (colonnes)

\# ========================

table_CG_EPI <- make_reclass_table(df_summary_Lalagkas, CG, EPI)

kable(

table_CG_EPI,

align = "c",

col.names = c("Cockcroft-Gault (mL/min)", "≥60", "40–59", "25–39", "10–24", "Total"),

caption = "Table 1B. Reclassification of renal function categories: Cockcroft–Gault vs CKD-EPI"

)

```

\# Table 1

```{r}

library(dplyr)

library(tidyr)

library(knitr)

\# Ordre des classes

levels_rf <- c("≥60", "40–59", "25–39", "10–24")

make_reclass_table_inv <- function(data, row_var, col_var = CG) {

row_sym <- rlang::ensym(row_var) # MDRD ou EPI (Lignes)

col_sym <- rlang::ensym(col_var) # CG (Colonnes)

\# 1. Filtre les NA et conserve uniquement les 4 classes définies

df_clean <- data %>%

filter(!is.na(!!row_sym), !is.na(!!col_sym)) %>%

filter(!!row_sym %in% levels_rf, !!col_sym %in% levels_rf)

\# 2. Comptages numériques bruts sous forme de grille

tab_num <- df_clean %>%

mutate(

!!row_sym := factor(!!row_sym, levels = levels_rf),

!!col_sym := factor(!!col_sym, levels = levels_rf)

) %>%

group_by(!!row_sym, !!col_sym) %>%

summarise(n = sum(n), .groups = "drop") %>%

complete(!!row_sym := factor(levels_rf, levels = levels_rf),

!!col_sym := factor(levels_rf, levels = levels_rf),

fill = list(n = 0)) %>%

pivot_wider(names_from = !!col_sym, values_from = n)

\# Isolement de la matrice de comptage (sans la colonne d'étiquette)

mat_vals <- as.matrix(tab_num[, levels_rf])

\# 3. Calcul des totaux

col_totals <- colSums(mat_vals)

row_totals <- rowSums(mat_vals)

grand_total <- sum(mat_vals)

\# 4. Formatage des cellules "n (col %)"

tab_fmt <- tab_num

for (j in seq_along(levels_rf)) {

col_name <- levels_rf[j]

denom <- col_totals[j]

vals <- tab_num[[col_name]]

pcts <- ifelse(denom > 0, sprintf("%.1f", 100 * vals / denom), "0.0")

tab_fmt[[col_name]] <- paste0(vals, " (", pcts, "%)")

}

\# Ajout de la colonne Total de ligne

tab_fmt$Total <- as.character(row_totals)

\# 5. Création de la ligne Total (en bas)

total_row <- data.frame(

row_label = "Total",

`≥60`	= paste0(col_totals["≥60"], " (100.0%)"),

`40–59` = paste0(col_totals["40–59"], " (100.0%)"),

`25–39` = paste0(col_totals["25–39"], " (100.0%)"),

`10–24` = paste0(col_totals["10–24"], " (100.0%)"),

Total	= as.character(grand_total),

check.names = FALSE,

stringsAsFactors = FALSE

)

colnames(total_row)[1] <- as.character(row_sym)

\# 6. Assemblage final

bind_rows(tab_fmt, total_row)

}

\# ===========================================================

\# Exécution

\# ===========================================================

table_MDRD_CG <- make_reclass_table_inv(df_summary_Lalagkas, row_var = MDRD, col_var = CG)

kable(

table_MDRD_CG,

align = "c",

col.names = c("MDRD (mL/min/1.73 m²)", "≥60", "40–59", "25–39", "10–24", "Total"),

caption = "Table 1A. Reclassification: MDRD (rows) vs Cockcroft–Gault (reference, columns)"

)

table_EPI_CG <- make_reclass_table_inv(df_summary_Lalagkas, row_var = EPI, col_var = CG)

kable(

table_EPI_CG,

align = "c",

col.names = c("CKD-EPI (mL/min/1.73 m²)", "≥60", "40–59", "25–39", "10–24", "Total"),

caption = "Table 1B. Reclassification: CKD-EPI (rows) vs Cockcroft–Gault (reference, columns)"

)

```

\# Table 2 article AUC

```{r}

\# Table 2 - Median simulated ganciclovir AUC0-24h [IQR] by renal function category

library(dplyr)

library(tidyr)

library(knitr)

levels_rf <- c("≥60", "40–59", "25–39", "10–24")

\# fonction médiane [Q1–Q3]

med_iqr <- function(x) {

q <- quantile(x, probs = c(0.5, 0.25, 0.75), na.rm = TRUE)

sprintf("%.1f [%.1f–%.1f]", q[1], q[2], q[3])

}

\# ---------------------------------------------------------

\# Fonction générique : médiane [IQR] de l'AUC par classe de

\# fonction rénale, pour une équation donnée (dosée et classée

\# selon sa propre formule)

\# ---------------------------------------------------------

make_auc_median_table <- function(data_auc, equation_name) {

data_auc %>%

filter(Intervalle == "AUC_0_24", CLcr_class %in% levels_rf) %>%

mutate(CLcr_class = factor(CLcr_class, levels = levels_rf)) %>%

group_by(CLcr_class) %>%

summarise(value = med_iqr(AUC), .groups = "drop") %>%

pivot_wider(names_from = CLcr_class, values_from = value) %>%

mutate(Equation = equation_name, .before = 1)

}

\# ---------------------------------------------------------

\# Construction des 3 lignes

\# ---------------------------------------------------------

tab_CG_auc	<- make_auc_median_table(data_long_final_Lalagkas_CG_prophyalxie_AUC, "Cockcroft-Gault")

tab_MDRD_auc <- make_auc_median_table(data_long_final_Lalagkas_MDRD_prophyalxie_AUC, "MDRD")

tab_EPI_auc <- make_auc_median_table(data_long_final_Lalagkas_CKD_prophyalxie_AUC, "CKD-EPI")

table_median_auc <- bind_rows(tab_CG_auc, tab_MDRD_auc, tab_EPI_auc) %>%

mutate(Equation = factor(Equation, levels = c("Cockcroft-Gault", "MDRD", "CKD-EPI"))) %>%

arrange(Equation) %>%

select(Equation, all_of(levels_rf))

kable(

table_median_auc,

align = "c",

col.names = c("Equation", levels_rf),

caption = "Table 2. Median simulated ganciclovir AUC0-24h [IQR] by renal function category according to Cockcroft-Gault, MDRD, and CKD-EPI equations"

)

```

\# Code Table 3

```{r}

library(dplyr)

library(tidyr)

library(knitr)

levels_rf <- c("≥60", "40–59", "25–39", "10–24")

\# ---------------------------------------------------------

\# Fonction générique : calcule le % Under/Target/Over

\# par classe de fonction rénale, pour une équation donnée

\# ---------------------------------------------------------

make_exposure_table <- function(data_auc, equation_name) {

data_auc %>%

filter(Intervalle == "AUC_0_24", CLcr_class %in% levels_rf) %>%

mutate(

CLcr_class = factor(CLcr_class, levels = levels_rf),

Exposure = case_when(

AUC < 40 ~ "Underexposure",

AUC <= 60 ~ "Target attainment",

AUC > 60 ~ "Overexposure"

),

Exposure = factor(Exposure,

levels = c("Underexposure", "Target attainment", "Overexposure"))

) %>%

group_by(CLcr_class, Exposure, .drop = FALSE) %>%

summarise(n = n(), .groups = "drop") %>%

group_by(CLcr_class) %>%

mutate(pct = round(100 * n / sum(n), 1)) %>%

ungroup() %>%

select(CLcr_class, Exposure, pct) %>%

pivot_wider(names_from = Exposure, values_from = pct, values_fill = 0) %>%

select(CLcr_class, `Underexposure`, `Target attainment`, `Overexposure`) %>%

mutate(Equation = equation_name, .before = 1) %>%

rename(`Renal function category (mL/min)` = CLcr_class,

`Underexposure (%)` = Underexposure,

`Target attainment (%)` = `Target attainment`,

`Overexposure (%)` = Overexposure)

}

\# ---------------------------------------------------------

\# Construction des 3 blocs

\# ---------------------------------------------------------

tab_CG	<- make_exposure_table(data_long_final_Lalagkas_CG_prophyalxie_AUC, "Cockcroft-Gault")

tab_MDRD <- make_exposure_table(data_long_final_Lalagkas_MDRD_prophyalxie_AUC, "MDRD")

tab_EPI <- make_exposure_table(data_long_final_Lalagkas_CKD_prophyalxie_AUC, "CKD-EPI")

table_exposure_final <- bind_rows(tab_CG, tab_MDRD, tab_EPI)

\# ---------------------------------------------------------

\# Affichage : "Equation" n'apparaît qu'à la 1ère ligne du bloc

\# ---------------------------------------------------------

table_exposure_display <- table_exposure_final %>%

group_by(Equation) %>%

mutate(Equation = ifelse(row_number() == 1, Equation, "")) %>%

ungroup()

kable(

table_exposure_display,

align = "c",

caption = "Table 3. Target attainment (%) by renal function category according to Cockcroft-Gault, MDRD, and CKD-EPI equations"

)

```

\# Erreur discretisation :

```{r}

library(dplyr)

library(pracma)

library(knitr)

\# ============================================================

\# 1) Une SEULE simulation, à la résolution fine (référence)

\# ============================================================

set.seed(050825)

sim_fine <- model_Lalagkas %>%

data_set(

full_data_Lalagkas_CKD_prophylaxie_ready %>%

select(ID, time, amt, ii, addl, ss, evid, WT, AGE, CLcr, cmt)

) %>%

zero_re("sigma") %>%

mrgsim(end = 192, delta = 0.1) %>%

as_tibble()

\# ============================================================

\# 2) "sim_coarse" = sous-échantillonnage de sim_fine

\#	(mêmes patients, mêmes ETA, juste une grille de temps plus grossière)

\# ============================================================

coarse_times <- seq(0, 192, by = 2)

sim_coarse <- sim_fine %>%

filter(time %in% coarse_times)

\# ============================================================

\# 3) Calcul de l'AUC0-24h (moyenne sur 7 jours, 0-168h)

\# ============================================================

calc_auc_0_24 <- function(sim_df) {

sim_df %>%

filter(time >= 0, time <= 168) %>%

group_by(ID) %>%

summarise(AUC_0_24 = trapz(time, DV) / 7, .groups = "drop")

}

auc_fine	<- calc_auc_0_24(sim_fine)	%>% rename(AUC_true	= AUC_0_24)

auc_coarse <- calc_auc_0_24(sim_coarse) %>% rename(AUC_delta2 = AUC_0_24)

\# ============================================================

\# 4) Comparaison

\# ============================================================

comparison_auc <- auc_fine %>%

left_join(auc_coarse, by = "ID") %>%

mutate(

abs_diff = AUC_delta2 - AUC_true,

rel_diff_pct = 100 * abs_diff / AUC_true

)

summary_discretization_error <- comparison_auc %>%

summarise(

n = n(),

mean_rel_diff_pct	= round(mean(rel_diff_pct, na.rm = TRUE), 3),

median_rel_diff_pct = round(median(rel_diff_pct, na.rm = TRUE), 3),

sd_rel_diff_pct	= round(sd(rel_diff_pct, na.rm = TRUE), 3),

max_abs_rel_diff_pct = round(max(abs(rel_diff_pct), na.rm = TRUE), 3)

)

kable(

summary_discretization_error,

align = "c",

caption = "Discretization error of trapezoidal AUC0-24h estimation (delta = 2h subsampled vs delta = 0.1h reference, same simulated patients)"

)

```

\# Test CG

```{r}

library(dplyr)

library(tidyr)

library(tibble)

library(knitr)

\# =========================================================

\# 1) Fonction AUC trapézoïdale

\# =========================================================

calc_auc_trapezoid <- function(time, conc) {

ord <- order(time)

time <- time[ord]

conc <- conc[ord]

sum(diff(time) * (head(conc, -1) + tail(conc, -1)) / 2, na.rm = TRUE)

}

\# =========================================================

\# 2) Fonction pour créer les bins exacts de 1 mL/min

\# =========================================================

make_exact_bins <- function(x) {

low <- floor(x)

high <- low + 1

label <- paste0(low, "–", high)

tibble(

bin_low = low,

bin_high = high,

CLcr_bin_exact = label

)

}

\# =========================================================

\# 3) Base de travail : CG uniquement

\# =========================================================

df_cg <- data_long_final_Lalagkas_CG_prophyalxie_pas %>%

filter(Formule == "CG") %>%

mutate(

CLcr = as.numeric(CLcr),

time = as.numeric(time),

DV = as.numeric(DV)

) %>%

filter(!is.na(ID), !is.na(CLcr), !is.na(time), !is.na(DV))

\# =========================================================

\# 4) Garder le profil sur 0-168 h

\#	puis calculer l'AUC cumulée 0-168 h

\# =========================================================

auc_by_id <- df_cg %>%

filter(time >= 0, time <= 168) %>%

group_by(ID, CLcr) %>%

summarise(

AUC_0_168 = calc_auc_trapezoid(time, DV),

.groups = "drop"

) %>%

mutate(

AUC_0_24 = AUC_0_168 / 7	# mean daily AUC, comme dans le manuscrit

)

\# =========================================================

\# 5) Définir les catégories d’exposition

\# =========================================================

auc_by_id <- auc_by_id %>%

mutate(

Exposure = case_when(

AUC_0_24 < 40 ~ "Underexposure",

AUC_0_24 <= 60 ~ "Target attainment",

AUC_0_24 > 60 ~ "Overexposure",

TRUE ~ NA_character_

),

target_attainment = Exposure == "Target attainment"

)

\# Vérification rapide

summary(auc_by_id$AUC_0_24)

\# =========================================================

\# 6) Créer les bins exacts à partir de CLcr

\# =========================================================

auc_by_id <- auc_by_id %>%

bind_cols(make_exact_bins(.$CLcr))

\# Vérification des bins demandés

auc_by_id %>%

filter(bin_low %in% c(24, 25, 39, 40, 59, 60)) %>%

count(CLcr_bin_exact, bin_low, bin_high) %>%

arrange(bin_low, bin_high)

\# =========================================================

\# 7) Résumé par bin exact

\# =========================================================

bin_summary <- auc_by_id %>%

group_by(CLcr_bin_exact, bin_low, bin_high) %>%

summarise(

n = n(),

median_AUC = median(AUC_0_24, na.rm = TRUE),

q25_AUC = quantile(AUC_0_24, 0.25, na.rm = TRUE),

q75_AUC = quantile(AUC_0_24, 0.75, na.rm = TRUE),

target_pct = 100 * mean(target_attainment, na.rm = TRUE),

.groups = "drop"

) %>%

arrange(bin_low, bin_high)

\# =========================================================

\# 8) Définir exactement les bins voulus autour des seuils

\# =========================================================

thresholds <- c(25, 40, 60)

threshold_table <- tibble(

Threshold = thresholds,

below_low = thresholds - 1,

below_high = thresholds,

above_low = thresholds,

above_high = thresholds + 1,

Bin_below = paste0(thresholds - 1, "–", thresholds),

Bin_above = paste0(thresholds, "–", thresholds + 1)

)

\# =========================================================

\# 9) Stats du bin sous le seuil

\# =========================================================

below_bins <- threshold_table %>%

left_join(

bin_summary,

by = c("below_low" = "bin_low", "below_high" = "bin_high")

) %>%

rename(

n_below = n,

median_AUC_below = median_AUC,

q25_AUC_below = q25_AUC,

q75_AUC_below = q75_AUC,

target_pct_below = target_pct

) %>%

select(

Threshold, Bin_below, n_below,

median_AUC_below, q25_AUC_below, q75_AUC_below, target_pct_below

)

\# =========================================================

\# 10) Stats du bin au-dessus du seuil

\# =========================================================

above_bins <- threshold_table %>%

left_join(

bin_summary,

by = c("above_low" = "bin_low", "above_high" = "bin_high")

) %>%

rename(

n_above = n,

median_AUC_above = median_AUC,

q25_AUC_above = q25_AUC,

q75_AUC_above = q75_AUC,

target_pct_above = target_pct

) %>%

select(

Threshold, Bin_above, n_above,

median_AUC_above, q25_AUC_above, q75_AUC_above, target_pct_above

)

\# =========================================================

\# 11) Table finale

\# =========================================================

discontinuity_table_CG <- below_bins %>%

left_join(above_bins, by = "Threshold") %>%

mutate(

delta_target_attainment = target_pct_above - target_pct_below,

delta_median_AUC = ((median_AUC_above - median_AUC_below)/median_AUC_below)*100,

discontinuity_index = (delta_target_attainment) / (delta_median_AUC),

`Target below (%)` = round(target_pct_below, 1),

`Target above (%)` = round(target_pct_above, 1),

`Δ Target attainment (%)` = round(delta_target_attainment, 1),

`Median AUC below` = round(median_AUC_below, 1),

`Median AUC above` = round(median_AUC_above, 1),

`Δ Median AUC (%)` = round(delta_median_AUC, 1),

`Discontinuity index` = round(discontinuity_index, 2)

) %>%

select(

Threshold,

Bin_below,

Bin_above,

n_below,

n_above,

`Target below (%)`,

`Target above (%)`,

`Δ Target attainment (%)`,

`Median AUC below`,

`Median AUC above`,

`Δ Median AUC (%)`,

`Discontinuity index`

)

\# =========================================================

\# 12) Affichage

\# =========================================================

kable(

discontinuity_table_CG,

align = "c",

caption = "Discontinuity in target attainment and median AUC across renal function thresholds (Cockcroft–Gault)"

)

\# =========================================================

\# 13) Version courte

\# =========================================================

discontinuity_table_CG_small <- discontinuity_table_CG %>%

select(

Threshold,

Bin_below,

Bin_above,

`Δ Target attainment (%)`,

`Δ Median AUC (%)`,

`Discontinuity index`

)

kable(

discontinuity_table_CG_small,

align = "c",

caption = "Summary of discontinuity across renal function thresholds (Cockcroft–Gault)"

)

```

```{r}

\# ===============================================================================

\# Figure S1 CG - Version avec proportions intégrées (Target Attainment Check)

\# ===============================================================================

\# 1. Création de la figure avec ajout de geom_text

Figure_S1_CG_base <- ggplot(

exposure_summary_Lalagkas_CG_Prophylaxie_pas,

aes(x = x_mid, y = prop, fill = Exposure_pas)

) +

\# Barres empilées

geom_col(

width = step_pas * 0.9,

position = position_stack(reverse = FALSE)

) +

\# AJOUT : Étiquettes de pourcentage à l'intérieur des barres

\# On ne les affiche que si la proportion est > 5% pour la clarté

geom_text(

aes(label = ifelse(prop > 5, paste0(round(prop, 0)), ""),

color = Exposure_pas), # Pour adapter la couleur du texte au fond

position = position_stack(vjust = 0.5),

size = 2.5, # Taille ajustée car les bins de 2 mL/min sont étroits

fontface = "bold",

show.legend = FALSE

) +

\# Segments verticaux aux seuils cliniques

geom_segment(

data = vlines_df,

aes(x = xint, xend = xint, y = 0, yend = 100),

inherit.aes = FALSE,

linetype = "dashed",

linewidth = 0.6,

color = "black"

) +

\# Étiquettes des seuils (10, 25, 40, 60)

geom_text(

data = vlines_df,

aes(x = xint, y = 102, label = label),

inherit.aes = FALSE,

size = 4.5,

fontface = "bold",

vjust = 0,

color = "black"

) +

\# Échelles et Labels

scale_x_continuous(

name = "Estimated Creatinine Clearance (mL/min) — 2 mL/min intervals",

breaks = seq(10, 120, by = 10),

expand = expansion(mult = c(0.01, 0.01))

) +

scale_y_continuous(

name = "Proportion of Patients (%)",

labels = scales::label_number(suffix = "%"),

limits = c(0, 112),

breaks = seq(0, 100, by = 20),

expand = expansion(mult = c(0, 0))

) +

\# Couleurs des barres

scale_fill_manual(

name = "Exposure Status",

values = c(

"Underexposure"	= "gray15",

"Target attainment" = "gray50",

"Overexposure"	= "gray85"

)

) +

\# Couleurs du texte (blanc sur fond sombre, noir sur fond clair)

scale_color_manual(

values = c(

"Underexposure"	= "white",

"Target attainment" = "white",

"Overexposure"	= "black"

)

) +

coord_cartesian(clip = "off") +

theme_bw(base_size = 12) +

theme(

legend.position = "top",

legend.justification = "center",

axis.title = element_text(face = "bold", size = 13),

axis.text = element_text(color = "black", size = 11, face = "bold"),

panel.grid.major.x = element_blank(),

panel.grid.minor = element_blank(),

panel.grid.major.y = element_line(color = "grey90", linewidth = 0.5),

legend.title = element_text(face = "bold"),

plot.margin = margin(t = 25, r = 10, b = 10, l = 10)

)

\# 2. Finalisation avec Cowplot (Label A)

Figure_S1_CG_final <- ggdraw(Figure_S1_CG_base) +

draw_label(

"A",

x = 0.02, y = 0.96,

hjust = 0, vjust = 1,

fontface = "bold",

size = 18

)

print(Figure_S1_CG_final)

```

```{r}

library(dplyr)

library(tidyr)

library(ggplot2)

library(pracma)

library(scales)

library(stringr)

library(cowplot)

library(knitr)

set.seed(1234)

\# ===============================================================================

\# 1) FONCTIONS UTILITAIRES

\# ===============================================================================

\# Calcul AUC trapézoïdale

calc_auc_trapezoid <- function(time, conc) {

ord <- order(time)

time <- time[ord]

conc <- conc[ord]

sum(diff(time) * (head(conc, -1) + tail(conc, -1)) / 2, na.rm = TRUE)

}

\# Création des bins fins

make_clcr_bins_pas <- function(x, step = 1, min_bin = 10, max_bin = 120) {

breaks <- seq(min_bin, max_bin, by = step)

labs <- paste0(breaks[-length(breaks)], "–", breaks[-1] - 1)

lab <- cut(x, breaks = breaks, right = FALSE, include.lowest = TRUE, labels = labs)

out <- as.character(lab)

out[!is.na(x) & x < min_bin] <- paste0("<", min_bin)

out[!is.na(x) & x >= max_bin] <- paste0("≥", max_bin)

levels_out <- c(paste0("<", min_bin), labs, paste0("≥", max_bin))

factor(out, levels = levels_out)

}

\# Point milieu pour l'axe X du graphique

get_bin_midpoint <- function(bin_label, min_bin = 10, max_bin = 120, step = 1) {

bin_label <- as.character(bin_label)

case_when(

is.na(bin_label) ~ NA_real_,

bin_label == paste0("<", min_bin) ~ min_bin - step / 2,

bin_label == paste0("≥", max_bin) ~ max_bin + step / 2,

str_detect(bin_label, "–") ~ {

low <- as.numeric(str_extract(bin_label, "^[0-9]+"))

high <- as.numeric(str_extract(bin_label, "(?<=–)[0-9]+"))

(low + high) / 2

},

TRUE ~ NA_real_

)

}

\# ===============================================================================

\# 2) PRÉPARATION DES DONNÉES (PAS = 1)

\# ===============================================================================

step_pas <- 1

data_long_final_Lalagkas_CG_prophyalxie_pas <- final_df_Lalagkas_prophylaxie_CG_Final %>%

pivot_longer(

cols = starts_with("DFG_"),

names_to = "Formule",

names_prefix = "DFG_",

values_to = "CLcr"

) %>%

mutate(

\# Classes RCP (nécessaires pour le group_by de ta fonction d'AUC)

CLcr_class = case_when(

CLcr < 10	~ "<10",

CLcr >= 10 & CLcr < 25 ~ "10–24",

CLcr >= 25 & CLcr < 40 ~ "25–39",

CLcr >= 40 & CLcr < 60 ~ "40–59",

CLcr >= 60	~ "≥60",

TRUE ~ NA_character_

),

\# Classes fines pour le graphique et l'index

CLcr_class_fine_pas = make_clcr_bins_pas(CLcr, step = step_pas)

)

\# Calcul de l'AUC moyenne journalière (0-168h / 7)

auc_by_id_pas <- data_long_final_Lalagkas_CG_prophyalxie_pas %>%

filter(Formule == "CG", time >= 0, time <= 168) %>%

group_by(ID, CLcr_class, CLcr_class_fine_pas) %>%

summarise(

AUC_0_24 = calc_auc_trapezoid(time, DV) / 7,

.groups = "drop"

) %>%

mutate(

Exposure = case_when(

AUC_0_24 < 40 ~ "Underexposure",

AUC_0_24 <= 60 ~ "Target attainment",

AUC_0_24 > 60 ~ "Overexposure"

),

Exposure = factor(Exposure, levels = c("Overexposure", "Target attainment", "Underexposure")),

target_attainment = (Exposure == "Target attainment")

)

\# ===============================================================================

\# 3) RÉSUMÉ STATISTIQUE PAR BIN DE 1 ML/MIN

\# ===============================================================================

exposure_summary <- auc_by_id_pas %>%

group_by(CLcr_class_fine_pas) %>%

mutate(bin_low = as.numeric(str_extract(CLcr_class_fine_pas, "^[0-9]+"))) %>%

group_by(CLcr_class_fine_pas, bin_low) %>%

summarise(

n = n(),

prop_target = mean(target_attainment) * 100,

median_auc = median(AUC_0_24),

.groups = "drop"

) %>%

mutate(x_mid = get_bin_midpoint(CLcr_class_fine_pas, step = step_pas))

\# ===============================================================================

\# 4) CALCUL DE L'INDEX DE DISCONTINUITÉ (VÉRIFICATION)

\# ===============================================================================

thresholds <- c(25, 40, 60)

discontinuity_results <- lapply(thresholds, function(th) {

below <- exposure_summary %>% filter(bin_low == th - 1)

above <- exposure_summary %>% filter(bin_low == th)

tibble(

Threshold = th,

Bin_below = below$CLcr_class_fine_pas,

Bin_above = above$CLcr_class_fine_pas,

`Target below (%)` = round(below$prop_target, 1),

`Target above (%)` = round(above$prop_target, 1),

`Δ Target attainment (%)` = round(above$prop_target - below$prop_target, 1),

`Δ Median AUC (%)` = round(((above$median_auc - below$median_auc) / above$median_auc) * 100, 1)

) %>%

mutate(`Discontinuity index` = round(`Δ Target attainment (%)` / `Δ Median AUC (%)`, 2))

}) %>% bind_rows()

print(discontinuity_results)

\# ===============================================================================

\# 5) GÉNÉRATION DE LA FIGURE S1 (PAS 1)

\# ===============================================================================

vlines_df <- tibble(xint = thresholds, label = as.character(thresholds))

\# Données pour les labels de barres (filtrées pour la lisibilité)

plot_data <- auc_by_id_pas %>%

group_by(CLcr_class_fine_pas, Exposure) %>%

summarise(n = n(), .groups = "drop") %>%

group_by(CLcr_class_fine_pas) %>%

mutate(prop = n / sum(n) * 100,

x_mid = get_bin_midpoint(CLcr_class_fine_pas, step = step_pas))

Figure_S1_CG_base <- ggplot(plot_data, aes(x = x_mid, y = prop, fill = Exposure)) +

geom_col(width = step_pas, position = position_stack(reverse = FALSE)) +

\# Labels de proportions (uniquement si > 15% car les barres de 1mL sont très fines)

geom_text(

aes(label = ifelse(prop > 15, paste0(round(prop, 0)), ""), color = Exposure),

position = position_stack(vjust = 0.5), size = 1.5, fontface = "bold", show.legend = FALSE

) +

geom_segment(data = vlines_df, aes(x = xint, xend = xint, y = 0, yend = 100),

inherit.aes = FALSE, linetype = "dashed", linewidth = 0.5) +

geom_text(data = vlines_df, aes(x = xint, y = 102, label = label),

inherit.aes = FALSE, size = 4, fontface = "bold", vjust = 0) +

scale_x_continuous(name = "Estimated Creatinine Clearance (mL/min) — 1 mL/min intervals",

breaks = seq(10, 120, by = 10), expand = c(0.01, 0.01)) +

scale_y_continuous(name = "Proportion of Patients (%)", labels = label_number(suffix = "%"),

limits = c(0, 110), breaks = seq(0, 100, by = 20), expand = c(0,0)) +

scale_fill_manual(values = c("Underexposure" = "gray15", "Target attainment" = "gray50", "Overexposure" = "gray85")) +

scale_color_manual(values = c("Underexposure" = "white", "Target attainment" = "white", "Overexposure" = "black")) +

theme_bw(base_size = 12) +

theme(legend.position = "top", panel.grid.major.x = element_blank(),

axis.text = element_text(face = "bold", color = "black"))

Figure_S1_CG_final <- ggdraw(Figure_S1_CG_base) +

draw_label("A", x = 0.02, y = 0.97, hjust = 0, vjust = 1, fontface = "bold", size = 18)

print(Figure_S1_CG_final)

```

```{r}

library(dplyr)

library(tidyr)

library(tibble)

\# =========================================================

\# Fonctions communes (déjà présentes dans ton script)

\# =========================================================

calc_auc_trapezoid <- function(time, conc) {

ord <- order(time)

time <- time[ord]

conc <- conc[ord]

sum(diff(time) * (head(conc, -1) + tail(conc, -1)) / 2, na.rm = TRUE)

}

make_exact_bins <- function(x) {

low <- floor(x)

high <- low + 1

tibble(bin_low = low, bin_high = high, CLcr_bin_exact = paste0(low, "–", high))

}

\# =========================================================

\# Fonction générique pour construire auc_by_id pour UNE équation

\# =========================================================

build_auc_by_id <- function(data_source, formule_name) {

df <- data_source %>%

filter(Formule == formule_name) %>%

mutate(

CLcr = as.numeric(CLcr),

time = as.numeric(time),

DV = as.numeric(DV)

) %>%

filter(!is.na(ID), !is.na(CLcr), !is.na(time), !is.na(DV))

auc <- df %>%

filter(time >= 0, time <= 168) %>%

group_by(ID, CLcr) %>%

summarise(AUC_0_168 = calc_auc_trapezoid(time, DV), .groups = "drop") %>%

mutate(AUC_0_24 = AUC_0_168 / 7) %>%

mutate(

Exposure = case_when(

AUC_0_24 < 40 ~ "Underexposure",

AUC_0_24 <= 60 ~ "Target attainment",

AUC_0_24 > 60 ~ "Overexposure",

TRUE ~ NA_character_

),

target_attainment = Exposure == "Target attainment"

) %>%

bind_cols(make_exact_bins(.$CLcr))

auc

}

\# =========================================================

\# Construction des 3 objets distincts (sources identifiées dans ton fichier)

\# =========================================================

auc_by_id_CG	<- build_auc_by_id(data_long_final_Lalagkas_CG_prophyalxie_pas, "CG")

auc_by_id_MDRD <- build_auc_by_id(data_long_final_Lalagkas_MDRD_prophyalxie_pas, "MDRD")

auc_by_id_EPI <- build_auc_by_id(data_long_final_Lalagkas_CKD_prophyalxie_pas, "EPI")

\# Vérifications rapides

summary(auc_by_id_CG$AUC_0_24)

summary(auc_by_id_MDRD$AUC_0_24)

summary(auc_by_id_EPI$AUC_0_24)

nrow(auc_by_id_CG)

nrow(auc_by_id_MDRD)

nrow(auc_by_id_EPI)

```

#Bootstrap

```{r}

library(dplyr)

library(tidyr)

library(purrr)

library(knitr)

\# =========================================================

\# Fonction : calcule le DI pour un jeu de données donné

\# (réutilisable telle quelle sur un échantillon bootstrap)

\# =========================================================

compute_DI_table <- function(auc_by_id) {

thresholds <- c(25, 40, 60)

bin_summary <- auc_by_id %>%

group_by(bin_low, bin_high) %>%

summarise(

n = n(),

median_AUC = median(AUC_0_24, na.rm = TRUE),

target_pct = 100 * mean(target_attainment, na.rm = TRUE),

.groups = "drop"

)

threshold_table <- tibble(

Threshold = thresholds,

below_low = thresholds - 1, below_high = thresholds,

above_low = thresholds, above_high = thresholds + 1

)

below_bins <- threshold_table %>%

left_join(bin_summary, by = c("below_low" = "bin_low", "below_high" = "bin_high")) %>%

rename(n_below = n, median_AUC_below = median_AUC, target_pct_below = target_pct) %>%

select(Threshold, n_below, median_AUC_below, target_pct_below)

above_bins <- threshold_table %>%

left_join(bin_summary, by = c("above_low" = "bin_low", "above_high" = "bin_high")) %>%

rename(n_above = n, median_AUC_above = median_AUC, target_pct_above = target_pct) %>%

select(Threshold, n_above, median_AUC_above, target_pct_above)

below_bins %>%

left_join(above_bins, by = "Threshold") %>%

mutate(

delta_target = target_pct_above - target_pct_below,

delta_AUC_pct = (median_AUC_above - median_AUC_below) / median_AUC_below * 100,

DI = delta_target / delta_AUC_pct

) %>%

select(Threshold, n_below, n_above, delta_target, delta_AUC_pct, DI)

}

\# =========================================================

\# Fonction : bootstrap du DI pour UNE équation

\# =========================================================

bootstrap_DI <- function(auc_by_id, n_boot = 1000, seed = 123) {

set.seed(seed)

n_patients <- nrow(auc_by_id)

boot_results <- map_dfr(1:n_boot, function(i) {

\# rééchantillonnage avec remise (patients, pas juste les lignes de bin)

boot_sample <- auc_by_id[sample(1:n_patients, n_patients, replace = TRUE), ]

compute_DI_table(boot_sample) %>%

mutate(replicate = i)

})

boot_results

}

\# =========================================================

\# Application aux 3 équations

\# (auc_by_id_CG / auc_by_id_MDRD / auc_by_id_EPI doivent

\# déjà contenir : AUC_0_24, target_attainment, bin_low, bin_high)

\# =========================================================

boot_CG	<- bootstrap_DI(auc_by_id_CG, n_boot = 1000)

boot_MDRD <- bootstrap_DI(auc_by_id_MDRD, n_boot = 1000)

boot_EPI <- bootstrap_DI(auc_by_id_EPI, n_boot = 1000)

\# =========================================================

\# Résumé : point estimate (échantillon original) + IC bootstrap

\# =========================================================

summarize_DI_bootstrap <- function(auc_by_id, boot_results, equation_name) {

point_estimate <- compute_DI_table(auc_by_id) %>%

select(Threshold, DI_point = DI)

boot_summary <- boot_results %>%

group_by(Threshold) %>%

summarise(

DI_median_boot = median(DI, na.rm = TRUE),

DI_ci_low = quantile(DI, 0.025, na.rm = TRUE),

DI_ci_high = quantile(DI, 0.975, na.rm = TRUE),

DI_sd_boot = sd(DI, na.rm = TRUE),

.groups = "drop"

)

point_estimate %>%

left_join(boot_summary, by = "Threshold") %>%

mutate(Equation = equation_name, .before = 1)

}

DI_summary_CG <- summarize_DI_bootstrap(auc_by_id_CG, boot_CG, "Cockcroft-Gault")

DI_summary_MDRD <- summarize_DI_bootstrap(auc_by_id_MDRD, boot_MDRD, "MDRD")

DI_summary_EPI <- summarize_DI_bootstrap(auc_by_id_EPI, boot_EPI, "CKD-EPI")

DI_summary_final <- bind_rows(DI_summary_CG, DI_summary_MDRD, DI_summary_EPI) %>%

mutate(

`DI [point estimate]` = round(DI_point, 2),

`Bootstrap median DI` = round(DI_median_boot, 2),

`95% CI` = paste0("[", round(DI_ci_low, 2), "; ", round(DI_ci_high, 2), "]"),

`Bootstrap SD` = round(DI_sd_boot, 2)

) %>%

select(Equation, Threshold, `DI [point estimate]`, `Bootstrap median DI`, `95% CI`, `Bootstrap SD`)

kable(

DI_summary_final,

align = "c",

caption = "Table 4. Discontinuity Index (DI) with bootstrap 95% confidence intervals (1000 replicates) across renal function thresholds"

)

```

```{r}

\# Point estimates cohérents avec le bootstrap (à partir des mêmes données n=10000)

compute_DI_table(auc_by_id_CG)

compute_DI_table(auc_by_id_MDRD)

compute_DI_table(auc_by_id_EPI)

```

```{r}

library(ggplot2)

library(dplyr)

\# Zoom sur la plage cliniquement pertinente + légende propre

plot_range <- auc_by_id_CG %>% filter(CLcr <= 120)

ggplot(plot_range, aes(x = CLcr, y = AUC_0_24)) +

geom_point(alpha = 0.04, size = 0.4, color = "grey30") +

geom_smooth(method = "loess", span = 0.08, color = "black", se = TRUE, fill = "grey60") +

geom_hline(aes(yintercept = 40, linetype = "AUC target range (40-60)"), color = "red") +

geom_hline(yintercept = 60, linetype = "dashed", color = "red") +

geom_vline(aes(xintercept = 25, linetype = "Dosing thresholds"), color = "blue") +

geom_vline(xintercept = c(40, 60), linetype = "dotted", color = "blue") +

scale_linetype_manual(name = "", values = c("AUC target range (40-60)" = "dashed",

"Dosing thresholds" = "dotted")) +

coord_cartesian(xlim = c(0, 120), ylim = c(0, 150)) +

labs(

x = "Cockcroft-Gault renal function (mL/min)",

y = "Simulated AUC0-24h (mg.h/L)",

title = "Continuous relationship between renal function and simulated exposure"

) +

theme_bw() +

theme(legend.position = "bottom")

```

\# Panel 3

```{r}

library(ggplot2)

library(dplyr)

\# Empiler les 3 datasets avec un identifiant d'équation

plot_data_all <- bind_rows(

auc_by_id_CG	%>% mutate(Equation = "Cockcroft-Gault"),

auc_by_id_MDRD %>% mutate(Equation = "MDRD"),

auc_by_id_EPI %>% mutate(Equation = "CKD-EPI")

) %>%

mutate(Equation = factor(Equation, levels = c("Cockcroft-Gault", "MDRD", "CKD-EPI"))) %>%

filter(CLcr <= 120)

Figure_3_revised <- ggplot(plot_data_all, aes(x = CLcr, y = AUC_0_24)) +

geom_point(alpha = 0.04, size = 0.4, color = "grey30") +

geom_smooth(method = "loess", span = 0.08, color = "black", se = TRUE, fill = "grey60") +

geom_hline(aes(yintercept = 40, linetype = "AUC target range (40-60)"), color = "red") +

geom_hline(yintercept = 60, linetype = "dashed", color = "red") +

geom_vline(aes(xintercept = 25, linetype = "Dosing thresholds"), color = "blue") +

geom_vline(xintercept = c(40, 60), linetype = "dotted", color = "blue") +

scale_linetype_manual(name = "", values = c("AUC target range (40-60)" = "dashed",

"Dosing thresholds" = "dotted")) +

facet_wrap(~ Equation, ncol = 1) +

coord_cartesian(xlim = c(0, 120), ylim = c(0, 150)) +

labs(

x = "Estimated renal function (mL/min or mL/min/1.73 m²)",

y = "Simulated AUC0-24h (mg.h/L)",

) +

theme_bw() +

theme(legend.position = "bottom", strip.text = element_text(face = "bold"))

Figure_3_revised

```

```{r}

tiff("Figure_3_new.tiff",

width = 9, height = 6.0*1,

units = "in", res = 600,

compression = "lzw",

type = "cairo")

print(Figure_3_revised)

dev.off()

```

```{r}

library(dplyr)

library(tidyr)

library(tibble)

library(knitr)

levels_rf <- c("≥60", "40–59", "25–39", "10–24")

\# ============================================================

\# 1) Matrice de comptages brute (CG en lignes = référence,

\#	col_var en colonnes = MDRD ou EPI), ordre FORCÉ

\# ============================================================

get_raw_matrix <- function(data, col_var) {

col_sym <- rlang::ensym(col_var)

tab <- data %>%

mutate(

CG = factor(CG, levels = levels_rf),

!!col_sym := factor(!!col_sym, levels = levels_rf)

) %>%

group_by(CG, !!col_sym) %>%

summarise(n = sum(n), .groups = "drop") %>%

complete(CG = levels_rf, !!col_sym := levels_rf, fill = list(n = 0)) %>%

pivot_wider(names_from = !!col_sym, values_from = n) %>%

select(CG, all_of(levels_rf)) %>%

column_to_rownames("CG") %>%

as.matrix()

\# Forcer explicitement l'ordre lignes ET colonnes (sécurité)

tab <- tab[levels_rf, levels_rf]

tab

}

mat_MDRD <- get_raw_matrix(df_summary_Lalagkas, MDRD)

mat_EPI <- get_raw_matrix(df_summary_Lalagkas, EPI)

\# Vérification visuelle avant de calculer les stats

print(mat_MDRD)

print(mat_EPI)

\# ============================================================

\# 2) Statistiques de reclassification à partir d'une matrice

\#	(lignes = CG/référence, colonnes = équation comparée)

\#	levels_rf est ordonné du MEILLEUR au PIRE (≥60 -> 10-24),

\#	donc "downward" = déplacement vers un indice de colonne plus grand

\# ============================================================

reclass_stats <- function(mat) {

n_levels <- nrow(mat)

total_n <- sum(mat)

concordant <- sum(diag(mat))

downward <- sum(mat[row(mat) < col(mat)])

upward	<- sum(mat[row(mat) > col(mat)])

pct_reclassified <- 100 * (total_n - concordant) / total_n

pct_downward <- 100 * downward / total_n

pct_upward	<- 100 * upward / total_n

\# % downgradé/upgradé PAR catégorie de départ, avec garde-fou sur les bornes

pct_downgraded_vec <- sapply(1:n_levels, function(i) {

if (i < n_levels) {

sum(mat[i, (i+1):n_levels]) / sum(mat[i, ]) * 100

} else {

0

}

})

pct_upgraded_vec <- sapply(1:n_levels, function(i) {

if (i > 1) {

sum(mat[i, 1:(i-1)]) / sum(mat[i, ]) * 100

} else {

0

}

})

per_category <- tibble(

CG_category = rownames(mat),

n_total = rowSums(mat),

pct_downgraded = pct_downgraded_vec,

pct_upgraded = pct_upgraded_vec,

pct_concordant = 100 * diag(mat) / rowSums(mat)

)

list(

pct_reclassified = round(pct_reclassified, 1),

pct_downward = round(pct_downward, 1),

pct_upward = round(pct_upward, 1),

per_category = per_category %>% mutate(across(where(is.numeric), ~round(., 1)))

)

}

stats_MDRD <- reclass_stats(mat_MDRD)

stats_EPI <- reclass_stats(mat_EPI)

cat("=== MDRD vs Cockcroft-Gault ===\n")

cat("Overall reclassified:", stats_MDRD$pct_reclassified, "%\n")

cat("Downward:", stats_MDRD$pct_downward, "% | Upward:", stats_MDRD$pct_upward, "%\n\n")

kable(stats_MDRD$per_category, caption = "MDRD: reclassification by starting CG category")

cat("\n=== CKD-EPI vs Cockcroft-Gault ===\n")

cat("Overall reclassified:", stats_EPI$pct_reclassified, "%\n")

cat("Downward:", stats_EPI$pct_downward, "% | Upward:", stats_EPI$pct_upward, "%\n\n")

kable(stats_EPI$per_category, caption = "CKD-EPI: reclassification by starting CG category")

```

\# Test CKD

```{r}

library(dplyr)

library(tidyr)

library(tibble)

library(knitr)

\# =========================================================

\# 1) Fonction AUC trapézoïdale

\# =========================================================

calc_auc_trapezoid <- function(time, conc) {

ord <- order(time)

time <- time[ord]

conc <- conc[ord]

sum(diff(time) * (head(conc, -1) + tail(conc, -1)) / 2, na.rm = TRUE)

}

\# =========================================================

\# 2) Fonction pour créer les bins exacts de 1 mL/min

\# =========================================================

make_exact_bins <- function(x) {

low <- floor(x)

high <- low + 1

label <- paste0(low, "–", high)

tibble(

bin_low = low,

bin_high = high,

CLcr_bin_exact = label

)

}

\# =========================================================

\# 3) Base de travail : CG uniquement

\# =========================================================

df_cKD <- data_long_final_Lalagkas_CKD_prophyalxie_pas %>%

filter(Formule == "EPI") %>%

mutate(

CLcr = as.numeric(CLcr),

time = as.numeric(time),

DV = as.numeric(DV)

) %>%

filter(!is.na(ID), !is.na(CLcr), !is.na(time), !is.na(DV))

\# =========================================================

\# 4) Garder le profil sur 0-168 h

\#	puis calculer l'AUC cumulée 0-168 h

\# =========================================================

auc_by_id <- df_cKD %>%

filter(time >= 0, time <= 168) %>%

group_by(ID, CLcr) %>%

summarise(

AUC_0_168 = calc_auc_trapezoid(time, DV),

.groups = "drop"

) %>%

mutate(

AUC_0_24 = AUC_0_168 / 7	# mean daily AUC, comme dans le manuscrit

)

\# =========================================================

\# 5) Définir les catégories d’exposition

\# =========================================================

auc_by_id <- auc_by_id %>%

mutate(

Exposure = case_when(

AUC_0_24 < 40 ~ "Underexposure",

AUC_0_24 <= 60 ~ "Target attainment",

AUC_0_24 > 60 ~ "Overexposure",

TRUE ~ NA_character_

),

target_attainment = Exposure == "Target attainment"

)

\# Vérification rapide

summary(auc_by_id$AUC_0_24)

\# =========================================================

\# 6) Créer les bins exacts à partir de CLcr

\# =========================================================

auc_by_id <- auc_by_id %>%

bind_cols(make_exact_bins(.$CLcr))

\# Vérification des bins demandés

auc_by_id %>%

filter(bin_low %in% c(24, 25, 39, 40, 59, 60)) %>%

count(CLcr_bin_exact, bin_low, bin_high) %>%

arrange(bin_low, bin_high)

\# =========================================================

\# 7) Résumé par bin exact

\# =========================================================

bin_summary <- auc_by_id %>%

group_by(CLcr_bin_exact, bin_low, bin_high) %>%

summarise(

n = n(),

median_AUC = median(AUC_0_24, na.rm = TRUE),

q25_AUC = quantile(AUC_0_24, 0.25, na.rm = TRUE),

q75_AUC = quantile(AUC_0_24, 0.75, na.rm = TRUE),

target_pct = 100 * mean(target_attainment, na.rm = TRUE),

.groups = "drop"

) %>%

arrange(bin_low, bin_high)

\# =========================================================

\# 8) Définir exactement les bins voulus autour des seuils

\# =========================================================

thresholds <- c(25, 40, 60)

threshold_table <- tibble(

Threshold = thresholds,

below_low = thresholds - 1,

below_high = thresholds,

above_low = thresholds,

above_high = thresholds + 1,

Bin_below = paste0(thresholds - 1, "–", thresholds),

Bin_above = paste0(thresholds, "–", thresholds + 1)

)

\# =========================================================

\# 9) Stats du bin sous le seuil

\# =========================================================

below_bins <- threshold_table %>%

left_join(

bin_summary,

by = c("below_low" = "bin_low", "below_high" = "bin_high")

) %>%

rename(

n_below = n,

median_AUC_below = median_AUC,

q25_AUC_below = q25_AUC,

q75_AUC_below = q75_AUC,

target_pct_below = target_pct

) %>%

select(

Threshold, Bin_below, n_below,

median_AUC_below, q25_AUC_below, q75_AUC_below, target_pct_below

)

\# =========================================================

\# 10) Stats du bin au-dessus du seuil

\# =========================================================

above_bins <- threshold_table %>%

left_join(

bin_summary,

by = c("above_low" = "bin_low", "above_high" = "bin_high")

) %>%

rename(

n_above = n,

median_AUC_above = median_AUC,

q25_AUC_above = q25_AUC,

q75_AUC_above = q75_AUC,

target_pct_above = target_pct

) %>%

select(

Threshold, Bin_above, n_above,

median_AUC_above, q25_AUC_above, q75_AUC_above, target_pct_above

)

\# =========================================================

\# 11) Table finale

\# =========================================================

discontinuity_table_CKD <- below_bins %>%

left_join(above_bins, by = "Threshold") %>%

mutate(

delta_target_attainment = target_pct_above - target_pct_below,

delta_median_AUC = ((median_AUC_above - median_AUC_below)/median_AUC_below)*100,

discontinuity_index = (delta_target_attainment) / (delta_median_AUC),

`Target below (%)` = round(target_pct_below, 1),

`Target above (%)` = round(target_pct_above, 1),

`Δ Target attainment (%)` = round(delta_target_attainment, 1),

`Median AUC below` = round(median_AUC_below, 1),

`Median AUC above` = round(median_AUC_above, 1),

`Δ Median AUC (%)` = round(delta_median_AUC, 1),

`Discontinuity index` = round(discontinuity_index, 3)

) %>%

select(

Threshold,

Bin_below,

Bin_above,

n_below,

n_above,

`Target below (%)`,

`Target above (%)`,

`Δ Target attainment (%)`,

`Median AUC below`,

`Median AUC above`,

`Δ Median AUC (%)`,

`Discontinuity index`

)

\# =========================================================

\# 12) Affichage

\# =========================================================

kable(

discontinuity_table_CKD,

align = "c",

caption = "Discontinuity in target attainment and median AUC across renal function thresholds (CKD-EPI)"

)

\# =========================================================

\# 13) Version courte

\# =========================================================

discontinuity_table_CKD_small <- discontinuity_table_CKD %>%

select(

Threshold,

Bin_below,

Bin_above,

`Δ Target attainment (%)`,

`Δ Median AUC (%)`,

`Discontinuity index`

)

kable(

discontinuity_table_CKD_small,

align = "c",

caption = "Summary of discontinuity across renal function thresholds (CKD-EPI)"

)

```

\# Test MDRD

```{r}

library(dplyr)

library(tidyr)

library(tibble)

library(knitr)

\# =========================================================

\# 1) Fonction AUC trapézoïdale

\# =========================================================

calc_auc_trapezoid <- function(time, conc) {

ord <- order(time)

time <- time[ord]

conc <- conc[ord]

sum(diff(time) * (head(conc, -1) + tail(conc, -1)) / 2, na.rm = TRUE)

}

\# =========================================================

\# 2) Fonction pour créer les bins exacts de 1 mL/min

\# =========================================================

make_exact_bins <- function(x) {

low <- floor(x)

high <- low + 1

label <- paste0(low, "–", high)

tibble(

bin_low = low,

bin_high = high,

CLcr_bin_exact = label

)

}

\# =========================================================

\# 3) Base de travail : CG uniquement

\# =========================================================

df_cg <- data_long_final_Lalagkas_MDRD_prophyalxie_pas %>%

filter(Formule == "MDRD") %>%

mutate(

CLcr = as.numeric(CLcr),

time = as.numeric(time),

DV = as.numeric(DV)

) %>%

filter(!is.na(ID), !is.na(CLcr), !is.na(time), !is.na(DV))

\# =========================================================

\# 4) Garder le profil sur 0-168 h

\#	puis calculer l'AUC cumulée 0-168 h

\# =========================================================

auc_by_id <- df_cg %>%

filter(time >= 0, time <= 168) %>%

group_by(ID, CLcr) %>%

summarise(

AUC_0_168 = calc_auc_trapezoid(time, DV),

.groups = "drop"

) %>%

mutate(

AUC_0_24 = AUC_0_168 / 7	# mean daily AUC, comme dans le manuscrit

)

\# =========================================================

\# 5) Définir les catégories d’exposition

\# =========================================================

auc_by_id <- auc_by_id %>%

mutate(

Exposure = case_when(

AUC_0_24 < 40 ~ "Underexposure",

AUC_0_24 <= 60 ~ "Target attainment",

AUC_0_24 > 60 ~ "Overexposure",

TRUE ~ NA_character_

),

target_attainment = Exposure == "Target attainment"

)

\# Vérification rapide

summary(auc_by_id$AUC_0_24)

\# =========================================================

\# 6) Créer les bins exacts à partir de CLcr

\# =========================================================

auc_by_id <- auc_by_id %>%

bind_cols(make_exact_bins(.$CLcr))

\# Vérification des bins demandés

auc_by_id %>%

filter(bin_low %in% c(24, 25, 39, 40, 59, 60)) %>%

count(CLcr_bin_exact, bin_low, bin_high) %>%

arrange(bin_low, bin_high)

\# =========================================================

\# 7) Résumé par bin exact

\# =========================================================

bin_summary <- auc_by_id %>%

group_by(CLcr_bin_exact, bin_low, bin_high) %>%

summarise(

n = n(),

median_AUC = median(AUC_0_24, na.rm = TRUE),

q25_AUC = quantile(AUC_0_24, 0.25, na.rm = TRUE),

q75_AUC = quantile(AUC_0_24, 0.75, na.rm = TRUE),

target_pct = 100 * mean(target_attainment, na.rm = TRUE),

.groups = "drop"

) %>%

arrange(bin_low, bin_high)

\# =========================================================

\# 8) Définir exactement les bins voulus autour des seuils

\# =========================================================

thresholds <- c(25, 40, 60)

threshold_table <- tibble(

Threshold = thresholds,

below_low = thresholds - 1,

below_high = thresholds,

above_low = thresholds,

above_high = thresholds + 1,

Bin_below = paste0(thresholds - 1, "–", thresholds),

Bin_above = paste0(thresholds, "–", thresholds + 1)

)

\# =========================================================

\# 9) Stats du bin sous le seuil

\# =========================================================

below_bins <- threshold_table %>%

left_join(

bin_summary,

by = c("below_low" = "bin_low", "below_high" = "bin_high")

) %>%

rename(

n_below = n,

median_AUC_below = median_AUC,

q25_AUC_below = q25_AUC,

q75_AUC_below = q75_AUC,

target_pct_below = target_pct

) %>%

select(

Threshold, Bin_below, n_below,

median_AUC_below, q25_AUC_below, q75_AUC_below, target_pct_below

)

\# =========================================================

\# 10) Stats du bin au-dessus du seuil

\# =========================================================

above_bins <- threshold_table %>%

left_join(

bin_summary,

by = c("above_low" = "bin_low", "above_high" = "bin_high")

) %>%

rename(

n_above = n,

median_AUC_above = median_AUC,

q25_AUC_above = q25_AUC,

q75_AUC_above = q75_AUC,

target_pct_above = target_pct

) %>%

select(

Threshold, Bin_above, n_above,

median_AUC_above, q25_AUC_above, q75_AUC_above, target_pct_above

)

\# =========================================================

\# 11) Table finale

\# =========================================================

discontinuity_table_MDRD <- below_bins %>%

left_join(above_bins, by = "Threshold") %>%

mutate(

delta_target_attainment = target_pct_above - target_pct_below,

delta_median_AUC = ((median_AUC_above - median_AUC_below)/median_AUC_below)*100,# Variation relative sur l'AUC médiane

discontinuity_index = (delta_target_attainment) / (delta_median_AUC),

`Target below (%)` = round(target_pct_below, 1),

`Target above (%)` = round(target_pct_above, 1),

`Δ Target attainment (%)` = round(delta_target_attainment, 1),

`Median AUC below` = round(median_AUC_below, 1),

`Median AUC above` = round(median_AUC_above, 1),

`Δ Median AUC (%)` = round(delta_median_AUC, 1),

`Discontinuity index` = round(discontinuity_index, 2)

) %>%

select(

Threshold,

Bin_below,

Bin_above,

n_below,

n_above,

`Target below (%)`,

`Target above (%)`,

`Δ Target attainment (%)`,

`Median AUC below`,

`Median AUC above`,

`Δ Median AUC (%)`,

`Discontinuity index`

)

\# =========================================================

\# 12) Affichage

\# =========================================================

kable(

discontinuity_table_MDRD,

align = "c",

caption = "Discontinuity in target attainment and median AUC across renal function thresholds (MDRD)"

)

\# =========================================================

\# 13) Version courte

\# =========================================================

discontinuity_table_MDRD_small <- discontinuity_table_MDRD %>%

select(

Threshold,

Bin_below,

Bin_above,

`Δ Target attainment (%)`,

`Δ Median AUC (%)`,

`Discontinuity index`

)

kable(

discontinuity_table_MDRD_small,

align = "c",

caption = "Summary of discontinuity across renal function thresholds (MDRD)"

)

```

```{r}

library(dplyr)

library(tidyr)

library(tibble)

library(knitr)

\# =========================================================

\# 1) Fonctions de base

\# =========================================================

calc_auc_trapezoid <- function(time, conc) {

ord <- order(time)

time <- time[ord]

conc <- conc[ord]

sum(diff(time) * (head(conc, -1) + tail(conc, -1)) / 2, na.rm = TRUE)

}

make_exact_bins <- function(x) {

low <- floor(x)

high <- low + 1

tibble(bin_low = low, bin_high = high, CLcr_bin_exact = paste0(low, "–", high))

}

\# =========================================================

\# 2) Préparation des données (CG)

\# =========================================================

df_cg <- data_long_final_Lalagkas_CG_prophyalxie_pas %>%

filter(Formule == "CG") %>%

mutate(across(c(CLcr, time, DV), as.numeric)) %>%

filter(!is.na(ID), !is.na(CLcr), !is.na(time), !is.na(DV))

\# Calcul AUC 0-24h (moyenne sur 7 jours)

auc_by_id <- df_cg %>%

filter(time >= 0, time <= 168) %>%

group_by(ID, CLcr) %>%

summarise(AUC_0_24 = calc_auc_trapezoid(time, DV) / 7, .groups = "drop") %>%

mutate(

Exposure = case_when(

AUC_0_24 < 40 ~ "Underexposure",

AUC_0_24 <= 60 ~ "Target attainment",

AUC_0_24 > 60 ~ "Overexposure"

),

target_attainment = (Exposure == "Target attainment")

) %>%

bind_cols(make_exact_bins(.$CLcr))

\# =========================================================

\# 3) Résumé par bin de 1 mL/min

\# =========================================================

bin_summary <- auc_by_id %>%

group_by(CLcr_bin_exact, bin_low, bin_high) %>%

summarise(

n = n(),

median_AUC = median(AUC_0_24, na.rm = TRUE),

target_pct = 100 * mean(target_attainment, na.rm = TRUE),

.groups = "drop"

)

\# =========================================================

\# 4) Analyse de discontinuité (Pas de 1 autour des seuils)

\# =========================================================

thresholds <- c(25, 40, 60)

\# Construction de la table de comparaison (Bins strictement adjacents)

threshold_comparison <- tibble(

Threshold = thresholds,

low_b = thresholds - 1, # Borne basse du bin "Below"

high_b = thresholds, # Borne haute du bin "Below"

low_a = thresholds, # Borne basse du bin "Above"

high_a = thresholds + 1 # Borne haute du bin "Above"

)

\# Récupération des stats "Below" et "Above"

discontinuity_table <- threshold_comparison %>%

\# Jointure pour le bin juste en-dessous

left_join(bin_summary, by = c("low_b" = "bin_low", "high_b" = "bin_high")) %>%

rename(Bin_below = CLcr_bin_exact, n_below = n,

med_below = median_AUC, target_below = target_pct) %>%

\# Jointure pour le bin juste au-dessus

left_join(bin_summary, by = c("low_a" = "bin_low", "high_a" = "bin_high")) %>%

rename(Bin_above = CLcr_bin_exact, n_above = n,

med_above = median_AUC, target_above = target_pct) %>%

\# Calculs des indices

mutate(

`Δ Target attainment (%)` = round(target_above - target_below, 1),

`Δ Median AUC (%)` = round(((med_above - med_below) / med_above) * 100, 1),

`Discontinuity index` = round(`Δ Target attainment (%)` / `Δ Median AUC (%)`, 2)

)

\# =========================================================

\# 5) Affichage final

\# =========================================================

discontinuity_table %>%

select(Threshold, Bin_below, Bin_above, n_below, n_above,

`Δ Target attainment (%)`, `Δ Median AUC (%)`, `Discontinuity index`) %>%

kable(align = "c", caption = "Discontinuity Analysis (Step: 1 mL/min)")

```

```{r}

library(dplyr)

library(purrr)

library(mrgsolve)

library(pracma)

library(ggplot2)

\# ============================================================

\# 1) Population de base : DFG_CG renommé en CLcr (nom attendu

\#	par le modèle mrgsolve), filtré sur la plage 10-100 mL/min

\# ============================================================

pop_base <- full_data %>%

filter(DFG_CG >= 10, DFG_CG <= 100) %>%

select(ID, WT, DFG_CG) %>%

rename(CLcr = DFG_CG)

\# ============================================================

\# 2) Les 4 schémas de dose fixes (appliqués à TOUTE la population,

\#	indépendamment de la catégorie de dosage officielle)

\# ============================================================

regimens <- tibble::tibble(

Regimen = factor(

c("Full dose (900 mg q24h)",

"Half dose (450 mg q24h)",

"450 mg q48h",

"450 mg q84h"),

levels = c("Full dose (900 mg q24h)",

"Half dose (450 mg q24h)",

"450 mg q48h",

"450 mg q84h")

),

amt = c(900, 450, 450, 450),

ii = c(24, 24, 48, 84)

)

\# ============================================================

\# 3) Fonction de simulation d'AUC0-24h pour UN schéma donné

\#	(cohérente avec la pipeline principale : end=192, delta=2,

\#	addl=9, pas de zero_re("sigma"))

\# ============================================================

simulate_auc_fixed_regimen <- function(pop, amt_dose, ii_dose, model, seed = 123) {

data_ev <- pop %>%

mutate(

time = 0, evid = 1, cmt = 1, ss = 1,

amt = amt_dose, ii = ii_dose, addl = 9

)

set.seed(seed)

out <- model %>%

data_set(data_ev) %>%

mrgsim(end = 192, delta = 2) %>%

as_tibble()

out %>%

filter(time >= 0, time <= 168) %>%

group_by(ID) %>%

summarise(AUC_0_24 = trapz(time, DV) / 7, .groups = "drop")

}

\# ============================================================

\# 4) Vérification de cohérence AVANT de lancer la boucle complète

\#	(doit être proche de 42.9, valeur Table 2, catégorie 25-39, CG)

\# ============================================================

test_patients <- pop_base %>% filter(CLcr >= 28, CLcr <= 32)

test <- simulate_auc_fixed_regimen(test_patients, amt_dose = 450, ii_dose = 48, model_Lalagkas)

cat("Médiane de vérification (attendu ~42.9) :", median(test$AUC_0_24), "\n")

\# ============================================================

\# 5) Boucle sur les 4 régimes, appliqués à toute la population

\# ============================================================

results_list <- map_dfr(1:nrow(regimens), function(i) {

reg <- regimens[i, ]

simulate_auc_fixed_regimen(pop_base, reg$amt, reg$ii, model_Lalagkas) %>%

left_join(pop_base, by = "ID") %>%

mutate(Regimen = reg$Regimen)

})

\# ============================================================

\# 6) Graphique : AUC vs CLcr (10-100), 4 courbes lissées, SANS seuils

\# ============================================================

ggplot(results_list, aes(x = CLcr, y = AUC_0_24, color = Regimen, fill = Regimen)) +

geom_smooth(method = "loess", span = 0.3, se = TRUE, linewidth = 1) +

scale_x_continuous(limits = c(10, 100), breaks = seq(10, 100, 10)) +

labs(

x = "Renal function (Cockcroft-Gault, mL/min)",

y = "Simulated AUC0-24h (mg.h/L)",

color = "Dosing regimen", fill = "Dosing regimen",

title = "Simulated ganciclovir exposure across renal function under fixed dosing regimens"

) +

theme_bw() +

theme(legend.position = "bottom")

```

```{r}

library(dplyr)

library(ggplot2)

library(purrr)

\# ============================================================

\# 1) Médiane par bin de 1 mL/min (reprend le calcul précédent)

\# ============================================================

results_binned <- results_list %>%

mutate(bin = floor(CLcr)) %>%

group_by(Regimen, bin) %>%

summarise(median_AUC = median(AUC_0_24, na.rm = TRUE), .groups = "drop")

\# ============================================================

\# 2) Pour chaque régime, ajuster un LOESS et prédire sur une

\#	grille fine de CLcr, pour identifier précisément l'intervalle

\#	où l'AUC médiane lissée est dans [40, 60]

\# ============================================================

find_target_range <- function(data_regimen, grid = seq(10, 100, by = 0.1)) {

fit <- loess(median_AUC ~ bin, data = data_regimen, span = 0.3)

pred <- predict(fit, newdata = data.frame(bin = grid))

in_target <- grid[which(pred >= 40 & pred <= 60)]

if (length(in_target) == 0) {

return(tibble(CLcr_min = NA, CLcr_max = NA, note = "Never within 40-60 mg.h/L over 10-100 mL/min"))

}

tibble(

CLcr_min = round(min(in_target), 1),

CLcr_max = round(max(in_target), 1),

note = ifelse(

length(in_target) == length(which(grid >= min(in_target) & grid <= max(in_target))),

"Continuous range",

"Non-continuous range \u2014 check plot"

)

)

}

target_ranges <- results_binned %>%

group_by(Regimen) %>%

group_modify(~ find_target_range(.x)) %>%

ungroup()

print(target_ranges)

\# ============================================================

\# 3) Graphique avec la bande cible et les intervalles identifiés

\#	surlignés pour chaque régime (sans lignes de seuil de dose)

\# ============================================================

ggplot(results_binned, aes(x = bin, y = median_AUC, color = Regimen)) +

annotate("rect", xmin = -Inf, xmax = Inf, ymin = 40, ymax = 60,

fill = "grey70", alpha = 0.25) +

geom_smooth(method = "loess", span = 0.3, se = FALSE, linewidth = 1) +

scale_x_continuous(limits = c(10, 100), breaks = seq(10, 100, 10)) +

labs(

x = "Renal function (Cockcroft-Gault, mL/min)",

y = "Median simulated AUC0-24h (mg.h/L)",

color = "Dosing regimen",

title = "Renal function range achieving target AUC0-24h (40-60 mg.h/L) by dosing regimen"

) +

theme_bw() +

theme(legend.position = "bottom")

```

```{r}

library(dplyr)

library(ggplot2)

gaps <- tibble::tibble(

gap_start = c(17.8, 31.0, 62.1),

gap_end	= c(20.9, 38.3, 86.0)

)

ggplot(results_binned, aes(x = bin, y = median_AUC, color = Regimen)) +

annotate("rect", xmin = -Inf, xmax = Inf, ymin = 40, ymax = 60,

fill = "grey70", alpha = 0.25) +

geom_rect(data = gaps, inherit.aes = FALSE,

aes(xmin = gap_start, xmax = gap_end, ymin = -Inf, ymax = Inf),

fill = "red", alpha = 0.12) +

geom_smooth(method = "loess", span = 0.3, se = FALSE, linewidth = 1) +

scale_x_continuous(limits = c(10, 100), breaks = seq(10, 100, 10)) +

scale_y_log10(

breaks = c(10, 20, 40, 60, 80, 100, 150, 200),

limits = c(NA, 200),

oob = scales::oob_squish # tronque proprement au lieu de faire disparaître la ligne

) +

labs(

x = "Renal function (Cockcroft-Gault, mL/min)",

y = "Median simulated AUC0-24h (mg.h/L, log scale)",

color = "Dosing regimen",

title = "Renal function coverage gaps across fixed dosing regimens",

subtitle = "Red bands: no tested regimen achieves the AUC target (40-60 mg.h/L).\nY-axis capped at 200 mg.h/L; full-dose values exceed this at low renal function."

) +

theme_bw() +

theme(legend.position = "bottom")

```

```{r}

library(dplyr)

library(ggplot2)

\# ============================================================

\# 1) Médiane + Q10/Q90 par bin de 1 mL/min, pour chaque régime

\# ============================================================

results_binned_q <- results_list %>%

mutate(bin = floor(CLcr)) %>%

group_by(Regimen, bin) %>%

summarise(

median_AUC = median(AUC_0_24, na.rm = TRUE),

q10_AUC	= quantile(AUC_0_24, 0.10, na.rm = TRUE),

q90_AUC	= quantile(AUC_0_24, 0.90, na.rm = TRUE),

.groups = "drop"

)

\# ============================================================

\# 2) Lissage LOESS séparé pour la médiane et pour les bornes Q10/Q90

\#	(pour garder des courbes lisses cohérentes avec le reste du papier)

\# ============================================================

smooth_quantile <- function(data_regimen, grid = seq(10, 100, by = 1)) {

fit_med <- loess(median_AUC ~ bin, data = data_regimen, span = 0.3)

fit_q10 <- loess(q10_AUC	~ bin, data = data_regimen, span = 0.3)

fit_q90 <- loess(q90_AUC	~ bin, data = data_regimen, span = 0.3)

tibble(

bin = grid,

median_AUC = predict(fit_med, newdata = data.frame(bin = grid)),

q10_AUC	= predict(fit_q10, newdata = data.frame(bin = grid)),

q90_AUC	= predict(fit_q90, newdata = data.frame(bin = grid))

)

}

results_smoothed <- results_binned_q %>%

group_by(Regimen) %>%

group_modify(~ smooth_quantile(.x)) %>%

ungroup()

\# ============================================================

\# 3) Graphique : médiane (ligne) + bande Q10-Q90 (ruban semi-transparent)

\# ============================================================

library(dplyr)

library(ggplot2)

gaps <- tibble::tibble(

gap_start = c(17.8, 31.0, 62.1),

gap_end	= c(20.9, 38.3, 86.0)

)

Figure_sup <- ggplot(results_smoothed, aes(x = bin, y = median_AUC, color = Regimen)) +

annotate("rect", xmin = -Inf, xmax = Inf, ymin = 40, ymax = 60,

fill = "grey85", alpha = 0.6) +

geom_rect(data = gaps, inherit.aes = FALSE,

aes(xmin = gap_start, xmax = gap_end, ymin = -Inf, ymax = Inf),

fill = "red", alpha = 0.10) +

geom_line(aes(y = q10_AUC), linetype = "dashed", linewidth = 0.4, alpha = 0.7) +

geom_line(aes(y = q90_AUC), linetype = "dashed", linewidth = 0.4, alpha = 0.7) +

geom_line(linewidth = 1.1) +

scale_x_continuous(limits = c(10, 100), breaks = seq(10, 100, 10)) +

scale_y_log10(

breaks = c(10, 20, 40, 60, 80, 100, 150, 200),

limits = c(NA, 200),

oob = scales::oob_squish

) +

labs(

x = "Renal function (Cockcroft-Gault, mL/min)",

y = "Simulated AUC0-24h (mg.h/L, log scale)",

color = "Dosing regimen",

title = "Renal function coverage gaps across fixed dosing regimens",

subtitle = "Solid line: median; dashed lines: 10th-90th percentile.\nGrey band: AUC target (40-60 mg.h/L). Red bands: no regimen achieves target.\nY-axis capped at 200 mg.h/L."

) +

theme_bw() +

theme(legend.position = "bottom")

Figure_sup

```

```{r}

tiff("Figure_sup.tiff",

width = 9, height = 6,

units = "in", res = 600,

compression = "lzw",

type = "cairo")

print(Figure_sup)

dev.off()

```

```{r}

library(dplyr)

library(ggplot2)

library(purrr)

\# ============================================================

\# 0) Rappel : fonction de simulation (déjà corrigée précédemment)

\# ============================================================

simulate_auc_fixed_regimen <- function(pop, amt_dose, ii_dose, model, seed = 123) {

data_ev <- pop %>%

mutate(

time = 0, evid = 1, cmt = 1, ss = 1,

amt = amt_dose, ii = ii_dose, addl = 9

)

set.seed(seed)

out <- model %>%

data_set(data_ev) %>%

mrgsim(end = 192, delta = 2) %>%

as_tibble()

out %>%

filter(time >= 0, time <= 168) %>%

group_by(ID) %>%

summarise(AUC_0_24 = trapz(time, DV) / 7, .groups = "drop")

}

regimens <- tibble::tibble(

Regimen = factor(

c("Full dose (900 mg q24h)", "Half dose (450 mg q24h)", "450 mg q48h", "450 mg q84h"),

levels = c("Full dose (900 mg q24h)", "Half dose (450 mg q24h)", "450 mg q48h", "450 mg q84h")

),

amt = c(900, 450, 450, 450),

ii = c(24, 24, 48, 84)

)

\# ============================================================

\# Fonction générique : construit la population, simule les 4 régimes,

\# identifie l'intervalle cible, et trace le graphique — pour UNE équation

\# ============================================================

run_target_range_analysis <- function(full_data, dfg_col, equation_label, unit_label) {

pop_base <- full_data %>%

filter(.data[[dfg_col]] >= 10, .data[[dfg_col]] <= 100) %>%

select(ID, WT, all_of(dfg_col)) %>%

rename(CLcr = all_of(dfg_col))

results_list <- map_dfr(1:nrow(regimens), function(i) {

reg <- regimens[i, ]

simulate_auc_fixed_regimen(pop_base, reg$amt, reg$ii, model_Lalagkas) %>%

left_join(pop_base, by = "ID") %>%

mutate(Regimen = reg$Regimen)

})

results_binned <- results_list %>%

mutate(bin = floor(CLcr)) %>%

group_by(Regimen, bin) %>%

summarise(median_AUC = median(AUC_0_24, na.rm = TRUE), .groups = "drop")

find_target_range <- function(data_regimen, grid = seq(10, 100, by = 0.1)) {

fit <- loess(median_AUC ~ bin, data = data_regimen, span = 0.3)

pred <- predict(fit, newdata = data.frame(bin = grid))

in_target <- grid[which(pred >= 40 & pred <= 60)]

if (length(in_target) == 0) {

return(tibble(CLcr_min = NA, CLcr_max = NA, note = "Never within 40-60 mg.h/L over 10-100 mL/min"))

}

tibble(

CLcr_min = round(min(in_target), 1),

CLcr_max = round(max(in_target), 1),

note = ifelse(

length(in_target) == length(which(grid >= min(in_target) & grid <= max(in_target))),

"Continuous range", "Non-continuous range \u2014 check plot"

)

)

}

target_ranges <- results_binned %>%

group_by(Regimen) %>%

group_modify(~ find_target_range(.x)) %>%

ungroup()

cat("\n===", equation_label, "===\n")

print(target_ranges)

p <- ggplot(results_binned, aes(x = bin, y = median_AUC, color = Regimen)) +

annotate("rect", xmin = -Inf, xmax = Inf, ymin = 40, ymax = 60,

fill = "grey70", alpha = 0.25) +

geom_smooth(method = "loess", span = 0.3, se = FALSE, linewidth = 1) +

scale_x_continuous(limits = c(10, 100), breaks = seq(10, 100, 10)) +

labs(

x = paste0("Renal function (", equation_label, ", ", unit_label, ")"),

y = "Median simulated AUC0-24h (mg.h/L)",

color = "Dosing regimen",

title = paste0("Renal function range achieving target AUC0-24h (40-60 mg.h/L) \u2014 ", equation_label)

) +

theme_bw() +

theme(legend.position = "bottom")

print(p)

list(target_ranges = target_ranges, plot = p, results_binned = results_binned)

}

\# ============================================================

\# Exécution pour MDRD et CKD-EPI

\# ============================================================

res_MDRD <- run_target_range_analysis(full_data, "DFG_MDRD", "MDRD", "mL/min/1.73m2")

res_EPI <- run_target_range_analysis(full_data, "DFG_EPI", "CKD-EPI", "mL/min/1.73m2")

```

\# Supplemental table 3

```{r}

library(dplyr)

library(ggplot2)

library(tidyr)

library(scales)

\# ============================================================

\# 0) Données : réutilise UNIQUEMENT res_* du chunk Supplemental Table 3

\#	(ne PAS redéfinir run_target_range_analysis ici)

\# ============================================================

stopifnot(exists("res_CG"), exists("res_MDRD"), exists("res_EPI"))

eq_levels <- c("Cockcroft-Gault", "MDRD", "CKD-EPI")

relabel_eq <- function(x) {

factor(dplyr::recode(as.character(x), "CG" = "Cockcroft-Gault"), levels = eq_levels)

}

regimen_colors <- c(

"Full dose (900 mg q24h)" = "#F8766D",

"Half dose (450 mg q24h)" = "#7CAE00",

"450 mg q48h"	= "#00BFC4",

"450 mg q84h"	= "#C77CFF"

)

all_target_ranges_fig <- bind_rows(res_CG$target_ranges,

res_MDRD$target_ranges,

res_EPI$target_ranges) %>%

mutate(Equation = relabel_eq(Equation),

Regimen = factor(as.character(Regimen), levels = names(regimen_colors)))

smoothed_all <- bind_rows(res_CG$smoothed, res_MDRD$smoothed, res_EPI$smoothed) %>%

mutate(Equation = relabel_eq(Equation),

Regimen = factor(as.character(Regimen), levels = names(regimen_colors)))

\# Contrôle : aucune NA, et CG demi-dose jusqu'à ~49 / pleine dose dès ~67

stopifnot(!any(is.na(all_target_ranges_fig$Equation)))

print(as.data.frame(all_target_ranges_fig %>%

mutate(across(c(CLcr_min, CLcr_max), ~ round(.x, 1))) %>%

arrange(Equation, Regimen)))

\# ============================================================

\# 1) Gaps (toutes zones sans régime en cible)

\# ============================================================

compute_gaps <- function(target_ranges, grid = seq(10, 100, by = 0.1)) {

in_any_target <- rep(FALSE, length(grid))

for (i in seq_len(nrow(target_ranges))) {

lo <- target_ranges$CLcr_min[i]; hi <- target_ranges$CLcr_max[i]

if (!is.na(lo)) in_any_target <- in_any_target | (grid >= lo & grid <= hi)

}

gap_grid <- grid[!in_any_target]

if (length(gap_grid) == 0) return(tibble(gap_start = numeric(0), gap_end = numeric(0)))

tibble(g = gap_grid, grp = cumsum(c(1, diff(gap_grid) > 0.15))) %>%

group_by(grp) %>%

summarise(gap_start = min(g), gap_end = max(g), .groups = "drop") %>%

select(-grp) %>%

filter(gap_end - gap_start > 0.5)

}

gaps_all <- all_target_ranges_fig %>%

group_by(Equation) %>%

group_modify(~ compute_gaps(.x)) %>%

ungroup() %>%

mutate(gap_width = round(abs(gap_end - gap_start), 1))

\# ============================================================

\# 2) Points de croisement demi-dose / pleine dose

\# ============================================================

crossing_points <- all_target_ranges_fig %>%

filter(Regimen %in% c("Full dose (900 mg q24h)", "Half dose (450 mg q24h)")) %>%

mutate(Regimen = as.character(Regimen)) %>%

select(Equation, Regimen, CLcr_min, CLcr_max) %>%

pivot_wider(names_from = Regimen, values_from = c(CLcr_min, CLcr_max)) %>%

transmute(

Equation,

full_dose_enters_target = `CLcr_min_Full dose (900 mg q24h)`,

half_dose_exits_target = `CLcr_max_Half dose (450 mg q24h)`,

gap_start = half_dose_exits_target,

gap_end	= full_dose_enters_target,

gap_width = gap_end - gap_start

)

cat("\n=== Crossing points ===\n")

print(as.data.frame(crossing_points))

crossing_points_long <- bind_rows(

crossing_points %>% transmute(Equation, x = full_dose_enters_target, y = 60,

Regimen = "Full dose (900 mg q24h)"),

crossing_points %>% transmute(Equation, x = half_dose_exits_target, y = 40,

Regimen = "Half dose (450 mg q24h)")

) %>%

mutate(Regimen = factor(Regimen, levels = names(regimen_colors)))

gap_annotations <- crossing_points %>%

filter(!is.na(gap_width), gap_width > 0) %>%

mutate(y_annot = 8,

label = paste0(round(gap_start), "\u2013", round(gap_end),

" (", round(gap_width), ")"))

gaps_all_edge <- gaps_all %>%

left_join(gap_annotations %>% select(Equation, c_start = gap_start, c_end = gap_end),

by = "Equation") %>%

filter(is.na(c_start) | !(abs(gap_start - c_start) < 2 & abs(gap_end - c_end) < 2)) %>%

select(-c_start, -c_end)

\# ============================================================

\# 3) Figure 4

\# ============================================================

col_60 <- regimen_colors[["Full dose (900 mg q24h)"]]

col_40 <- regimen_colors[["Half dose (450 mg q24h)"]]

col_25 <- regimen_colors[["450 mg q48h"]]

Figure_4 <- ggplot(smoothed_all, aes(x = x, color = Regimen)) +

annotate("rect", xmin = -Inf, xmax = Inf, ymin = 40, ymax = 60,

fill = "grey70", alpha = 0.25) +

geom_rect(data = gaps_all, inherit.aes = FALSE,

aes(xmin = gap_start, xmax = gap_end, ymin = 1, ymax = 500),

fill = "red", alpha = 0.15, color = NA) +

geom_vline(xintercept = 60, color = col_60, linetype = "dotted", linewidth = 0.6) +

geom_vline(xintercept = 40, color = col_40, linetype = "dotted", linewidth = 0.6) +

geom_vline(xintercept = 25, color = col_25, linetype = "dotted", linewidth = 0.6) +

geom_line(aes(y = q10_AUC), linetype = "dashed", linewidth = 0.4, alpha = 0.7) +

geom_line(aes(y = q90_AUC), linetype = "dashed", linewidth = 0.4, alpha = 0.7) +

geom_line(aes(y = median_AUC), linewidth = 1.1) +

geom_point(data = crossing_points_long, aes(x = x, y = y, color = Regimen),

size = 2.6, shape = 21, fill = "white", stroke = 1.3, inherit.aes = FALSE) +

geom_segment(data = gap_annotations, inherit.aes = FALSE,

aes(x = gap_start, xend = gap_end, y = y_annot, yend = y_annot),

arrow = arrow(ends = "both", length = unit(0.1, "cm")),

color = "red4", linewidth = 0.5) +

geom_text(data = gap_annotations, inherit.aes = FALSE,

aes(x = (gap_start + gap_end) / 2, y = y_annot * 0.75, label = label),

color = "red4", size = 3) +

geom_label(data = gaps_all_edge, inherit.aes = FALSE,

aes(x = (gap_start + gap_end) / 2, y = 13, label = round(gap_width)),

color = "red4", size = 2.5, fontface = "bold",

label.size = 0, fill = "white", alpha = 0.75,

label.padding = unit(0.1, "lines")) +

facet_wrap(~ Equation, ncol = 1) +

scale_x_continuous(limits = c(10, 100), breaks = seq(10, 100, 10)) +

scale_y_log10(breaks = c(10, 20, 40, 60, 80, 100, 150, 200),

limits = c(NA, 200), oob = scales::oob_squish) +

scale_color_manual(values = regimen_colors) +

labs(x = "Renal function (mL/min or mL/min/1.73 m\u00b2)",

y = "Simulated AUC0-24h (mg.h/L, log scale)",

color = "Dosing regimen") +

theme_bw() +

theme(legend.position = "bottom")

Figure_4

tiff("Figure_4.tiff", width = 9, height = 10, units = "in", res = 600,

compression = "lzw", type = "cairo")

print(Figure_4)

dev.off()

```

```{r}

tiff("Figure_4.tiff",

width = 9, height = 10,

units = "in", res = 600,

compression = "lzw",

type = "cairo")

print(Figure_4)

dev.off()

```

```{r}

library(dplyr)

library(tidyr)

library(purrr)

library(pracma)

library(knitr)

\# ============================================================

\# 0) Régimes et simulation

\# ============================================================

regimen_order <- c("450 mg q84h",

"450 mg q48h",

"Half dose (450 mg q24h)",

"Full dose (900 mg q24h)")

regimens <- tibble::tibble(

Regimen = factor(rev(regimen_order), levels = rev(regimen_order)),

amt = c(900, 450, 450, 450),

ii = c(24, 24, 48, 84)

)

simulate_auc_fixed_regimen <- function(pop, amt_dose, ii_dose, model, seed = 123) {

data_ev <- pop %>%

mutate(time = 0, evid = 1, cmt = 1, ss = 1,

amt = amt_dose, ii = ii_dose, addl = 9)

set.seed(seed)

model %>%

data_set(data_ev) %>%

mrgsim(end = 192, delta = 2) %>%

as_tibble() %>%

filter(time >= 0, time <= 168) %>%

group_by(ID) %>%

summarise(AUC_0_24 = trapz(time, DV) / 7, .groups = "drop")

}

find_target_range <- function(pred, grid) {

in_target <- grid[which(pred >= 40 & pred <= 60)]

if (length(in_target) == 0) {

return(tibble(CLcr_min = NA_real_, CLcr_max = NA_real_, continuous = NA))

}

tibble(

CLcr_min = min(in_target),

CLcr_max = max(in_target),

continuous = length(in_target) ==

sum(grid >= min(in_target) & grid <= max(in_target))

)

}

\# ============================================================

\# 1) Analyse CORRIGÉE :

\#	- covariable du modèle = CKD-EPI (DFG_EPI), toujours

\#	- axe x / binning = équation étudiée (X_eq)

\# ============================================================

run_target_range_analysis <- function(full_data, dfg_col, equation_label,

grid = seq(10, 100, by = 0.1)) {

pop_base <- full_data %>%

filter(.data[[dfg_col]] >= 10, .data[[dfg_col]] <= 100) %>%

transmute(ID, WT, CLcr = DFG_EPI, X_eq = .data[[dfg_col]])

results_list <- map_dfr(seq_len(nrow(regimens)), function(i) {

reg <- regimens[i, ]

simulate_auc_fixed_regimen(pop_base %>% select(ID, WT, CLcr),

reg$amt, reg$ii, model_Lalagkas) %>%

left_join(pop_base, by = "ID") %>%

mutate(Regimen = reg$Regimen)

})

results_binned <- results_list %>%

mutate(bin = floor(X_eq)) %>%

group_by(Regimen, bin) %>%

summarise(

median_AUC = median(AUC_0_24, na.rm = TRUE),

q10_AUC	= quantile(AUC_0_24, 0.10, na.rm = TRUE),

q90_AUC	= quantile(AUC_0_24, 0.90, na.rm = TRUE),

.groups = "drop"

)

smoothed <- results_binned %>%

group_by(Regimen) %>%

group_modify(~ tibble(

x = grid,

median_AUC = predict(loess(median_AUC ~ bin, data = .x, span = 0.3), newdata = data.frame(bin = grid)),

q10_AUC	= predict(loess(q10_AUC	~ bin, data = .x, span = 0.3), newdata = data.frame(bin = grid)),

q90_AUC	= predict(loess(q90_AUC	~ bin, data = .x, span = 0.3), newdata = data.frame(bin = grid))

)) %>%

ungroup() %>%

mutate(Equation = equation_label)

target_ranges <- smoothed %>%

group_by(Regimen) %>%

group_modify(~ find_target_range(.x$median_AUC, .x$x)) %>%

ungroup() %>%

mutate(Equation = equation_label, .before = 1)

list(target_ranges = target_ranges, smoothed = smoothed)

}

res_CG	<- run_target_range_analysis(full_data, "DFG_CG", "CG")

res_MDRD <- run_target_range_analysis(full_data, "DFG_MDRD", "MDRD")

res_EPI <- run_target_range_analysis(full_data, "DFG_EPI", "CKD-EPI")

all_target_ranges <- bind_rows(res_CG$target_ranges,

res_MDRD$target_ranges,

res_EPI$target_ranges)

\# ============================================================

\# 2) PANEL A : plages de fonction rénale où la médiane est en cible

\# ============================================================

fmt_range <- function(lo, hi, continuous, grid_min = 10, grid_max = 100) {

ifelse(

is.na(lo), "Never in target (10–100)",

paste0("≈ ", round(lo), "–", round(hi),

ifelse(hi >= grid_max - 0.1, "†", ""),

ifelse(!continuous, "*", ""))

)

}

regimen_labels <- tibble(

Regimen = regimen_order,

`Dosing regimen (SmPC)` = c("450 mg q84h", "450 mg q48h",

"450 mg q24h (half dose)", "900 mg q24h (full dose)"),

`Assigned renal function range` = c("10–24 mL/min", "25–39 mL/min",

"40–59 mL/min", "≥60 mL/min")

)

panel_A <- all_target_ranges %>%

mutate(Regimen = as.character(Regimen),

Range = fmt_range(CLcr_min, CLcr_max, continuous)) %>%

select(Equation, Regimen, Range) %>%

pivot_wider(names_from = Equation, values_from = Range) %>%

right_join(regimen_labels, by = "Regimen") %>%

mutate(Regimen = factor(Regimen, levels = rev(regimen_order))) %>%

arrange(Regimen) %>%

select(`Dosing regimen (SmPC)`, `Assigned renal function range`,

`CG (mL/min)` = CG,

`MDRD (mL/min/1.73m²)` = MDRD,

`CKD-EPI (mL/min/1.73m²)` = `CKD-EPI`)

kable(panel_A, align = "c",

caption = "Supplemental Table 3 — Panel A. Renal function ranges where the median simulated AUC0-24h is within 40–60 mg.h/L, by fixed regimen and equation. † reaches the upper limit of the analysed range (100); * non-continuous range.")

\# ============================================================

\# 3) PANEL B : gaps entre régimes successifs (= seuils RCP)

\# ============================================================

transitions <- tibble(

lower = regimen_order[1:3], # régime le moins intense (CLcr plus basse)

upper = regimen_order[2:4], # régime suivant

Gap = c("Gap 1: q84h → q48h (SmPC threshold 25)",

"Gap 2: q48h → half dose (SmPC threshold 40)",

"Gap 3: half dose → full dose (SmPC threshold 60)")

)

fmt_gap <- function(lo, hi) {

case_when(

is.na(lo) | is.na(hi) ~ "NA (regimen never in target)",

hi > lo	~ paste0("≈ ", round(lo), "–", round(hi),

" (", round(hi - lo), ")"),

TRUE	~ paste0("No gap (overlap ≈ ", round(hi), "–", round(lo), ")")

)

}

make_panel_B_row <- function(tr_eq) {

tr <- tr_eq %>% mutate(Regimen = as.character(Regimen))

gaps <- transitions %>%

left_join(tr %>% select(Regimen, lower_max = CLcr_max), by = c("lower" = "Regimen")) %>%

left_join(tr %>% select(Regimen, upper_min = CLcr_min), by = c("upper" = "Regimen")) %>%

mutate(value = fmt_gap(lower_max, upper_min)) %>%

select(Gap, value)

\# Zones extrêmes : sous le régime le plus faible, au-dessus de la pleine dose

q84_min <- tr$CLcr_min[tr$Regimen == "450 mg q84h"]

full_max <- tr$CLcr_max[tr$Regimen == "Full dose (900 mg q24h)"]

extremes <- tibble(

Gap = c("Below q84h range", "Above full-dose range"),

value = c(

ifelse(!is.na(q84_min) & q84_min > 10.1,

paste0("≈ 10–", round(q84_min), " (", round(q84_min - 10), ")"), "None"),

ifelse(!is.na(full_max) & full_max < 99.9,

paste0("≈ ", round(full_max), "–100 (", round(100 - full_max), ")"), "None")

)

)

bind_rows(gaps, extremes) %>%

pivot_wider(names_from = Gap, values_from = value) %>%

mutate(Equation = unique(tr_eq$Equation), .before = 1)

}

panel_B <- all_target_ranges %>%

group_split(Equation) %>%

map_dfr(make_panel_B_row) %>%

mutate(Equation = factor(Equation, levels = c("CG", "MDRD", "CKD-EPI"))) %>%

arrange(Equation)

kable(panel_B, align = "c",

caption = "Supplemental Table 3 — Panel B. Gap zones where no fixed regimen achieves a median AUC0-24h of 40–60 mg.h/L (range, width in mL/min or mL/min/1.73m²).")

```

```{r}

auc_by_id_CG %>%

mutate(side = case_when(

CLcr >= 59 & CLcr < 60 ~ "59–60 (half dose)",

CLcr >= 60 & CLcr < 61 ~ "60–61 (full dose)"

)) %>%

filter(!is.na(side)) %>%

count(side, Exposure) %>%

group_by(side) %>%

mutate(pct = round(100 * n / sum(n), 1)) %>%

ungroup()

```

```{r}

library(dplyr)

library(purrr)

classify <- function(auc) cut(auc, c(-Inf, 40, 60, Inf), right = FALSE,

labels = c("Underexposure", "Target attainment", "Overexposure"))

\# Zone sans régime adéquat (CG), d'après la Supplemental Table 3

gap_lo <- 49; gap_hi <- 67

pop_gap <- full_data %>%

filter(DFG_CG >= gap_lo, DFG_CG < gap_hi) %>%

transmute(ID, WT, CLcr = DFG_EPI)	# covariable du modèle = CKD-EPI

gap_res <- map_dfr(list(c(450, 24), c(900, 24)), function(d) {

simulate_auc_fixed_regimen(pop_gap, d[1], d[2], model_Lalagkas) %>%

mutate(Regimen = ifelse(d[1] == 450, "Half dose (450 mg q24h)", "Full dose (900 mg q24h)"),

Exposure = classify(AUC_0_24))

})

gap_tab <- gap_res %>%

count(Regimen, Exposure) %>%

group_by(Regimen) %>%

mutate(pct = round(100 * n / sum(n), 1)) %>%

ungroup()

print(as.data.frame(gap_tab))

```
